# ESCRT-III assembly restores membrane tension in micron-scale plasma membrane repair

**DOI:** 10.1083/jcb.202601076

**Published:** 2026-11-13

**Authors:** Xinan Meng, Yuanyuan Wang, Erqing Gao, Liwei Guan, Yiyuan Ge, Jingyi Kang, Jingxiu Xu, Jingyang Yuan, Shujun Cai, Eric Miska, Wei Yang, Suhong Xu

**Affiliations:** 1 Cluster of Genetic, Development, and Regeneration and Department of Burn and Wound Repair of the Second Affiliated Hospital, Zhejiang University School of Medicine, Hangzhou, China; 2 School of Basic Medical Sciences and Forensic Medicine, Hangzhou Medical College, Hangzhou, China; 3 Centre for Cellular Biology and Signaling, Zhejiang University-University of Edinburgh Institute, Zhejiang University, Haining, China; 4 Department of Neurology, Center for Membrane Receptor and Brain Medicine, the Fourth Affiliated Hospital of School of Medicine, and International School of Medicine, International Institutes of Medicine, Zhejiang University, Yiwu, China; 5 Biomedical Sciences, College of Medicine and Veterinary Medicine, University of Edinburgh, Edinburgh, UK; 6 Department of Chemical Biology, School of Life Sciences, Southern University of Science and Technology, Shenzhen, China; 7 Institute for Biological Electron Microscopy, Southern University of Science and Technology, Shenzhen, China; 8 Department of Biochemistry, University of Cambridge, Cambridge, UK

## Abstract

Plasma membrane integrity is essential for cellular survival, yet how cells repair micron-scale plasma membrane ruptures remains poorly understood. Here, using the genetically tractable *Caenorhabditis elegans* epidermis, we combine live imaging, genetics, super-resolution microscopy, and biophysical approaches to define a size-dependent role for endosomal sorting complexes required for transport (ESCRT)-III in large-scale membrane repair. Small wounds induce transient, focal VPS-32 accumulation, whereas large wounds trigger the formation of a persistent but dynamic, circumferential ESCRT-III assembly that remains associated with the wound during repair. Formation of this assembly requires conserved ESCRT regulators, including ALX-1, TSG-101, and VPS-4, together with VPS-32 polymerization. The wound-associated ESCRT-III assembly is required for membrane tension restoration and efficient membrane repair, while disrupting VPS-32 polymerization impairs tension recovery, reduces vesicle recruitment and wound closure, and consequently compromises axon regeneration and organismal survival following injury. Together, our findings suggest that large plasma membrane wounds induce a persistent, dynamic ESCRT-III assembly that restores membrane tension and facilitates vesicle-mediated membrane addition to promote wound repair.

## Teaser

Plasma membrane wounds of different sizes elicit distinct ESCRT-III responses. Using the *C. elegans* epidermis, Xu et al. show that micron-scale wounds induce a persistent but dynamic ESCRT-III assembly that promotes membrane tension restoration and vesicle recruitment, thereby facilitating efficient membrane repair and tissue recovery.

## Introduction

Plasma membrane integrity is essential for cellular viability and for the maintenance, architecture, and function of tissues (Ammendolia et al., 2021). This barrier is continually challenged by physiological activities, including cell migration, contractility, and tissue remodeling, as well as by mechanical stress from the external environment. Membrane disruptions must be repaired rapidly, as delayed resealing can lead to uncontrolled Ca^2+^ influx, osmotic imbalance, and ultimately cell death (Cooper and McNeil, 2015). Efficient membrane repair is therefore a fundamental determinant of organismal resilience and tissue homeostasis (Tang and Marshall, 2017).

The demand for rapid and robust repair is especially pronounced in mechanically active tissues. Large-scale membrane disruptions accompany diverse pathological states, including the repeated tearing of myofibers in muscular dystrophies, widespread damage during ischemia-reperfusion injury in stroke or myocardial infarction, and acute mechanical trauma incurred during wound healing and nerve regeneration (McNeil and Kirchhausen, 2005). In these contexts, cells must mobilize repair machinery capable of acting with sufficient speed, mechanical robustness, and spatial coordination to seal micron-scale lesions. How this is achieved *in vivo* remains an important unresolved question in regenerative cell biology (McNeil and Steinhardt, 2003).

The endosomal sorting complexes required for transport (ESCRT) machinery has emerged as a central regulator of membrane remodeling and repair (Burigotto and Carlton, 2026; Park et al., 2024; Vietri et al., 2020; Hurley et al., 2025; Gatta and Carlton, 2019). In particular, the ESCRT-III module, typified by CHMP4B, assembles into membrane-associated filaments that drive membrane deformation, constriction, and scission during diverse cellular processes, including multivesicular body formation, cytokinetic abscission, nuclear envelope repair, and plasma membrane repair (Vietri et al., 2020; Gatta and Carlton, 2019; Hurley and Emr, 2006; Olmos, 2022). Recent studies have revealed that ESCRT-III assemblies exhibit remarkable structural and functional plasticity, forming diverse higher-order architectures that are continuously remodeled by the AAA + ATPase VPS4 (Harker-Kirschneck et al., 2019; Pfitzner et al., 2020; Stempels et al., 2023; Bertin et al., 2020; Shen et al., 2014; Tang et al., 2015; Jukic et al., 2023). Despite these advances, most studies of ESCRT-mediated plasma membrane repair have focused on relatively small membrane lesions, where ESCRT-III is thought to facilitate rapid membrane sealing and removal.

Whether ESCRT-III performs additional functions during the repair of large plasma membrane wounds remains unclear. In particular, it is not known how ESCRT-III responds to the substantial membrane tension changes and mechanical challenges associated with micron-scale membrane (Pontes et al., 2017; Raj et al., 2024; Togo et al., 1999). Recent studies have shown that tetraspanins assemble into ring-like structures at the wound margins, where they limit wound expansion and constrain edge retraction, thereby facilitating the early stage of repair (Huang et al., 2022; Wang et al., 2022). Works in multiple systems have also highlighted an intimate relationship between membrane mechanics and ESCRT-III activity (Lafaurie-Janvore et al., 2013; Booth et al., 2019), suggesting that ESCRT-III assemblies can both respond to and influence membrane tension.

The epidermis of *Caenorhabditis elegans* provides a powerful *in vivo* model for investigating these questions. Previous studies have shown that epidermal wounding triggers rapid Ca^2+^ signaling, mitochondrial response, and cytoskeletal reorganization that are essential for wound closure (Xu and Chisholm, 2011; Xu and Chisholm, 2014; Xu et al., 2021; Fu et al., 2020). More recent work has demonstrated that Golgi-derived vesicles, the SNARE protein SYX-2, and the fusogen EFF-1 contribute to large-wound repair (Wijaya and Xu, 2024; Meng et al., 2020; Shao et al., 2023; Meng et al., 2023), supporting an important role for vesicle-mediated membrane remodeling during restoration of epidermal integrity. However, how ESCRT-III participates in coordinating these events remains unknown.

Here, we show that large plasma membrane wounds induce the formation of a persistent, multicomponent ESCRT-III assembly at the wound perimeter. Unlike the transient ESCRT-III assemblies associated with small wounds, these structures persist for several hours, exhibit reduced molecular turnover, and depend on conserved ESCRT-III regulators, including ALIX and VPS4. We further find that VPS-32 polymerization is required for membrane tension recovery, efficient vesicle recruitment, and successful wound closure. Together, our findings reveal a wound size–dependent ESCRT-III response and support a model in which persistent and dynamic ESCRT-III assemblies help to coordinate membrane stabilization, tension restoration, and vesicle-mediated repair during recovery from large plasma membrane damage.

## Results

### VPS-32 is recruited to plasma membrane wounds in a size-dependent manner

**Figure 1.**
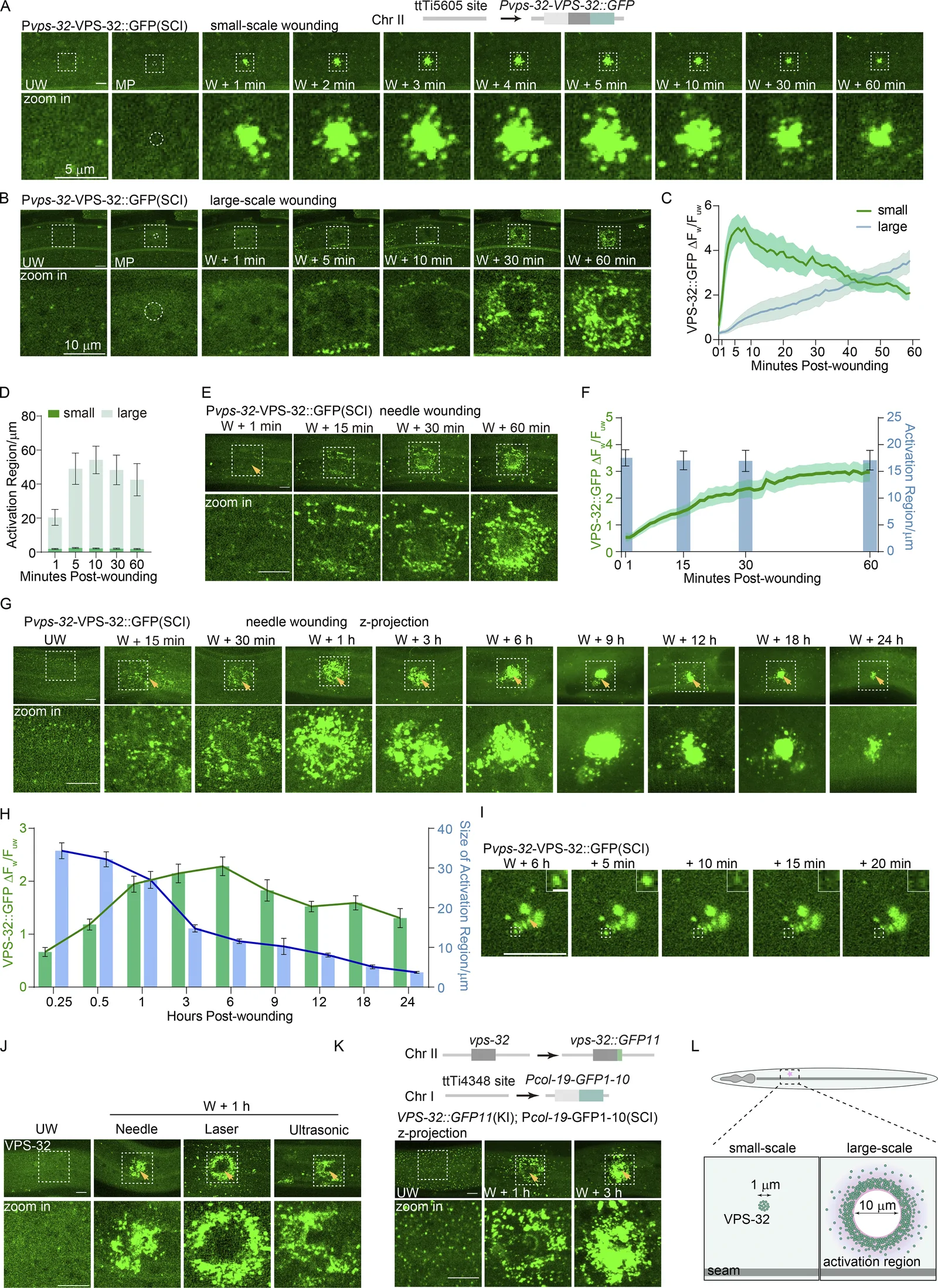
**Wounding triggers the size-dependent recruitment of VPS-32. (A)** Top, schematic of the CRISPR-Cas9 strategy to generate the single-copy *Pvps-32-VPS-32::GFP*(*zjuSi429*) reporter in *C. elegans*. Bottom, representative time-lapse confocal images of the recruitment of VPS-32::GFP at the indicated time points after small-scale laser-induced wounding in *C. elegans* epidermal cells. *Pvps-32-VPS-32::GFP(zjuSi429)* transgenic animals were used. The circle indicates the wound site. Scale bar, 5 μm. See Video 1. **(B)** Representative time-lapse confocal images of the recruitment of VPS-32::GFP at the indicated time points after large-scale laser-induced wounding in *C. elegans* epidermal cells. *Pvps-32-VPS-32::GFP(zjuSi429)* transgenic animals were used. The circle indicates the wound site. Scale bar, 10 μm. See Video 2. **(C)** Quantification of VPS-32::GFP fluorescence intensity within wound regions over time after wounding for small-scale (green) and large-scale (cyan) wounds. Data are presented as mean ± SEM (*n* = 10 and 10, respectively). **(D)** Quantification of the region size of VPS-32 accumulation after small (*n* = 15, 15, 15, 14, and 10, respectively) and large-scale laser wounds (*n* = 8, 8, 8, 8, and 8, respectively) at the indicated time points. Data are presented as mean ± SEM. **(E)** Representative confocal images showing the recruitment of VPS-32::GFP to wound sites in *C. elegans* epidermal cells at the indicated time points after needle-induced wounding. *Pvps-32-VPS-32::GFP(zjuSi429)* transgenic animals were used. Scale bar, 10 μm. See Video 3. **(F)** Quantification of VPS-32::GFP fluorescence intensity within wound regions over time after needle-induced wounding. Data are presented as mean ± SEM (*n* = 17). **(G)** Representative time-lapse confocal images showing the recruitment of VPS-32::GFP to needle-induced wound sites in *C. elegans* epidermal cells at the indicated times after needle-induced wounding. Arrows indicate wound sites. Scale bar, 10 μm. **(H)** Quantification of VPS-32::GFP fluorescence intensity and the sizes of VPS-32 activation regions over time after needle-induced wounding. Data are presented as mean ± SEM (*n* = 73, 59, 53, 66, 57, 37, 138, 46, and 30, respectively). **(I)** Representative time-lapse confocal images showing the disappearance of VPS-32::GFP at wound site 6 h after needle-induced wounding. Arrows indicate wound sites. Scale bar, 10 μm; zoom-in scale bar, 1 μm. See Video 4. **(J)** Confocal images showing the recruitment of VPS-32::GFP to wound sites in *C. elegans* epidermal cells by needle, laser, or ultrasonic wounding assays. *Pvps-32-VPS-32::GFP*(*zjuSi429*) transgenic animals were used. Arrows indicate wound sites. Scale bar, 10 μm. **(K)** Top, schematic of the split-GFP strategy used for endogenous, epidermis-specific labeling of VPS-32. VPS-32::GFP11 was generated by CRISPR-Cas9 knock-in and visualized by epidermal expression of sfGFP1-10. Bottom, representative confocal images showing the recruitment of VPS-32::GFP to wound sites at the indicated times after needle-induced wounding. Animals carrying *vps-32::GFP11(zju284); Pcol-19-sfGFP1-10(zjuSi177)* alleles were used. Arrows indicate wound sites. Scale bar, 10 μm. **(L)** Schematic illustrating the distinct VPS-32 recruitment pattern induced by small and large plasma membrane wounds. The inset shows a magnified view of the wound core and surrounding VPS-32 activation regions.

To determine how the ESCRT-III machinery responds to plasma membrane damage of different magnitudes, we used *C. elegans* hyp7 epidermis, a large multinucleated syncytium that provides an excellent *in vivo* model for membrane repair following tissue injury (Meng et al., 2020). Membrane damage was induced using three independent approaches: Micropoint UV laser ablation, needle puncture, or ultrasound-mediated rupture to distinguish general wound responses from injury method-specific effects. VPS-32, the *C. elegans* ortholog of CHMP4B, was visualized using a single-copy insertion (SCI) transgene expressing *Pvps-32*-VPS-32::GFP, thereby preserving physiological expression levels (Fig. 1 A).

In unwounded animals, VPS-32::GFP was distributed diffusely throughout the cytoplasm with no obvious plasma membrane association (Fig. 1 A). Small laser-induced membrane lesions (∼1 μm) triggered a rapid recruitment of VPS-32::GFP puncta to the wound site, where discrete puncta became detectable within seconds after injury (Fig. 1 A and Video 1). VPS-32::GFP fluorescence peaked within 5 min, remaining confined to a small region (∼5 μm^2^) surrounding the lesion (Fig. 1, A, C, and D). The puncta progressively increased in size and intensity before diminishing by 30 min after wounding (Fig. 1, A and C). This transient and highly localized response is consistent with rapid ESCRT-III assembly that facilitates excision of small, locally damaged membrane (Jimenez et al., 2014; Scheffer et al., 2014).

**Video 1.** **VPS-32 dynamics following small laser-induced wounding.** Time-lapse spinning-disk confocal microscopy of VPS-32::GFP (green) in the epidermis of a young adult *C. elegans* carrying the SCI *Pvps-32-VPS-32::GFP(zjuSi429)*. A Micropoint UV laser was used to generate a small (2–3 μm) plasma membrane wound. Images were acquired at 1-min intervals and are played back at 6 frames/s. Elapsed time after wounding (HH:mm:ss) is displayed in the lower-right corner. Scale bar, 10 μm. Related to Fig. 1 A.

In contrast, large laser-induced wounds (∼10 μm) elicited a markedly different pattern of VPS-32::GFP recruitment. Shortly after injury, numerous VPS-32::GFP puncta emerged throughout a broad peri-wound region extending tens of micrometers from the lesion (Fig. 1, B–D and Video 2). Time-lapse imaging revealed that these puncta were highly dynamic, undergoing repeated cycles of movement, disappearance, fusion, and coalescence while progressively accumulating around the wound margin (Fig. 1, B–D and Video 2). Rather than remaining confined to the immediate lesion, VPS-32 gradually assembled into a prominent circumferential structure surrounding the wound that persisted for more than 1 h (Video 2). Thus, unlike the transient focal accumulation observed at small wounds, large wounds induced the formation of a long-lived wound-associated ESCRT-III assembly.

**Video 2.** **VPS-32 dynamics following large laser wounding.** Time-lapse spinning-disk confocal microscopy of VPS-32::GFP (green) in the epidermis of a young adult *C. elegans* carrying *Pvps-32-VPS-32::GFP(zjuSi429)*. A Micropoint UV laser was used to generate a large (∼10 μm) plasma membrane wound. Images were taken at 30-s intervals and are played back at 10 frames/s; elapsed time after wounding (HH:mm:ss) is displayed in the lower-right corner. Scale bar, 10 μm. Related to Fig. 1 B.

A similar recruitment pattern was observed following needle puncture, although the wound-associated VPS-32 assembly persisted considerably longer, remaining detectable for 6 and 24 h before gradually declining (Fig. 1, E–I; and Videos 3 and 4). Ultrasound-induced membrane rupture likewise triggers a robust VPS-32 accumulation pattern at wound sites (Fig. 1 J), indicating that this response represents a general feature of large plasma membrane injuries rather than a consequence of a specific wound method. Notably, the dynamic VPS-32 puncta observed in the peri-wound region resemble the directional ESCRT-III puncta waves recently reported for CHMP2B during plasma membrane repair in mammalian cells (Heffner et al., 2026), suggesting that directional ESCRT-III recruitment toward membrane wounds is evolutionarily conserved.

**Video 3.** **VPS-32 dynamics following needle-induced wounding.** Time-lapse spinning-disk confocal microscopy of VPS-32::GFP (green) in the epidermis of a young adult *C. elegans* carrying *Pvps-32-VPS-32::GFP(zjuSi429)*, wounded by manual puncture with a hand-pulled glass needle. Images were acquired at 1 min intervals and are played back at 6 frames/s. Elapsed time after wounding (HH:mm:ss) is displayed in the lower-right corner. Scale bar, 10 μm. Related to Fig. 1 E.

**Video 4.** **Long-term dynamics and disassembly of wound-associated VPS-32 starting 6 h after needle-induced wounding.** Time-lapse spinning-disk confocal microscopy of VPS-32::GFP (green) in the epidermis of a young adult *C. elegans* carrying *Pvps-32-VPS-32::GFP(zjuSi429)*. Images were acquired every 10 s and are played back at 12 frames/s. Elapsed time after wounding (HH:mm:ss) is displayed in the lower-right corner. Scale bar, 10 μm. Related to Fig. 1 I.

To determine whether the observed dynamic reflects endogenous VPS-32 behavior, we generated a split-GFP knock-in strain by inserting a GFP11 into the endogenous *vps-32* locus and expressing GFP1-10 specifically in hyp7 epidermal cells (Fig. 1 K). Reconstituted GFP fluorescence faithfully recapitulated both the timing and spatial distribution of VPS-32 recruitment observed using the SCI VPS-32::GFP reporter following both laser- and needle-induced wounding (Fig. 1, B, E, and J), confirming that the transgenic reporter accurately reflects endogenous VPS-32 behavior. Together, these data demonstrate that ESCRT-III recruitment is strongly wound-size dependent: small membrane lesions induce rapid and transient VPS-32 accumulation, whereas large membrane disruption triggers the progressive assembly of a persistent wound-associated ESCRT-III structure (Fig. 1 L). These observations suggest that ESCRT-III adopts distinct organizational states depending on the magnitude of membrane damage.

### Large wounds recruit the ESCRT-III machinery through canonical upstream regulators

**Figure 2.**
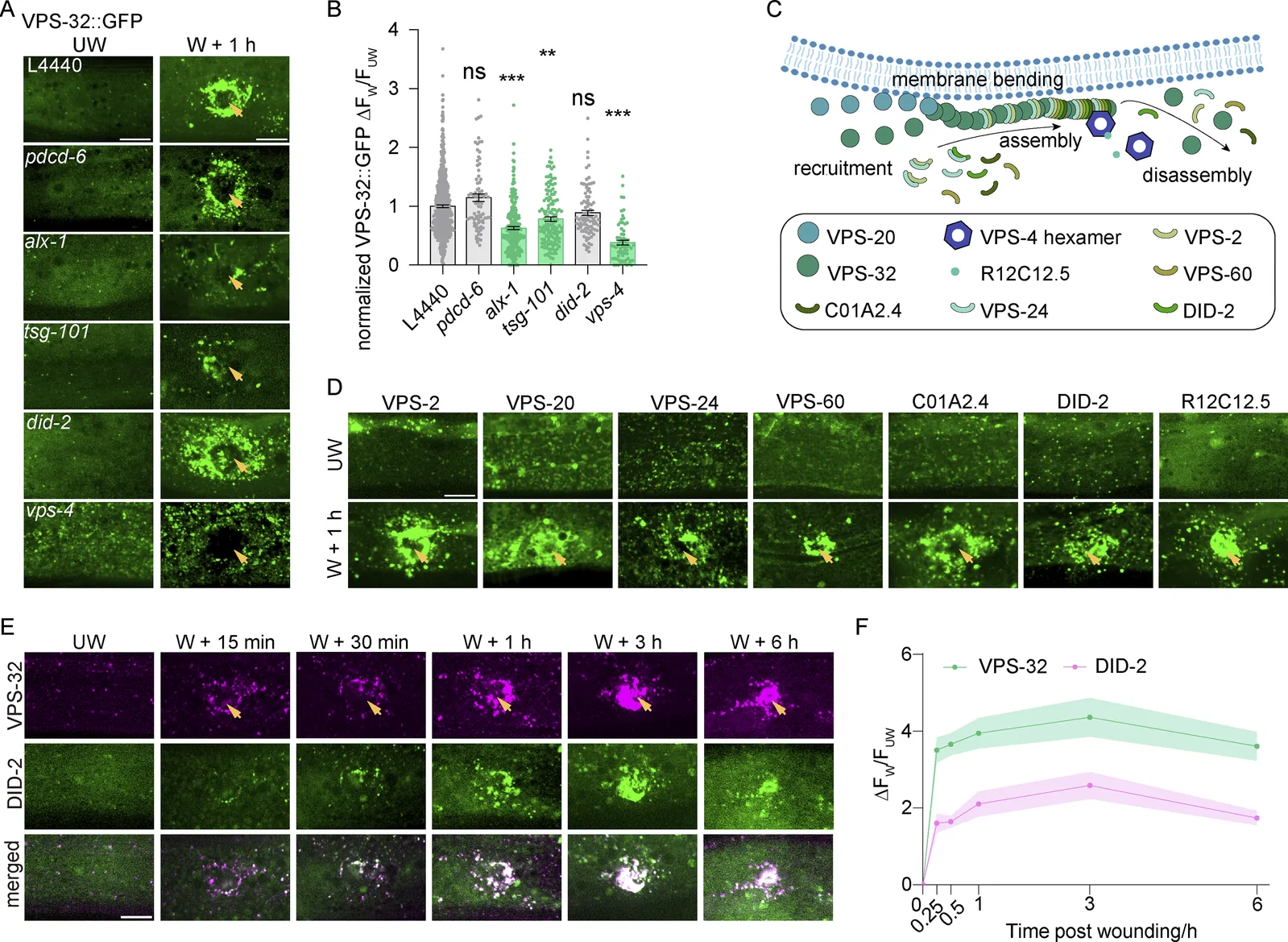
**Large plasma membrane wounds recruit the ESCRT-III machinery through canonical upstream regulators. (A)** Representative confocal images of VPS-32::GFP localization before and 1 h after needle-induced wounding under the RNAi treatment of L4440 control, *pdcd-6*, *alx-1*, *tsg-101*, *did-2,* and *vps-4*. *Pvps-32-VPS-32::GFP(zjuSi429)* transgenic animals were used. Scale bar, 10 μm. **(B)** Quantification of VPS-32::GFP fluorescence intensity within wound regions over time after wounding for the RNAi treatment of L4440 control, *pdcd-6*, *alx-1*, *tsg-101*, *did-2*, and *vps-4*. Data are presented as mean ± SEM (*n* = 589, 82, 225, 143, 89, and 69 from left to right). Statistical significance was assessed by the Kruskal–Wallis test, **P < 0.01, ***P < 0.001, ns, not significant. **(C)** Schematic illustrating the hierarchical assembly and VPS-4–dependent remodeling and disassembly of the ESCRT-III machinery. **(D)** Representative confocal images of the recruitment of various ESCRT-III components (VPS-2, VPS-20, VPS-24, VPS-60, C01A2.4, DID-2, and R12C12.5) to wound sites at 1 h after needle-induced wounding. *Pcol-19-VPS-2::GFP(zjuEx2393)*, *Pcol-19-VPS-20::GFP(zjuEx2367), Pcol-19-VPS-24::GFP(zjuEx2403), Pcol-19-VPS-60::GFP(zjuEx2530), Pcol-19-C01A2.4::GFP(zjuEx2545), Pcol-19-DID-2::GFP(zjuEx2397)*, and *Pcol-19-R12C12.5::GFP(zjuEx2543)* transgenic animals were used. Scale bar, 10 μm. **(E)** Representative confocal images of VPS-32::tagBFP and DID-2::GFP before and at different time points after needle-induced wounding. *Pdid-2-DID-2::GFP(zjuSi447); Pvps-32-VPS-32::tagBFP(zjuSi457)* transgenic animals were used. Scale bar, 10 μm. **(F)** Quantification of VPS-32::tagBFP (*n* = 38, 50, 21, 27, and 30, respectively) and DID-2::GFP (*n* = 23, 25, 27, 20, and 25, respectively) fluorescence intensity within wound regions over time after needle-induced wounding. Data are presented as mean ± SEM.

Previous studies in mammalian cells have shown that recruitment of CHMP4B/VPS-32 to plasma membrane or lysosome membrane lesions depends on upstream ESCRT-III components (Radulovic et al., 2018; Shukla et al., 2024; Scheffer et al., 2014; Skowyra et al., 2018; Goul et al., 2026). We therefore asked whether large plasma membrane wounds employed a similar recruitment pathway in *C. elegans*. Using epidermal-specific RNAi, we depleted several conserved ESCRT regulators, including *alx-1* (ALIX), *tsg-101* (TSG101), *pdcd-6/M04F3.4* (PDCD6/ALG2), *did-2* (DID2), and *vps-4*, in animals expressing VPS-32::GFP. Depletion of *alx-1* or *tsg-101* markedly reduced VPS-32 accumulation at the wound periphery, resulting in a significant decrease in fluorescence intensity and extent of the wound-associated VPS-32 assembly (Fig. 2, A and B). These findings indicate that large-wound repair utilizes the conserved ALIX and TSG101 that act upstream of ESCRT-III during repair of small plasma membrane injuries.

Notably, depletion of *vps-4* substantially impaired VPS-32 accumulation at wound sites (Fig. 2, A and B), indicating that VPS4-dependent ESCRT-III remodeling is required not only for disassembly of ESCRT-III filaments but also for efficient assembly of the wound-associated VPS-32 structure. In contrast, depletion of *pdcd-6* or ESCRT-III subunit *did-2* had little effect on VPS-32 recruitment (Fig. 2, A and B). Interestingly, the dispensability of PDCD-6/ALG2 parallels observations in lysosomal membrane repair, where PDCD-6/ALG2–independent ESCRT-III recruitment has also been reported (Chen et al., 2024; Corkery et al., 2026; Skowyra et al., 2018). Together, these observations suggest that although large membrane repair utilizes core ESCRT recruitment pathways, it may also engage distinct regulatory mechanisms, potentially reflecting the different geometric and mechanical challenges associated with membrane disruptions.

**Figure S1.**
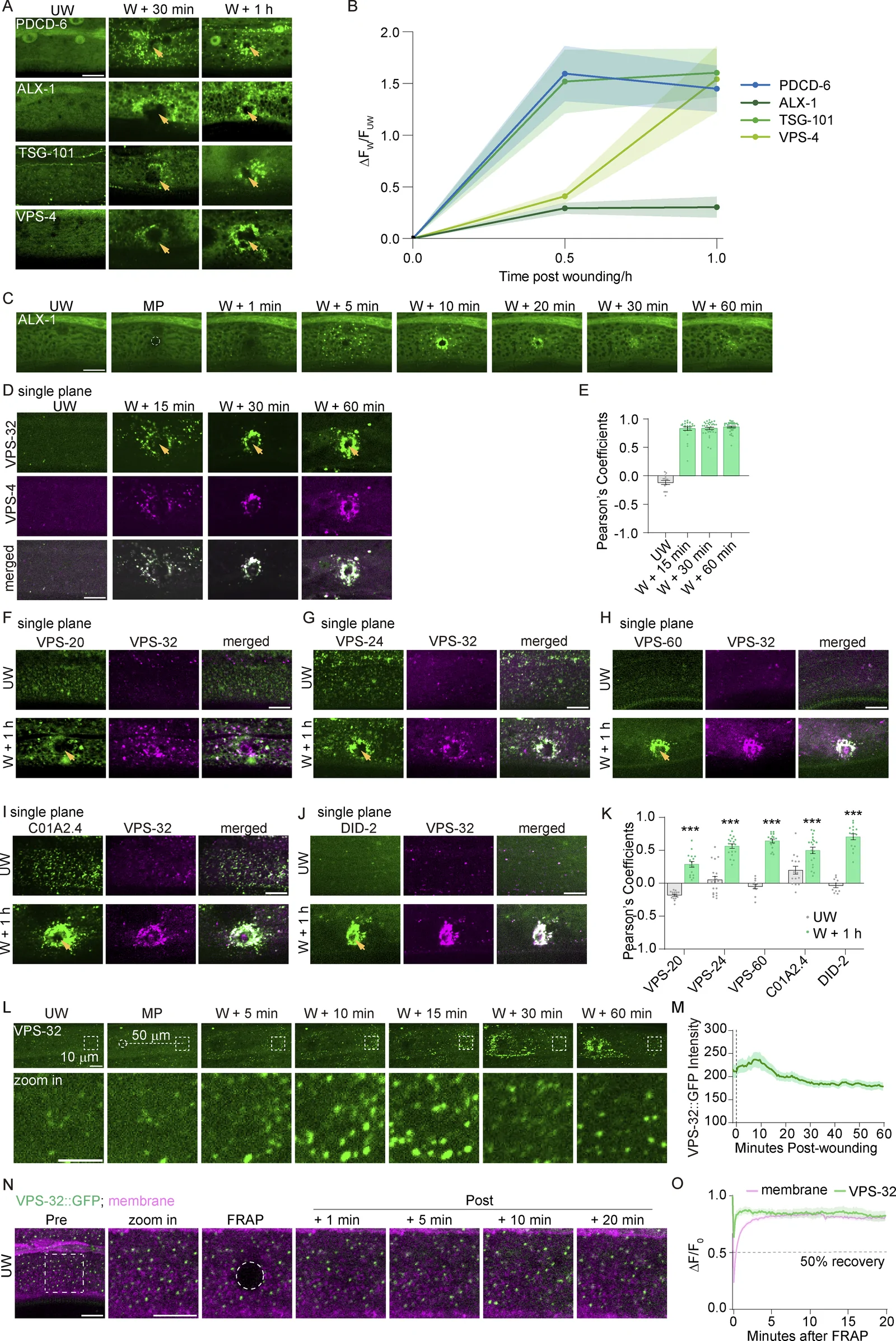
**Recruitment of upstream ESCRT components and ESCRT-III subunits to plasma membrane wounds. (A)** Representative time-lapse confocal images showing recruitment of PDCD-6::GFP, ALX-1::GFP, TSG-101::GFP, and VPS-4::mKate2 to needle-induced wound sites at 0.5 and 1 h after wounding. *Pcol-19-PDCD-6::GFP(zjuSi435)*, *Pcol-19-GFP::ALX-1(zjuSi437)*, *Pvps-4-VPS-4::mKate2(zjuSi478)*, and *Pcol-19-TSG-101::GFP(zjuEx2402)* transgenic animals were used. Arrows indicate wound sites. Scale bar, 10 μm. **(B)** Quantification of fluorescence intensity of the indicated upstream ESCRT components (PDCD-6::GFP [*n* = 21 and 24], ALX-1::GFP [*n* = 27 and 20], TSG-101::GFP [*n* = 21 and 20], andVPS-4::mKate2 [*n* = 40 and 43]) at wound sites over time after needle-induced wounding. Data are presented as mean ± SEM. **(C)** Representative time-lapse confocal images showing recruitment of ALX-1::GFP after laser-induced wounding. *Pcol-19-GFP::ALX-1(zjuSi437)* transgenic animals were used. The circle indicates the wound site. Scale bar, 10 μm. **(D)** Representative time-lapse confocal images showing recruitment of VPS-32::GFP and VPS-4::mKate2 after needle-induced wounding. *Pvps-32-VPS-32::GFP(zjuSi443); Pvps-4-VPS-4::mKate2(zjuSi478)* transgenic animals were used. Arrows indicate the wound site. Scale bar, 10 μm. **(E)** Pearson’s correlation coefficients quantifying colocalization between VPS-4::mKate2 and VPS-32::GFP before and 15 min, 30 min, and 1 h after needle-induced wounding. Data are presented as mean ± SEM (*n* = 14, 26, 32, and 37, respectively). **(F–J)** Representative confocal images showing recruitment of the ESCRT-III subunits VPS-20::GFP (F), VPS-24::GFP (G), VPS-60::GFP (H), C01A2.4::GFP (I), DID-2::GFP (J) together with VPS-32::tagBFP at 1 h after needle-induced wounding. Animals expressing *Pvps-32-VPS-32::tagBFP(zjuSi457)* together with the indicated GFP-tagged ESCRT-III reporter were used. Arrows indicate wound sites. Scale bar, 10 μm. **(K)** Pearson’s correlation coefficients quantifying colocalization between VPS-32::tagBFP and the indicated ESCRT-III subunits before and 1 h after needle-induced wounding. Data are presented as mean ± SEM (*n* = 16, 16, 20, 18, 10, 16, 15, 20, 10, and 15 from left to right). Statistical significance was assessed by the Mann–Whitney test, ***P < 0.001. **(L)** Representative time-lapse confocal images showing transient recruitment of VPS-32::GFP to regions ∼50 μm from large laser-induced wounds in *C. elegans* epidermal cells. Images were taken at the indicated time points after wounding. *Pvps-32-VPS-32::GFP(zjuSi429)* transgenic animals were used. The circle indicates the wound site. Scale bar, 10 μm; zoom-in scale bar, 5 μm. **(M)** Quantification of VPS-32::GFP fluorescence intensity in regions located 50 μm from the wound edge over time after large laser-induced wounding. Data are presented as mean ± SEM (*n* = 10). **(N)** Representative time-lapse confocal images of FRAP of VPS-32::GFP and membrane marker myr::mKate2 in the unwounded region of epidermal cells. Images were acquired before photobleaching (Pre), immediately after photobleaching (0 min), and at indicated recovery time points after bleaching (Post). *Pcol-19-myr::mKate2(zjuSi46); Pvps-32-VPS-32::GFP(zjuSi443)* transgenic animals were used. Scale bar, 10 μm. See Video 8. **(O)** FRAP recovery curve of VPS-32::GFP and myr::mKate2 measured in the unwounded region. Data are presented as mean ± SEM (*n* = 9). The dashed line indicates 50% fluorescence recovery.

To determine whether upstream ESCRT components are themselves recruited to large plasma membrane wounds, we generated fluorescent reporters for ALX-1, PDCD-6, and TSG-101. Live imaging revealed that all these proteins rapidly accumulated at wound sites following injury (Fig. S1, A–C), demonstrating coordinated activation of upstream ESCRT machinery during large membrane injury. Because VPS-4 is the ATPase responsible for remodeling and recycling ESCRT-III assemblies (Burigotto and Carlton, 2026), we next examined its localization after wounding. Dual-color imaging of VPS-32::GFP and VPS-4::mKate2 showed that VPS-4 was robustly recruited to wound sites and colocalized with VPS-32 throughout the repair process (Fig. S1, D and E). Together, these results demonstrate that large plasma membrane injury triggers coordinated recruitment of both upstream ESCRT regulators and VPS-4, supporting a model in which ALX-1, TSG-101, and VPS-4–dependent ESCRT activity promotes efficient assembly and remodeling of the wound-associated ESCRT-III structure.

We next asked whether additional ESCRT-III components are recruited to wound sites. Fluorescently tagged VPS-2, VPS-20, VPS-24, VPS-60, C01A2.4, DID-2, and R12C12.5 all accumulated at the wound periphery following injury (Fig. 2, C and D), indicating that large plasma membrane wounds recruit multiple ESCRT-III components rather than VPS-32 alone. Moreover, these ESCRT-III components exhibited a varying degree of colocalization with VPS-32 at the wound margin 1 h after injury (Fig. S1, F–K). Time-lapse images further revealed that DID-2::GFP recruitment occurs more slowly than VPS-32::tagBFP (Fig. 2, E and F), consistent with sequential assembly of ESCRT-III subcomplexes (Pfitzner et al., 2020; Pfitzner et al., 2021). Collectively, these results demonstrate that large plasma membrane wounds activate a broad ESCRT response involving both canonical upstream regulators and multiple ESCRT-III subunits, resulting in the coordinated assembly of a wound-associated ESCRT-III structure.

### VPS-32 assembles into a dynamic and persistent structure at the wounded membrane

**Figure 3.**
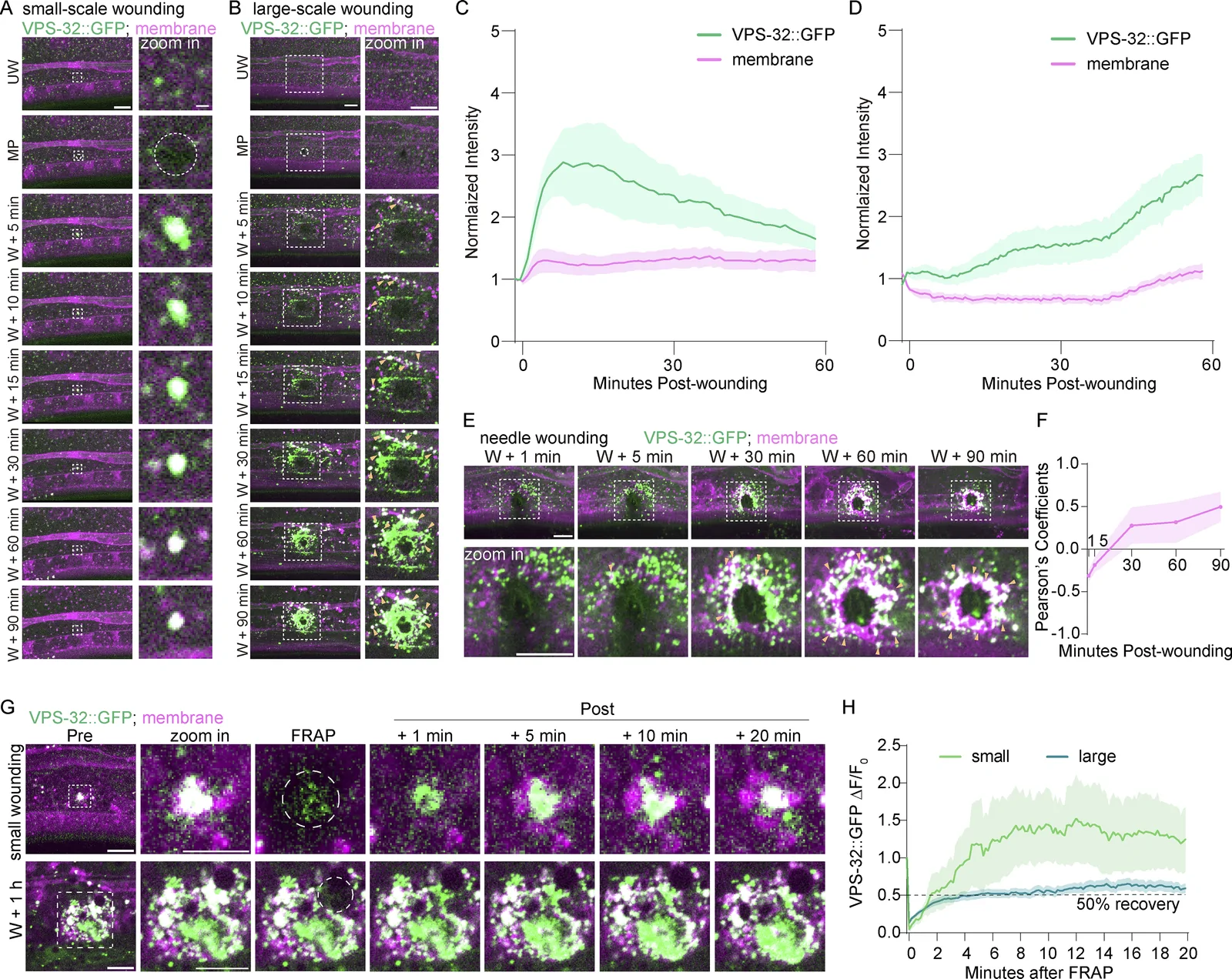
**VPS-32 forms a persistent and dynamically wound-associated ESCRT-III assembly. (A)** Representative time-lapse confocal images showing VPS-32::GFP recruitment at the indicated time points to small-scale laser-induced wounds, colocalized with the membrane marker myr::mKate2. *Pcol-19-myr::mKate2(zjuSi46); Pvps-32-VPS-32::GFP(zjuSi443)* transgenic animals were used. The circle indicates the wound site. Scale bar,10 μm; zoom-in scale bar, 1 μm. See Video 5. **(B)** Representative time-lapse confocal images showing VPS-32::GFP recruitment at the indicated time points to large-scale laser-induced wounds, colocalized with the membrane marker myr::mKate2. *Pcol-19-myr::mKate2(zjuSi46); Pvps-32-VPS-32::GFP(zjuSi443)* transgenic animals were used. The circle indicates the wound site. Arrowheads indicate sites of colocalization. Scale bar, 10 μm. See Video 6. **(C)** Quantification of normalized fluorescence intensity of VPS-32::GFP (*n* = 8) and myr::mKate2 (*n* = 7) at small laser-induced wounds over time. Data are presented as mean ± SEM. **(D)** Quantification of normalized fluorescence intensity of VPS-32::GFP (*n* = 5) and myr::mKate2 (*n* = 8) at large laser-induced wounds over time. Data are presented as mean ± SEM. **(E)** Representative time-lapse confocal images showing VPS-32::GFP recruitment at the indicated time points to needle-induced wound sites together with plasma membrane marker myr::mKate2. *Pcol-19-myr::mKate2(zjuSi46); Pvps-32-VPS-32::GFP(zjuSi443)* transgenic animals were used. Arrowheads indicate sites of colocalization. Scale bar, 10 μm. See Video 7. **(F)** Quantification of the Pearson’s correlation coefficients between VPS-32::GFP and myr::mKate2 over time after needle-induced wounding. Data are presented as mean ± SEM (*n* = 5). **(G)** Representative time-lapse confocal images showing the FRAP of VPS-32::GFP at small laser-induced wounds and needle-induced (large) wounds (1 h after wounding). *Pcol-19-myr::mKate2(zjuSi46); Pvps-32-VPS-32::GFP(zjuSi443)* transgenic animals were used. Images were taken before bleaching (Pre), immediately after bleaching (FRAP), and at the indicated times after bleaching (Post). Scale bar, 10 μm. See Videos 9 and 10. **(H)** Quantification of VPS-32::GFP and myr::mKate2 fluorescence recovery following FRAP at a small laser-induced wound site (*n* = 5) and needle-induced large wounds (*n* = 8). Data are presented as mean ± SEM. The 50% recovery level is indicated for reference.

To further characterize the organization of VPS-32 during plasma membrane repair, we co-expressed VPS-32::GFP with the plasma membrane marker myr::mKate2 (Fig. 3, A and B). Because homozygous GFP tagging of endogenous VPS-32 caused lethality, all subsequent analyses were performed using the single-copy *Pvps-32*-VPS-32::GFP transgene, which showed physiological expression and faithfully recapitulated endogenous recruitment behavior.

Following small laser-induced wounds (∼1 μm), VPS-32 was rapidly recruited to the lesion, where it closely colocalized with the plasma membrane marker throughout the repair process (Fig. 3 A and Video 5). VPS-32 formed discrete membrane-associated puncta that reached maximal intensity within 30 min after wounding before gradually disappearing (Fig. 3, A and C). Thus, repair of small wounds is accompanied by a rapid and transient accumulation of VPS-32, which remains tightly associated with the damaged membrane, consistent with the formation of ESCRT-III spiral filaments that mediate constriction and scission of small membrane lesions (Jimenez et al., 2014).

**Video 5.** **Small laser wounding induced VPS-32 recruitment to the wounded membrane.** Time-lapse spinning-disk confocal microscopy of the epidermis of a young adult *C. elegans* carrying *Pcol-19-myr::mKate2(zjuSi46); Pvps-32-VPS-32::GFP(zjuSi443)*. A Micropoint UV laser was used to generate a small (2–3 μm) wound. Each frame shows the plasma membrane marker myr::mKate2 (red, top left), VPS-32::GFP (green, top right), and the merged image (bottom). Images were taken at 30-s intervals and are played back at 24 frames/s. Elapsed time after wounding (HH:mm:ss) is displayed in the lower-right corner. Scale bar, 10 μm. Related to Fig. 3 A.

In contrast, large plasma membrane wounds (∼10 μm) triggered markedly different VPS-32 dynamics. Shortly after injury, numerous transient VPS-32–positive puncta appeared throughout a broad peri-wound region extending up to ∼50 μm from the wound edge (Fig. 3, B and D; and Fig. S1, L and M). These puncta were highly dynamic, undergoing repeated appearance, disappearance, movement, and fusion before progressively accumulating at the wound perimeter (Video 6). Rather than remaining as isolated puncta, VPS-32 ultimately assembled into a prominent circumferential structure that surrounds the wound margin and persists throughout the repair process (Fig. 3 B and Video 6).

**Video 6.** **Large laser wounding induced VPS-32 recruitment to the wounded membrane.** Time-lapse spinning-disk confocal microscopy of the epidermis of a young adult *C. elegans* carrying *Pcol-19-myr::mKate2(zjuSi46); Pvps-32-VPS-32::GFP(zjuSi443)*. A Micropoint UV laser was used to generate a large (∼10 μm) wound. Magenta, plasma membrane marker myr::mKate2; green, VPS-32::GFP. Images were taken at 30-s intervals and are played back at 10 frames/s. Elapsed time after wounding (HH:mm:ss) is displayed in the lower-right corner. Scale bar, 10 μm. Related to Fig. 3 B.

Assembly of VPS-32 occurred concurrently with extensive membrane remodeling. The plasma membrane marker myr::mKate2 progressively accumulated around the wound periphery and exhibited strong spatial and temporal overlap with VPS-32 throughout the repair process (Fig. 3, E and F; and Video 7). Because myr::mKate2 labels the plasma membrane, its progressive enrichment likely reflects the membrane remodeling and membrane addition that accompany large plasma membrane repair (Wang et al., 2022; Meng et al., 2023). These observations indicate that VPS-32 assembly is closely coordinated with wound-associated membrane remodeling rather than representing an independent cytosolic aggregation event.

**Video 7.** **Needle wounding induced VPS-32 recruitment to the wounded membrane.** Time-lapse spinning-disk confocal microscopy of the epidermis of a young adult *C. elegans* carrying *Pcol-19-myr::mKate2(zjuSi46); Pvps-32-VPS-32::GFP(zjuSi443)*, wounded by manual puncture with a hand-pulled glass needle. Each frame shows the plasma membrane marker myr::mKate2 (red, top left), VPS-32::GFP (green, top right), and the merged image (bottom). Images were acquired at 1 min intervals and are played back at 9 frames/s. Elapsed time after wounding (HH:mm:ss) is displayed in the lower-right corner. Scale bar, 10 μm. Related to Fig. 3 E.

To determine whether the wound-associated VPS-32 assembly represents a stable structure or undergoes continuous turnover, we performed FRAP on both small-scale and large-scale wound-associated VPS-32 assemblies. In the unwounded membrane, both VPS-32::GFP and the myr::mKate2 recovered rapidly after photobleaching, consistent with free diffusion under basal conditions (Fig. S1, N and O; and Video 8). Following small wounds, VPS-32::GFP recovered rapidly and completely after photobleaching (t½ <2 min) (Fig. 3, G and H; and Video 9), indicating continuous exchange of VPS-32 molecules during repair. In contrast, VPS-32::GFP associated with large wounds recovered substantially more slowly (t½ ∼20 min) and remained only partially recovered throughout the imaging period (Fig. 3, G and H; and Video 10), demonstrating substantially reduced molecular turnover.

**Video 8.** **FRAP analysis of VPS-32::GFP in unwounded epidermis.** FRAP of VPS-32::GFP in the unwounded epidermis of a young adult *C. elegans* carrying *Pcol-19-myr::mKate2(zjuSi46); Pvps-32-VPS-32::GFP(zjuSi443)*. Each frame shows myr::mKate2 (red, top left), VPS-32::GFP (green, top right), and the merged image (bottom). A region of the unwounded epidermis was photobleached, and fluorescence recovery was imaged every 10 s for 20 min; the movie is played back at 12 frames/s. Elapsed time after wounding (HH:mm:ss) is displayed in the lower-right corner. Scale bar, 10 μm. Related to Fig. S1 N.

**Video 9.** **FRAP analysis of VPS-32::GFP at small laser-induced wounds.** FRAP of VPS-32::GFP at a small (∼1 μm) laser-induced wound in the epidermis of a young adult *C. elegans* carrying *Pcol-19-myr::mKate2(zjuSi46); Pvps-32-VPS-32::GFP(zjuSi443)*. Each frame shows myr::mKate2 (red, top left), VPS-32::GFP (green, top right), and the merged image (bottom). The wound-associated VPS-32::GFP aggregate was photobleached, and recovery was recorded at 10-s intervals for 20 min; the movie is played back at 12 frames/s. Elapsed time after wounding (HH:mm:ss) is displayed in the lower-right corner. Scale bar, 10 μm. Related to Fig. 3 G.

**Video 10.** **FRAP analysis of VPS-32::GFP at large wound sites.** FRAP of VPS-32::GFP at the wound-associated aggregate 1 h after large-scale wounding in the epidermis of a young adult *C. elegans* carrying *Pcol-19-myr::mKate2(zjuSi46); Pvps-32-VPS-32::GFP(zjuSi443)*. Each frame shows myr::mKate2 (red, top left), VPS-32::GFP (green, top right), and the merged image (bottom). Recovery was at 10-s intervals for 20 min; the movie is played back at 12 frames/s. Elapsed time after wounding (HH:mm:ss) is displayed in the lower-right corner. Scale bar, 10 μm. Related to Fig. 3 G.

Thus, although VPS-32 remains dynamic at large wounds, it exhibits markedly reduced turnover compared with small wounds, demonstrating that the wound-associated ESCRT-III assembly becomes substantially more persistent as wound size increases. Together, these results support a wound size–dependent model of ESCRT-III assembly: small membrane lesions recruit rapidly exchanging VPS-32 assemblies, whereas large wounds induce the formation of a long-lived circumferential ESCRT-III structure that remains associated with the membrane throughout much of the repair process.

### VPS-32 polymerization is required for the formation of wound-associated ESCRT-III structure and membrane repair

**Figure 4.**
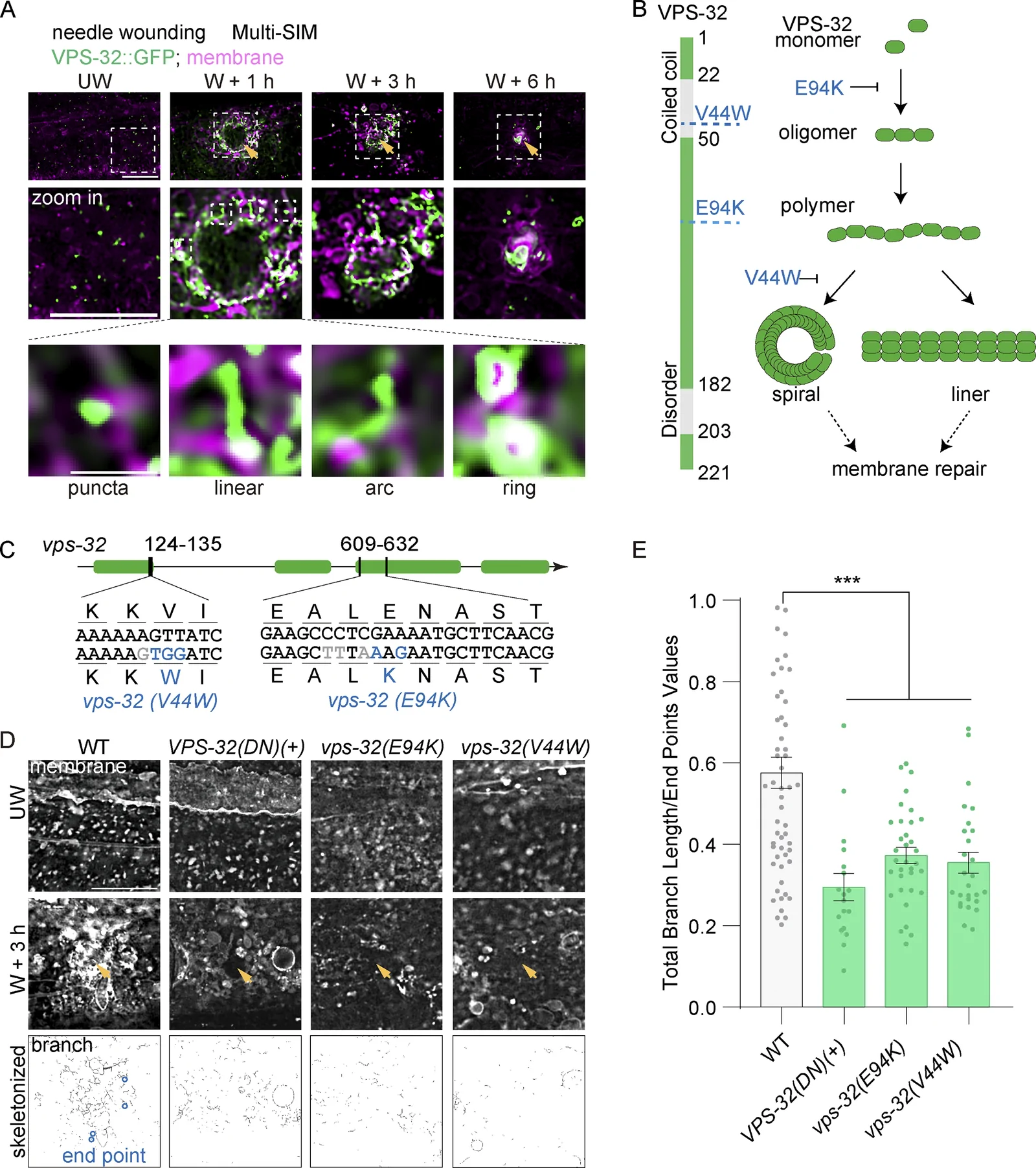
**VPS-32 polymerization is required for assembly of the wound-associated ESCRT-III structure. (A)** Representative multi-SIM super-resolution images of VPS-32::GFP and myr::mKate2 at the wound site at the indicated time points after needle-induced wounding in epidermal cells. *Pcol-19-myr::mKate2(zjuSi46); Pvps-32-VPS-32::GFP(zjuSi443)* transgenic animals were used. Arrows indicate wound sites. Scale bar, 10 μm; zoom-in scale bar, 1 μm. **(B)** Schematic of the VPS-32 protein structure indicating the positions of the *vps-32(V44W)* and *vps-32(E94K)* mutations that impair VPS-32 polymerization. **(C)** Schematic of the CRISPR-Cas9 strategy used to generate endogenous *vps-32(V44W)* and *vps-32(E94K)* alleles, including the corresponding nucleotide and amino acid substitutions. **(D)** Representative multi-SIM images showing membrane organization at wound sites 3 h after needle-induced wounding in WT, VPS-32(DN)–overexpressing, and *vps-32* mutant animals. *Pcol-19-myr::tagBFP(zjuSi58)* transgenic animals expressing *Pcol-19-mKate2::VPS-32(zjuEx3088)* or carrying *vps-32(V44W)(syb10631)* or *vps-32(E94K)(syb10582)* alleles were used. Membrane continuity, branching, and endpoints are highlighted. Arrows indicate wound sites. Scale bar,10 μm. **(E)** Quantification of membrane structural complexity, expressed as the ratio of total branch length to number of endpoints, in WT, VPS-32(DN)-overexpressing, and *vps-32* mutant animals. Data are presented as mean ± SEM (*n* = 56, 18, 34, and 27 from left to right). Statistical significance was assessed by the Kruskal–Wallis test, ***P < 0.001.

**Figure S2.**
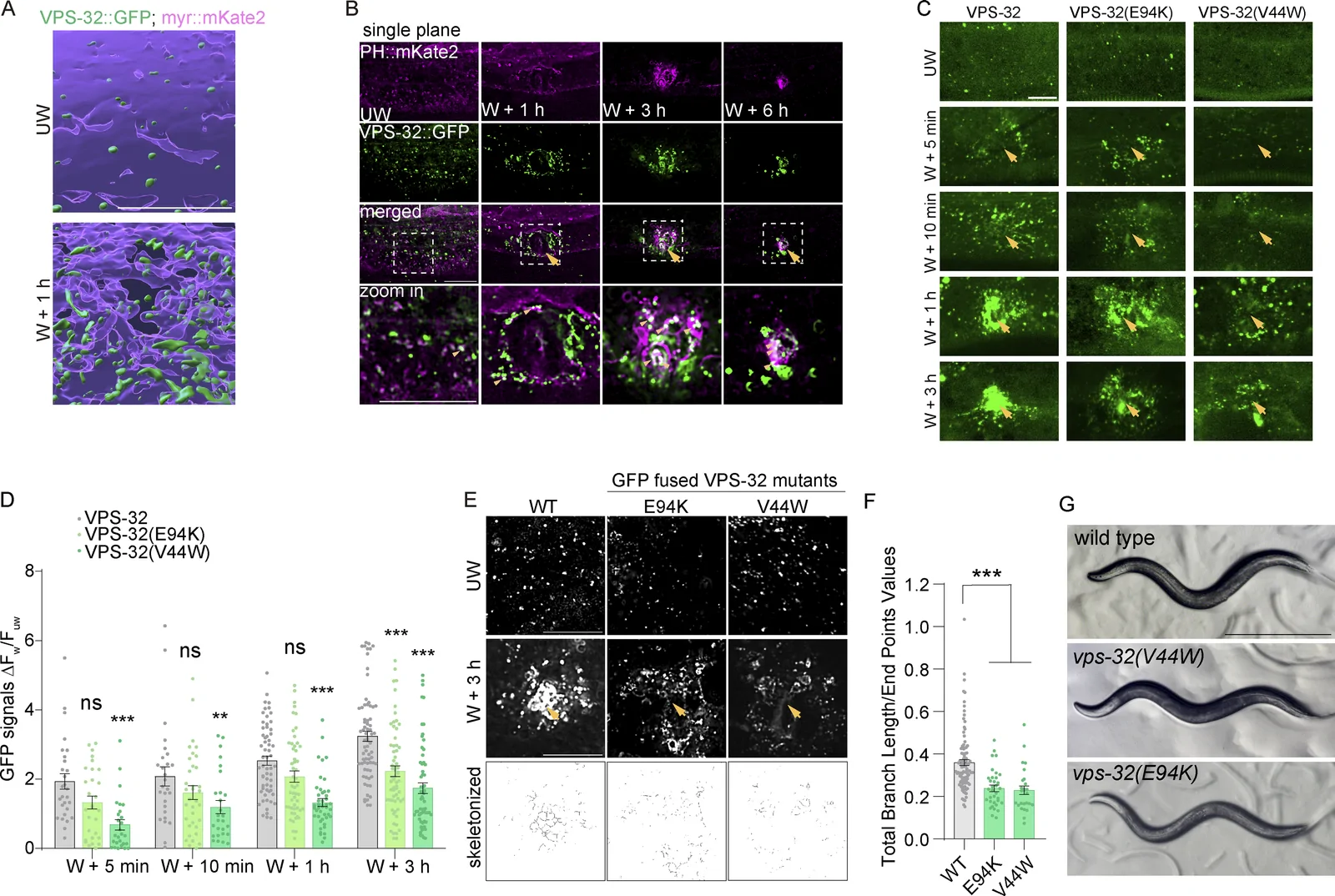
**VPS-32 polymerization is required for wound-associated ESCRT-III assembly. (A)** Multi-SIM images showing VPS-32::GFP and the plasma membrane marker myr::mKate2 in unwounded epidermis and 1 h after needle-induced wounding. 3D reconstructions are shown. *Pcol-19-myr::mKate2(zjuSi46); Pvps-32-VPS-32::GFP(zjuSi443)* transgenic animals were used. Scale bar, 10 μm. **(B)** Multi-SIM images showing VPS-32::GFP and the plasma membrane marker PH::mKate2 in single-plane views before and at the indicated times after needle-induced wounding. *Pcol-19-PH::mKate2(zjuSi327); Pvps-32-VPS-32::GFP(zjuSi429)* transgenic animals were used. Arrows indicate wound sites. Arrowheads indicate sites of colocalization. Scale bar, 10 μm. **(C)** Representative confocal images showing recruitment of WT VPS-32::GFP, VPS-32(E94K)::GFP, and VPS-32(V44W)::GFP to wound sites at the indicated times after needle-induced wounding. *Pvps-32-VPS-32::GFP(zjuSi585)*, *Pvps-32-VPS-32(E94K)::GFP(zjuSi571)*, and *Pvps-32-VPS-32(V44W)::GFP(zjuSi573)* transgenic animals were used. Arrows indicate wound sites. Scale bar, 10 μm. **(D)** Quantification of VPS-32::GFP fluorescence intensity at wound sites in WT, E94K, and V44W animals at the indicated times after needle-induced wounding. Data are presented as mean ± SEM (*n* = 28, 29, 27, 27, 35, 27, 61, 52, 45, 73, 64, and 63 from left to right). Statistical significance was assessed by the Kruskal–Wallis test, ***P < 0.001; **P < 0.01; ns, not significant. **(E)** Multi-SIM and corresponding skeletonized images of WT VPS-32::GFP, VPS-32(E94K)::GFP, and VPS-32(V44W)::GFP before and at 3 h after needle-induced wounding. *Pvps-32-VPS-32::GFP(zjuSi585)*, *Pvps-32-VPS-32(E94K)::GFP(zjuSi571)* and *Pvps-32-VPS-32(V44W)::GFP(zjuSi573)* transgenic animals were used. Arrows indicate wound sites. Scale bar, 10 μm. **(F)** Quantification of total branch length and branch endpoints ratio of skeletonized VPS-32 assemblies in WT VPS-32::GFP, VPS-32(E94K)::GFP, and VPS-32(V44W)::GFP animals. Data are presented as mean ± SEM (*n* = 100, 34, and 26 from left to right). Statistical significance was assessed by the Kruskal–Wallis test, ***P < 0.001. **(G)** Representative bright-field images of WT, *vps-32(V44W)*, and *vps-32(E94K)* animals at young adult stage (day 1). Scale bar, 500 μm.

ESCRT-III proteins are capable of assembling into a range of supramolecular structures, including linear filaments, spirals, and worm-like polymers, that are thought to drive their remodeling activity (Schmidt et al., 2020; Schöneberg et al., 2018; McCullough et al., 2015; Henne et al., 2013; Tang et al., 2015; McCullough et al., 2018; Liu et al., 2024). To examine the organization of VPS-32 at large plasma membrane wounds, we performed structured illumination microscopy (SIM) imaging on wounded epidermis. Super-resolution imaging revealed that VPS-32 formed extended membrane-associated assemblies composed of interconnected linear and spiral-like elements distributed around the wound perimeter (Fig. 4 A; and Fig. S2, A and B).

To test whether formation of this wound-associated ESCRT-III structure depends on VPS-32 polymerization, we analyzed two previously characterized VPS-32 variants that disrupt ESCRT-III filament assembly *in vitro* (Shen et al., 2014). The V44W mutation interferes with filament continuity, whereas E94K stabilizes the closed monomeric conformation of VPS-32, thereby preventing productive polymerization (Shen et al., 2014) (Fig. 4 B). We generated single-copy expression of GFP-tagged versions of each mutant and monitored their behavior following membrane injury. VPS-32(V44W)::GFP exhibited markedly reduced recruitment to wound sites, whereas VPS-32(E94K)::GFP was recruited with kinetics comparable with WT during early stages of repair and reduced 1 h after wounding (Fig. S2, C and D). Despite these differences in early recruitment, both proteins remained as dispersed puncta that failed to mature into higher-order assemblies 3 h after wounding, as revealed by super-resolution SIM imaging (Fig. S2, E and F). These observations demonstrate that initial wound-induced recruitment and subsequent polymerization are mechanistically separable processes and that VPS-32 polymerization is required for maturation of the persistent wound-associated ESCRT-III assembly.

To determine the functional importance of VPS-32 polymerization, we generated CRISPR-Cas9 animals carrying *vps-32(V44W)* and *vps-32(E94K)* mutations at the endogenous locus (Fig. 4 C). *vps-32(V44W)* mutant strains were viable and exhibited normal development under basal conditions, whereas *vps-32(E94K)* animals were relatively shorter and thinner than WT (Fig. S2 G). Super-resolution SIM imaging revealed pronounced defects in membrane remodeling at wound sites in *vps-32* mutants after wounding (Fig. 4, D and E). Whereas WT animals restored a continuous membrane architecture during repair, both polymerization-defective mutants displayed fragmented and discontinuous myr::mKate2 labeling around the wound (Fig. 4, D and E), consistent with defective membrane remodeling. Together, these data demonstrate that VPS-32 polymerization is dispensable for the initial recruitment of ESCRT-III to wounded membranes but is essential for assembly of the persistent wound-associated ESCRT-III structure and for efficient repair of large plasma membrane wounds.

### VPS-32 polymerization promotes vesicle recruitment during large plasma membrane repair

**Figure S3.**
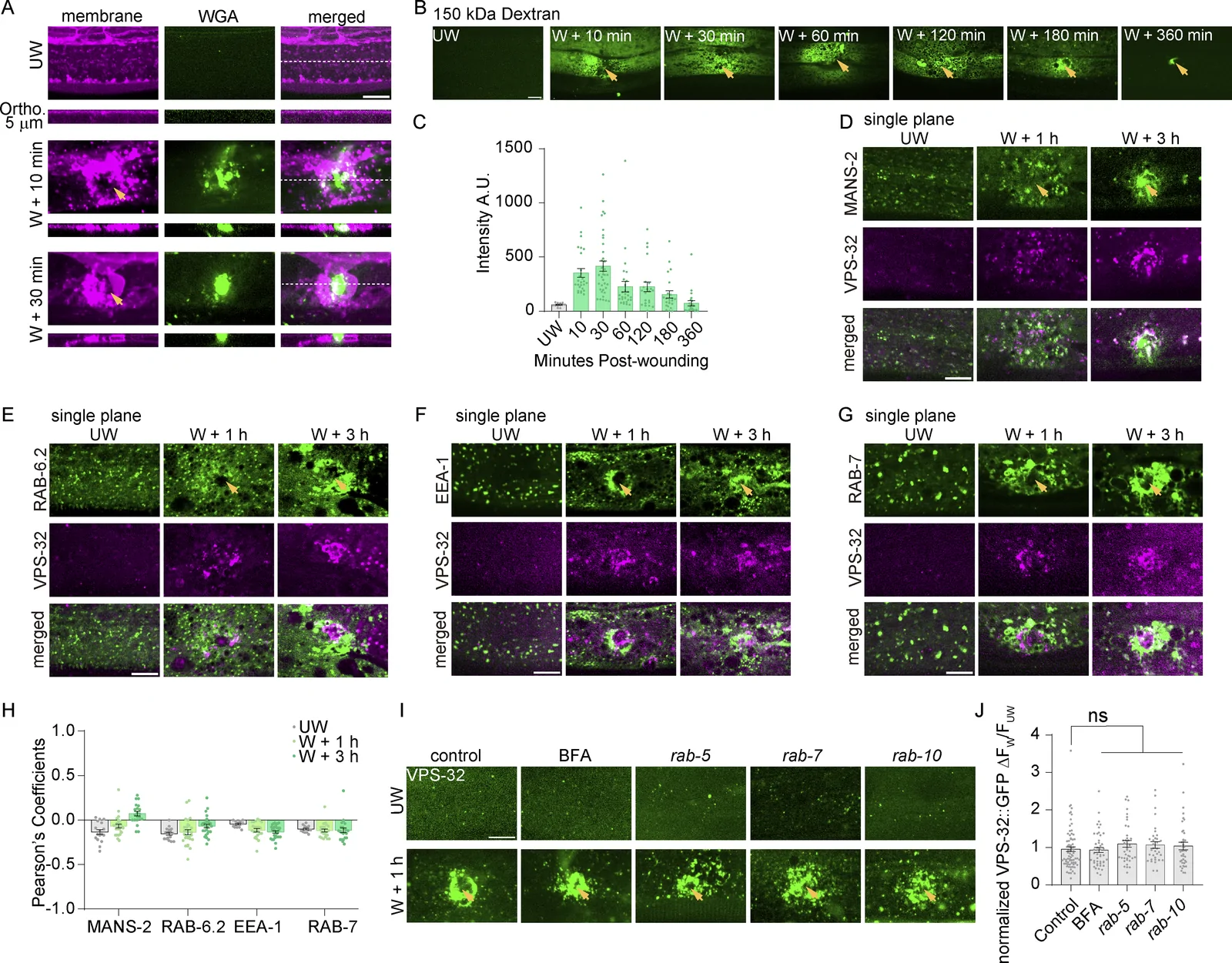
**VPS-32 assembly precedes and is independent of vesicle recruitment. (A)** Representative confocal images of the plasma membrane marker myr::mKate2 (magenta) and WGA staining (green) in unwounded and wounded epidermis at the indicated times after needle-induced wounding. Orthogonal views and merged images show accumulation of WGA-positive material at the wound margin. *Pcol-19-myr::mKate2(zjuSi46)* transgenic animals were used. Arrows indicate wound sites. Scale bar, 10 μm. **(B)** Representative confocal images of 150-kDa fluorescent dextran permeability assays in unwounded and wounded epidermal cells at the indicated times after needle-induced wounding. Arrows indicate wound sites. Scale bar, 10 μm. **(C)** Quantification of 150-kDa dextran fluorescence intensity measured 30 μm from the wound edge at the indicated times after needle-induced wounding. Data are presented as mean ± SEM (*n* = 12, 27, 38, 28, 22, 23, and 22 from left to right). **(D–G)** Representative single-plane confocal images showing colocalization of the vesicle markers GFP::MANS-2 (D), GFP::RAB-6.2 (E), GFP::EEA-1 (F), and mKate2::RAB-7 (G) with VPS-32::tagBFP (magenta) in unwounded and wounded epidermis at the indicated times after needle-induced wounding. Animals expressing *Pvps-32-VPS-32::tagBFP(zjuSi457)* together with the indicated vesicle marker were used. Arrows indicate wound sites. Scale bar, 10 μm. **(H)** Pearson’s correlation coefficients quantifying colocalization between VPS-32::tagBFP and the indicated vesicle markers at wound sites. Data are presented as mean ± SEM (*n* = 17, 27, 20, 16, 28, 27, 14, 23, 32, 15, 23, and 23 from left to right). **(I)** Representative confocal images of VPS-32::GFP recruitment to wound sites in control, Brefeldin A (BFA)-treated, and *rab-5*, *rab-7*, and *rab-10* RNAi–treated animals (from L4 stage) at unwounded and 1 h after needle-induced wounding. *Pvps-32-VPS-32::GFP(zjuSi429)* transgenic animals were used. Arrows indicate wound sites. Scale bar, 10 μm. **(J)** Quantification of normalized VPS-32::GFP fluorescence intensity at wound sites under the indicated conditions. Data are presented as mean ± SEM (*n* = 76, 46, 39, 35, and 40 from left to right). Statistical significance was assessed by the Kruskal–Wallis test; ns, not significant.

Large plasma membrane wounds remain morphologically visible for up to 60 min after injury without causing immediate cell death, suggesting that the wound is progressively occluded during repair rather than remaining as an open membrane rupture. To examine whether extracellular material accumulates at the wound site during this process, we stained wounded animals with wheat germ agglutinin (WGA), which labels glycocalyx-associated carbohydrates (Natsuka et al., 2005). Little or no WGA staining was detected on the apical surface of unwounded epidermal cells (Fig. S3 A). In contrast, following needle wounding, WGA-positive material progressively accumulated around the wound margin (Fig. S3 A), indicating the presence of carbohydrate-rich material at the repair site. Although the molecular identity of this material remains unknown, its enrichment is consistent with progressive wound occlusion during membrane repair.

To further assess whether the wound site has regained barrier function, we performed fluorescent dextran permeability assays with 150-kDa dextran. Dextran readily entered wounded cells immediately after injury and remained detectable if we stained the wounded animals at the early stages of repair (10 min–3 h) (Fig. S3, B and C), but intracellular fluorescence progressively decreased when we stained the wounded animals over time. By 6 h after wounding, dextran was rarely stained in wounded cells (Fig. S3, B and C), indicating that membrane barrier function had been substantially restored. These observations demonstrate that repair of large plasma membrane wounds is a gradual process *in vivo*, during which permeability progressively decreases rather than being restored immediately.

Our previous studies demonstrated that plasma membrane injury induces rapid recruitment of the Golgi-derived vesicles to the wound site, where SNARE-mediated membrane fusion contributes to the formation of a membrane patch during repair (Meng et al., 2023; Shao et al., 2023). We therefore asked whether wound-associated ESCRT-III assembly is linked to these membrane trafficking events. Consistent with previous observations, the Golgi markers MANS-2 and RAB-6.2, the early endosome marker EEA-1, and the late endosome marker RAB-7 were all robustly recruited to wound sites following injury (Fig. S3, D–H), confirming extensive activation of intracellular membrane trafficking during repair. However, none of these markers showed substantial colocalization with VPS-32 (Fig. S3, D–H), indicating that the wound-associated ESCRT-III assembly is largely distinct from the vesicle populations that accumulate during repair.

**Figure 5.**
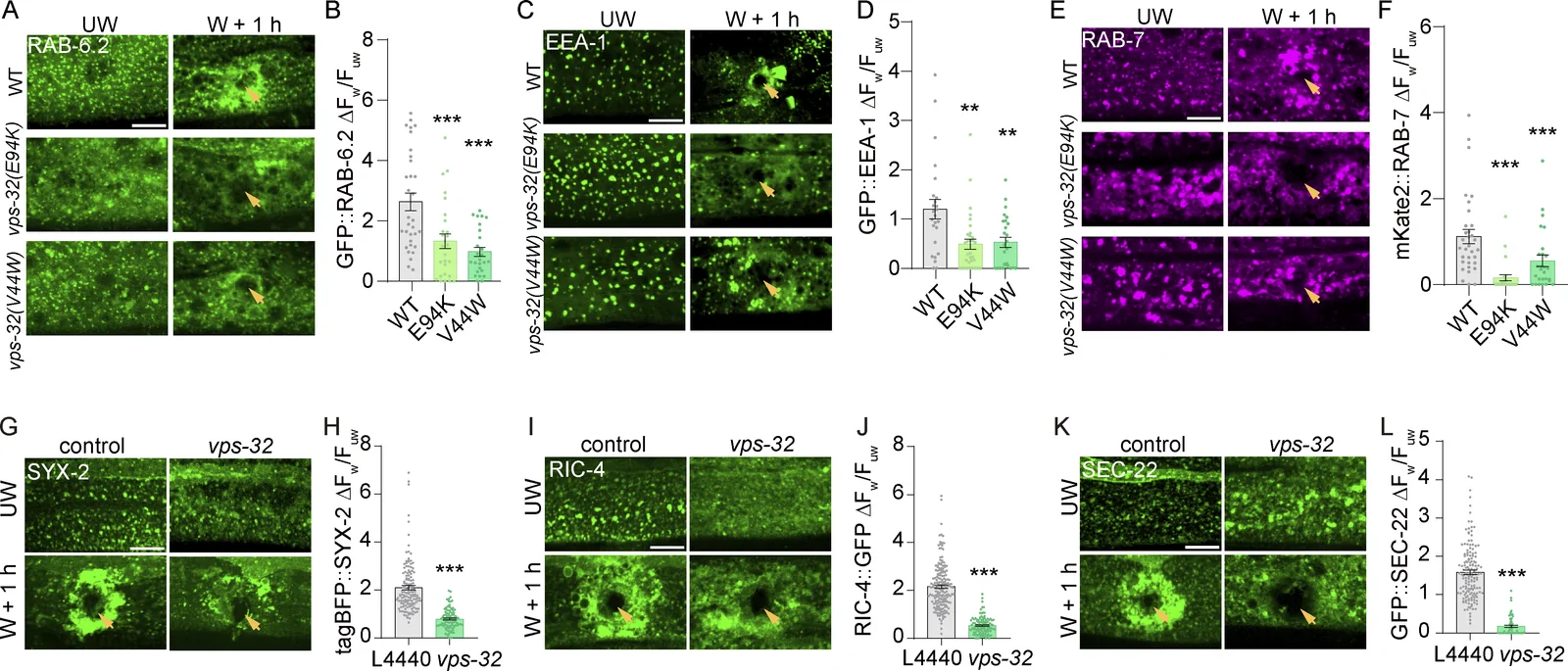
**VPS-32 polymerization promotes recruitment of intracellular membrane compartments and fusion machinery during plasma membrane repair. (A)** Representative confocal images showing GFP::RAB-6.2 localization in WT and *vps-32* mutants before and 1 h after needle-induced wounding. *Pcol-19-GFP::RAB-6.2(zjuSi313)* transgenic animals carrying *vps-32(V44W)(syb10631)* or *vps-32(E94K)(syb10582)* alleles were used. Arrows indicate wound sites. Scale bar, 10 μm. **(B)** Quantification of GFP::RAB-6.2 fluorescence intensity at wound sites in WT and *vps-32* mutants. Data are presented as mean ± SEM (*n* = 32, 27, and 26 from left to right). Statistical significance was assessed by the Kruskal–Wallis test, ***P < 0.001. **(C)** Representative confocal images showing GFP::EEA-1 localization in WT and *vps-32* mutants before and 1 h after needle-induced wounding. *Pcol-19-GFP::EEA-1(zjuSi303)* transgenic animals carrying *vps-32(V44W)(syb10631)* or *vps-32(E94K)(syb10582)* alleles were used. Arrows indicate wound sites. Scale bar, 10 μm. **(D)** Quantification of GFP::EEA-1 fluorescence intensity at wound sites in WT and *vps-32* mutants. Data are presented as mean ± SEM (*n* = 25, 32, and 26 from left to right). Statistical significance was assessed by the Kruskal–Wallis test, **P < 0.01. **(E)** Representative confocal images showing mKate2::RAB-7 localization in WT and *vps-32* mutants before and 1 h after needle-induced wounding. *Pcol-19-mKate2::RAB-7(zjuSi188)* transgenic animals carrying *vps-32(V44W)(syb10631)* or *vps-32(E94K)(syb10582)* alleles were used. Arrows indicate wound sites. Scale bar, 10 μm. **(F)** Quantification of mKate2::RAB-7 fluorescence intensity at wound sites in WT and *vps-32* mutants. Data are presented as mean ± SEM (*n* = 33, 30, and 27 from left to right). Statistical significance was assessed by the Kruskal–Wallis test, ***P < 0.001. **(G)** Representative confocal images showing tagBFP::SYX-2 in *vps-32* RNAi-treated animals before and 1 h after needle-induced wounding. *Pcol-19-tagBFP::SYX-2(zjuSi72)* transgenic animals were used. Arrows indicate wound sites. Scale bar, 10 μm. **(H)** Quantification of tagBFP::SYX-2 fluorescence intensity at wound sites in control and *vps-32* RNAi-treated animals. Data are presented as mean ± SEM (*n* = 138 and 100, respectively). Statistical significance was assessed by the Mann–Whitney test, ***P < 0.001. **(I)** Representative confocal images showing RIC-4::GFP in control and *vps-32* RNAi-treated animals before and 1 h after needle-induced wounding. *Pcol-19-RIC-4::GFP(zjuSi309)* transgenic animals were used. Arrows indicate wound sites. Scale bar, 10 μm. **(J)** Quantification of RIC-4::GFP fluorescence intensity at wound sites in control and *vps-32* RNAi-treated animals. Data are presented as mean ± SEM (*n* = 187 and 117, respectively). Statistical significance was assessed by the Mann–Whitney test, ***P < 0.001. **(K)** Representative confocal images showing GFP::SEC-22 in control and *vps-32* RNAi-treated animals before and 1 h after needle-induced wounding. *Pcol-19-GFP::SEC-22(zjuSi292)* transgenic animals were used. Arrows indicate wound sites. Scale bar, 10 μm. **(L)** Quantification of GFP::SEC-22 fluorescence intensity at wound sites in control and *vps-32* RNAi-treated animals. Data are presented as mean ± SEM (*n* = 152 and 64, respectively). Statistical significance was assessed by the Mann–Whitney test, ***P < 0.001.

We next asked whether VPS-32 polymerization contributes to vesicle recruitment. Notably, wound-induced accumulation of RAB-6.2–positive Golgi vesicles, EEA-1–positive early endosomes, and RAB-7–positive late endosomes was significantly reduced in both *vps-32(V44W)* and *vps-32(E94K)* mutants (Fig. 5, A–F). Similarly, recruitment of the membrane fusion machinery, including the t-SNARE SYX-2, SNAP-25 homolog RIC-4, and v-SNARE SEC-22, was markedly diminished in *vps-32* RNAi-treated animals (Fig. 5, G–L). Conversely, epidermal-specific depletion of *rab-5*, *rab-7*, or *rab-10*, as well as disruption of Golgi-dependent trafficking with Brefeldin A, had little effect on VPS-32 accumulation at wound sites (Fig. S3, I and J). Together, these findings suggest that VPS-32 structure polymerization is required for the efficient recruitment of intracellular membrane compartments and membrane fusion machinery needed for progressive wound occlusion and repair.

### ESCRT-III is required to restore membrane tension during repair

**Figure 6.**
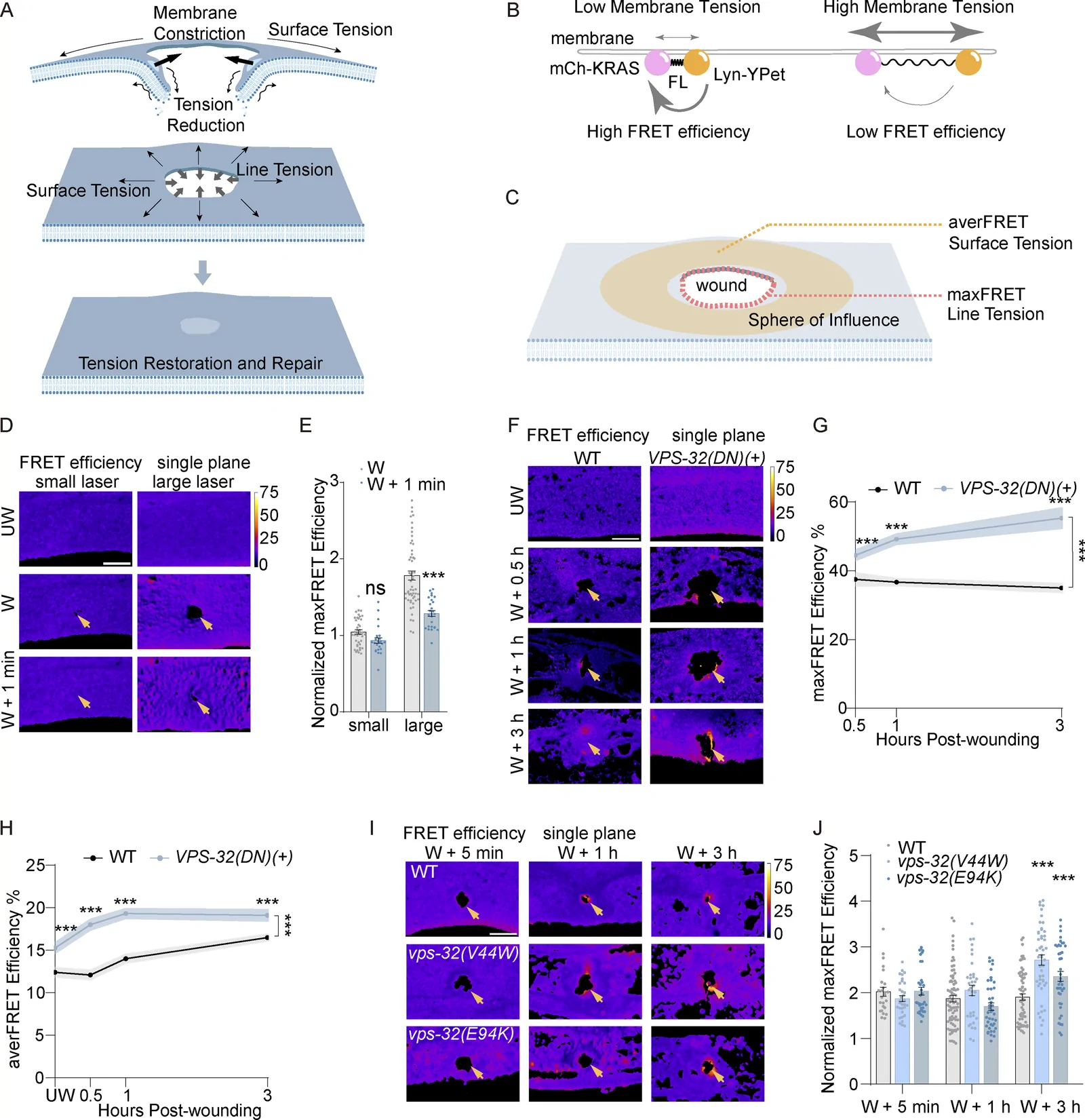
**VPS-32 polymerization is required for restoration of membrane tension during large-scale wound repair. (A)** Schematic illustrating the proposed mechanical changes during large-scale plasma membrane repair, including acute loss of surface tension, disruption of mechanical integrity at the wound edge, and subsequent restoration of line tension during membrane repair. **(B)** Schematic of the plasma membrane tension sensor module (TSM), consisting of two membrane-anchoring modules connected by a FRET-based tension-sensing linker. **(C)** Schematic illustrating the regions used for quantifying maxFRET at the wound edge and averFRET in the surrounding membrane region. **(D)** Representative single-plane confocal images showing FRET efficiency of the TSM at small (∼1 μm) and large (∼10 μm) laser-induced wounds in WT animals before and 1 min after wounding. *Pcol-19-TSM* tension sensor*(zjuSi229)* transgenic animals were used. FRET efficiency was normalized to the corresponding unwounded region. The color scale indicates FRET efficiency (%). Arrows indicate wound sites. Scale bar, 10 μm. **(E)** Quantification of normalized maxFRET efficiency at small and large wounds before and 1 min after wounding. Data are presented as mean ± SEM (*n* = 41, 26, 55, and 26 from left to right). Statistical significance was assessed by the Mann–Whitney test; ***P < 0.001; ns, not significant. **(F)** Representative single-plane confocal images showing TSM FRET efficiency in WT and VPS-32(DN)-overexpressing animals at the indicated time points after large needle-induced wounding. *Pcol-19-TSM(zjuSi229); Pcol-19-mKate2::VPS-32(zjuEx3095)* transgenic animals were used. Arrows indicate wound sites. Scale bar, 10 μm. **(G)** Quantification of maxFRET analysis at wound edges over time following large needle-induced wounding in WT (*n* = 31, 48, and 56, respectively) and VPS-32(DN)-overexpressing animals (*n* = 48, 55, and 28, respectively). Data are presented as mean ± SEM. Statistical significance was assessed by the Mann–Whitney test, ***P < 0.001. **(H)** Quantification of averFRET analysis within the surrounding membrane region over time following large needle-induced wounding in WT (*n* = 38, 31, 48, and 56, respectively) and VPS-32(DN)-overexpressing animals (*n* = 28, 48, 55, and 28, respectively). Data are presented as mean ± SEM. Statistical significance was assessed by the Mann–Whitney test, ***P < 0.001. **(I)** Representative single-plane confocal images showing FRET efficiency in WT, *vps-32(V44W)*, and *vps-32(E94K)* animals at the indicated time points after needle-induced wounding. *Pcol-19-TSM(zjuSi229)* transgenic animals carrying *vps-32(V44W)(syb10631)* or *vps-32(E94K)(syb10582)* alleles were used. The color scale indicates FRET efficiency (%). Arrows indicate wound sites. Scale bar, 10 μm. **(J)** Quantification of normalized maxFRET efficiency at needle-induced wound edges in WT and *vps-32* mutants at the indicated time points after needle-induced wounding. Data are presented as mean ± SEM (*n* = 25, 33, 35, 79, 35, 40, 68, 49, and 41 from left to right). Statistical significance was assessed by the Kruskal–Wallis test, ***P < 0.001.

Large plasma membrane ruptures lead to an acute loss of membrane tension, and subsequent tension restoration is a critical checkpoint for successful repair (Cooper and McNeil, 2015; McNeil and Kirchhausen, 2005; Raj et al., 2024). During the repair process, the line tension of the wound perimeter must be re-established to counteract the surface tension of the surrounding membrane (Kozlov and Chernomordik, 2015; Fig. 6 A). Despite its fundamental importance, how membrane tension is restored after large membrane injury remains poorly understood. Given that ESCRT-III polymers are both sensitive to membrane tension changes (McCullough et al., 2018; Liu et al., 2024; Pfitzner et al., 2020) and capable of membrane remodeling (Vietri et al., 2020; Burigotto and Carlton, 2026), we hypothesized that VPS-32 polymerization contributes to the recovery of membrane mechanics during large-wound repair.

**Figure S4.**
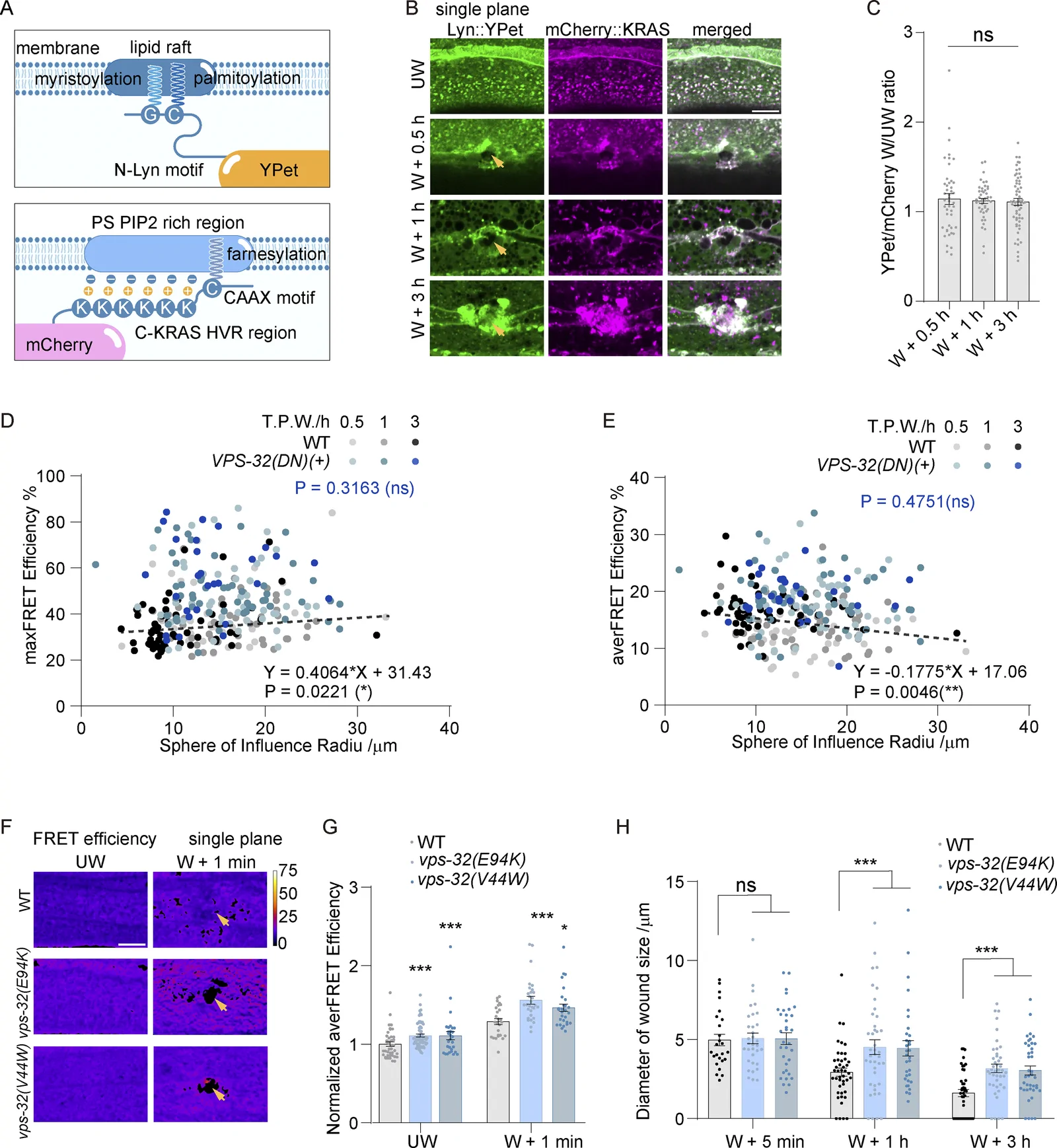
**VPS-32 polymerization is required for restoration of membrane tension after large-scale wounding. (A)** Schematic of the two membrane-targeting motifs used in the tension sensor control experiments. The N-Lyn motif (myristoylation and palmitoylation) targets YPet to lipid rafts, while the C-KRAS HVR (farnesylation) targets mCherry to PS/PIP2-rich membrane regions. **(B)** Representative confocal images of separately expressed N-Lyn::YPet (green) and mCherry::KRAS (magenta) in unwounded and wounded epidermis at the indicated times after needle-induced wounding. *Pcol-19-Lyn::YPet, Pcol-19-mCherry::KRAS(zjuEx3787)* transgenic animals were used. Arrows indicate wound sites. Scale bar, 10 μm. **(C)** Quantification of YPet/mCherry fluorescence intensity ratio (wounded/unwounded) at the indicated times after needle-induced wounding. Data are presented as mean ± SEM (*n* = 45, 46, and 60 from left to right). Statistical significance was assessed by the Kruskal–Wallis test; ns, not significant. **(D and E)** Scatter plots showing the relationship between sphere-of-influence radius and FRET efficiency in WT (*n* = 31, 48, and 56) and VPS-32(DN) (*n* = 48, 55, and 28) overexpressing animals at the indicated time points after wounding, related to Fig. 6, G and H. Maximal edge FRET efficiency (D); average peripheral FRET efficiency (E). Trend lines and *P* values are indicated; *P < 0.05, **P < 0.01. **(F)** Representative single-plane confocal images showing FRET efficiency in WT, *vps-32(E94K)*, and *vps-32(V44W)* animals before and 1 min after laser-induced wounding. *Pcol-19-TSM(zjuSi229)* with *vps-32(E94K)(syb10582)* or *vps-32(V44W)(syb10631)* animals were used. The color scale indicates FRET efficiency (%). Arrows indicate wound sites. Scale bar, 10 μm. **(G)** Quantification of normalized averFRET efficiency in WT and *vps-32* mutant animals before and 1 min after laser-induced wounding. Data are presented as mean ± SEM (*n* = 41, 67, 29, 26, 30, and 28 from left to right). Statistical significance was assessed by the Kruskal–Wallis test, ***P < 0.001; ns, not significant. **(H)** Quantification of wound size diameters in WT and *vps-32* mutants at 5 min, 1 h, and 3 h after needle-induced wounding, related to Fig. 6 I. Data are presented as mean ± SEM (*n* = 25, 34, 33, 44, 41, 32, 25, 42, and 40 from left to right). Statistical significance was assessed by the Kruskal–Wallis test, ***P < 0.001; ns, not significant. TSM, tension sensor module.

To quantify membrane mechanics *in vivo*, we employed a fluorescence resonance energy transfer (FRET)-based tension sensor module (Ringer et al., 2017) tethered to the plasma membrane (Fig. 6 B). The sensor consists of YPet and mCherry fluorophores linked by a spring-like ferredoxin-like peptide and anchored to the inner leaflet of the plasma membrane through N-terminal Lyn and C-terminal KRAS membrane-targeting motifs (Fig. S4 A) (Ringer et al., 2017). Stretching of the plasma membrane increases the distance between the fluorophores, resulting in reduced FRET efficiency, whereas membrane relaxation increases FRET.

Because plasma membrane injury is accompanied by extensive lipid remodeling, we first assessed whether wound-induced redistribution of membrane lipids might influence the FRET signal independently of membrane tension. To address this possibility, N-Lyn::YPet and mCherry::KRAS were expressed separately as unfused controls. The relative fluorescence intensity of the two membrane anchors remained unchanged at the wound edge throughout repair (Fig. S4, B and C), indicating that wound-induced lipid remodeling does not differentially redistribute the anchors. These results validate that changes in the intact sensor primarily report alterations in membrane mechanics rather than changes in membrane composition.

Using the intact tension sensor, we quantified both the maximum FRET (maxFRET)signal at the wound edge, which reflects local line tension, and the average FRET (averFRET) signal within the surrounding membrane, which reports the mechanical state of the adjacent plasma membrane (Fig. 6, B and C). Small laser-induced wounds (∼1 μm) produced no detectable changes in maxFRET throughout the repair process (Fig. 6, D and E), indicating that these lesions do not generate a measurable membrane tension deficit. In contrast, large wounds elicited a rapid increase in maxFRET within minutes after injury, consistent with a substantial reduction in membrane tension at the wound edge (Fig. 6, D and E). MaxFRET then progressively declined over the subsequent 3 h, indicating gradual recovery of local membrane tension (Fig. 6, F and G). Changes in averFRET followed a similar temporal pattern and reflected mechanical changes extending beyond the wound edge into the surrounding membrane (Fig. 6, F and H), suggesting coordinated restoration of membrane mechanics across the entire peri-wound region.

We next asked whether VPS-32 contributes to the restoration of membrane mechanics after injury. Animals expressing dominant-negative (DN) VPS-32 (Wang et al., 2022) displayed elevated basal averFRET under unwounded conditions (Fig. 6, F and H), indicating reduced membrane tension at baseline relative to WT animals. Following wounding, VPS-32(DN) animals failed to restore membrane tension: maxFRET remained persistently elevated at the wound edge, the normal spatial FRET gradient surrounding the wound was lost, and recovery of averFRET was severely impaired (Fig. 6, F–H; and Fig. S4, D and E). Consequently, the coordinated relationship between local wound-edge tension, surrounding membrane mechanics, and wound size observed in WT animals was substantially disrupted in VPS-32(DN) expressing animals (Fig. 6, F and H). These findings suggest that VPS-32 is required for the coordinated restoration of membrane mechanics during large-wound repair.

To determine whether this function depends specifically on VPS-32 polymerization, we next analyzed the polymerization-defective *vps-32(V44W)* and *vps-32(E94K)* endogenous mutants. Similar to VPS-32(DN) expressing animals, both mutants displayed elevated basal averFRET before injury, consistent with reduced membrane tension under resting conditions (Fig. S4, F and G). At 1 min after wounding, both mutants also exhibited significantly higher averFRET than WT (Fig. S4, F and G), indicating a greater acute reduction in membrane tension following injury. However, unlike WT animals, both mutants retained significantly elevated maxFRET at the wound edge even 3 h after injury, whereas WT animals showed substantial recovery (Fig. 6, I and J). Importantly, initial wound sizes were similar at 5 min after needle wounding and comparable between WT and mutants (Fig. S4 H). Together, these findings indicate that VPS-32 polymerization is essential for maintaining basal membrane tension and re-establishing membrane mechanics after large plasma membrane rupture.

### VPS-32 polymerization is required for membrane repair and axon regeneration

**Figure 7.**
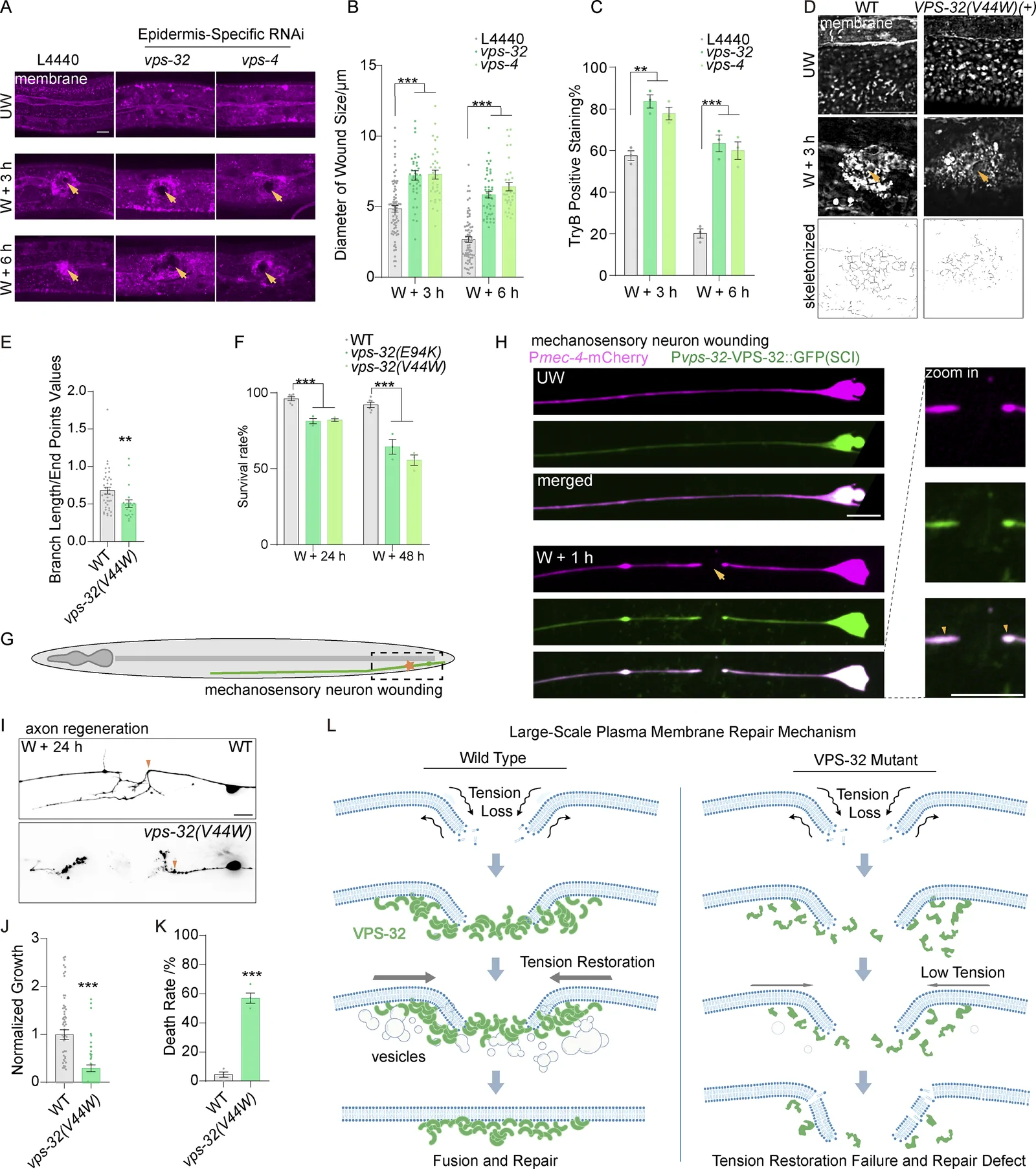
**VPS-32 polymerization is required for efficient membrane repair and axon regeneration. (A)** Representative confocal images showing membrane wound closure in animals subjected to epidermal-specific *vps-32* or *vps-4* RNAi at the indicated times after needle-induced wounding. Plasma membrane is labeled with myr::mKate2. *rde-1(ne219); Pcol-19-myr::mKate2(zjuSi46); Pcol-19-RDE-1(juIs346)* transgenic animals were used. Arrows indicate wound sites. Scale bar, 10 μm. **(B)** Quantification of wound diameter at 3 and 6 h after needle-induced wounding in epidermal-specific *vps-32* or *vps-4* RNAi animals. Data are presented as mean ± SEM (*n* = 76, 40, 35, 72, 48, and 34 from left to right). Statistical significance was assessed by the Kruskal–Wallis test, ***P < 0.001. **(C)** Quantification of TryB-positive wounds at 3 and 6 h after needle wounding in animals subjected to epidermal-specific *vps-32* or *vps-4* RNAi. Data are presented as mean ± SEM (*n* = 3 independent groups; at least 100 wound sites scored per group). Each RNAi group was compared with the L4440 control using Pearson’s χ-square test (Fisher’s exact test where expected counts were <5), with Bonferroni correction for multiple comparisons, **P < 0.01, ***P < 0.001. **(D)** Representative Multi-SIM images showing membrane organization and skeletonized structures at wound sites in wild-type and VPS-32(V44W)-overexpressing animals before and 3 h after needle-induced wounding. The plasma membrane was labeled with myr::tagBFP. *Pcol-19-myr::tagBFP(zjuSi58); Pvps-32-VPS-32(V44W)::GFP(zjuEx3206)* transgenic animals were used. Arrows indicate wound sites. Scale bar, 10 μm. **(E)** Quantification of membrane structural complexity, expressed as the ratio of total branch length to number of endpoints, in WT and VPS-32(V44W)-overexpressing animals. Data are presented as mean ± SEM (*n* = 39 and 20, respectively). Statistical significance was assessed by the Mann–Whitney test, **P < 0.01. **(F)** Quantification of survival 24 and 48 h after needle-induced wounding in WT and vps-32 mutant animals. Survival is normalized to the corresponding unwounded control groups. Data are presented as mean ± SEM (*n* = 6, 3, 3, 6, 3, and 3 independent groups from left to right; >50 animals scored per group). Each mutant was compared with the WT using Pearson’s χ-square test (Fisher’s exact test where expected counts were <5), with Bonferroni correction for multiple comparisons, ***P < 0.001. **(G)** Schematic illustrating laser axotomy of the PLM mechanosensory neuron. **(H)** Representative confocal images showing VPS-32::GFP at the PLM axon injury site before and 1 h after laser axotomy, magnified views are shown. The neuron was labeled with P*mec-4*-mCherry. *Pvps-32-VPS-32::GFP(zjuSi585); Pmec-4-mCherry(zjuEx3522)* transgenic animals were used. Arrows indicate wound sites. Arrowheads indicate colocalization of VPS-32::GFP with the injured neuron at the injury sites. Scale bar, 10 μm. **(I)** Representative confocal images showing PLM mechanosensory neurons in WT and *vps-32(V44W)* mutant animals 24 h after laser axotomy. Neurons are labeled with *Pmec-4-GFP. vps-32(V44W)*(*syb10631*)*;* P*mec-4-GFP*(*zdIs5*) animals were used. Arrowheads indicate axotomy sites. Scale bar, 10 μm. **(J)** Quantification of PLM axon regrowth length 24 h after laser-induced PLM axotomy in WT and *vps-32(V44W)* mutant animals. Data are presented as mean ± SEM (*n* = 62 and 46, respectively). Statistical significance was assessed by the Mann–Whitney test ***P < 0.001. **(K)** Quantification of mortality 24 h after laser-induced PLM axotomy in WT and *vps-32*(V44W) mutant animals. Data are presented as mean ± SEM (*n* = 4 independent groups; >20 animals scored per group). Genotypes were compared using Pearson’s χ-square test (Fisher’s exact test where expected counts were <5), ***P < 0.001. **(L)** Model illustrating how the wound-associated ESCRT-III structures promote membrane tension restoration and facilitate large-scale plasma membrane repair. Left, WT: Large-scale plasma membrane injury induces a local decrease in membrane tension. ESCRT components (green) are recruited to the wound periphery, where they assemble into a circumferential structure. This wound-associated ESCRT-III assembly enables restoration of membrane tension and facilitates recruitment of intracellular vesicles (transparent bubbles) to the wound site. Subsequent vesicle fusion with the plasma membrane contributes to membrane repair. Right, ESCRT-III polymerization mutant: ESCRT-III components are recruited to the wound site, but formation of a circumferential ESCRT-III structure is impaired. Consequently, membrane tension fails to recover efficiently, accompanied by reduced vesicle recruitment and fusion, resulting in defects in membrane repair.

**Figure S5.**
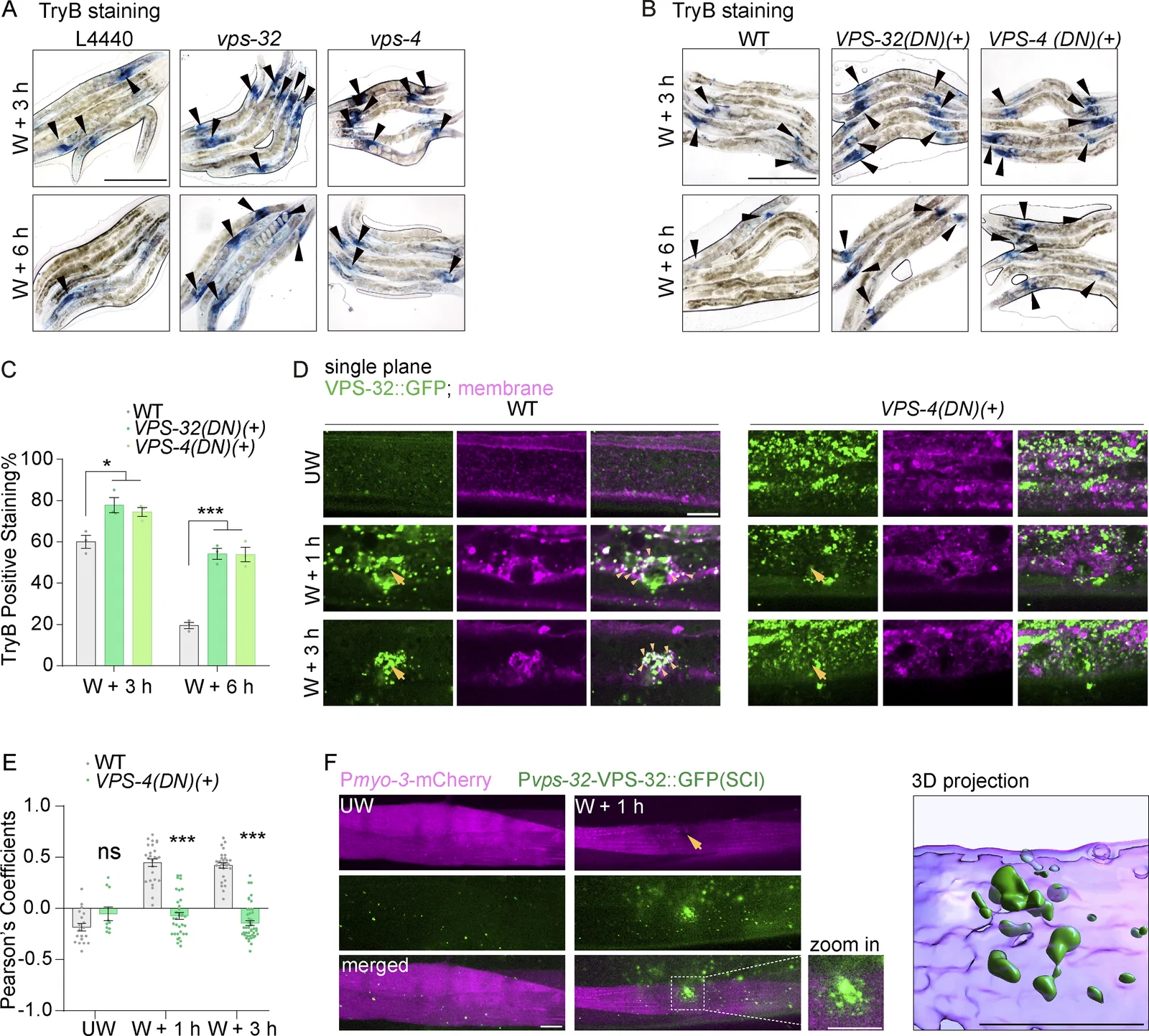
**VPS-32 polymerization is required for membrane repair across tissues. (A)** Representative TryB staining images of control (L4440), *vps-32*, and *vps-4* RNAi animals at 3 and 6 h after needle-induced wounding. Arrowheads indicate wound sites. Scale bar, 500 μm. **(B)** Representative TryB staining images of WT, VPS-32(DN)-overexpressing, and VPS-4(DN)-overexpressing animals at 3 and 6 h after needle-induced wounding. *Pcol-19-VPS-4(DN)(zjuEx2006)* and *Pcol-19-mKate2::VPS-32(zjuEx2734)* transgenic animals were used. Arrowheads indicate wound sites. Scale bar, 500 μm. **(C)** Quantification of TryB-positive staining at 3 and 6 h after needle-induced wounding in WT, VPS-32(DN)-overexpressing, and VPS-4(DN)-overexpressing animals. Data are presented as mean ± SEM (*n* = 3 independent groups; at least 100 wound sites scored per group). Each transgenic line was compared with the WT using Pearson’s χ-square test (Fisher’s exact test where expected counts were <5), with Bonferroni correction for multiple comparisons;*P < 0.05, ***P < 0.001. **(D)** Single-plane confocal images showing colocalization of VPS-32::GFP and myr::mKate2 at wound sites in WT and VPS-4(DN)-overexpressing worms 1 and 3 h after needle-induced wounding. *Pcol-19-myr::mKate2(zjuSi46); Pvps-32-VPS-32::GFP(zjuSi443); Pcol-19-VPS-4(DN)(zjuEx3046)* transgenic animals were used. Arrows indicate wound sites. Arrowheads indicate sites of colocalization. Scale bar, 10 μm. **(E)** Quantification of Pearson’s colocalization coefficients for VPS-32::GFP and myr::mKate2 at wound sites in WT and VPS-4(DN)-overexpressing animals at 1 and 3 h after needle-induced wounding. Data are presented as mean ± SEM (*n* = 19, 10, 26, 35, 29, and 45 from left to right). Statistical significance was assessed by the Mann–Whitney test; ***P < 0.001; ns, not significant. **(F)** Confocal images and three-dimensional reconstructions showing VPS-32::GFP and body wall muscle cytosol labelled animal before and 1 h after needle-induced wounding, including merged images and a magnified view. *Pvps-32-VPS-32::GFP(zjuSi585); Pmyo-3-mCherry(zjuEx3524)* transgenic animals were used. Arrows indicate wound sites. Scale bar, 10 μm.

To determine the physiological significance of VPS-32 during plasma membrane repair, we first perturbed VPS-32 function in the epidermis using RNAi. Knockdown of *vps-32* caused pronounced defects in wound closure, with wounds remaining significantly larger than controls for at least 3 h post-wounding (Fig. 7, A and B). Consistent with a failure in membrane resealing, Trypan blue (TryB) exclusion assays revealed persistent barrier failure in *vps-32* RNAi animals, with extensive dye uptake observed up to 6 h after wounding (Fig. 7 C and Fig. S5 A). These findings demonstrate that VPS-32 is essential for efficient repair of large plasma membrane wounds.

Because ESCRT-III function depends on continuous filament remodeling by the AAA ATPase VPS-4, we next examined whether VPS-4 regulates assembly of the wound-associated ESCRT-III structure. Similar to *vps-32* depletion, RNAi knockdown of *vps-4* or expression of VPS-4(DN) or VPS-32(DN) significantly impaired wound closure and increased TryB permeability after needle wounding (Fig. S5, A–C). Expression of ATPase-dead VPS-4 abolished VPS-32 recruitment to the membrane at wound sites (Fig. S5, D and E). Together, these results indicate that VPS-4-dependent ESCRT-III assemblies are required for efficient repair of large plasma membrane wounds.

We then assessed the function of VPS-32 polymerization using polymerization-defective variants. Epidermal expression of VPS-32(V44W) exerted a DN effect and markedly reduced membrane accumulation at wound sites (Fig. 7, D and E), consistent with defective incorporation of mutant subunits into WT polymers (Backwell and Marsh, 2022; McCullough et al., 2015; McCullough et al., 2018). More importantly, both *vps-32(V44W)* and *vps-32(E94K)* mutants displayed significantly reduced survival at 24 and 48 h following needle wounding compared with WT animals (Fig. 7 F). These findings demonstrate that VPS-32 polymerization is critical not only for membrane repair but also for organismal survival after severe tissue injury.

To determine whether VPS-32-mediated membrane repair extends beyond the epidermis, we examined VPS-32 recruitment in other mechanically challenged tissues, including neurons and muscles, which are exposed to physiological membrane damage and mechanical injury. Following laser-induced axotomy of PLM mechanosensory neurons or laser-induced injury of body-wall muscle, VPS-32::GFP rapidly accumulated at axonal stumps and muscle lesions within 1 h (Fig. 7, G and H; and Fig. S5 F), indicating that wound-associated ESCRT-III structure may represent a general mechanism for membrane damage repair.

Finally, we asked whether VPS-32 polymerization contributes to regeneration following neuronal injury. WT PLM neurons exhibited robust regeneration 24 h after laser axotomy, whereas axon regeneration was significantly impaired in *vps-32(V44W)* mutants (Fig. 7, I and J). Moreover, ∼60% of the *vps-32(V44W)* animals failed to survive within 24 h after axotomy (Fig. 7 K), highlighting the physiological importance of ESCRT-III–mediated membrane repair during neuronal injury. Collectively, these results demonstrate that dynamic VPS-32 polymerization is required for efficient plasma membrane repair, maintenance of membrane barrier integrity, and organismal survival following tissue damage. Furthermore, the rapid recruitment of VPS-32 to injured epidermal, neuronal, and muscle cells suggests that the wound-associated ESCRT-III assembly represents a conserved membrane repair mechanism deployed across multiple tissues *in vivo*.

## Discussion

Preserving plasma membrane integrity is a fundamental challenge in cell biology. While the mechanisms that seal nanoscale lesions are increasingly well understood, how cells repair micron-scale mechanical ruptures remains a major unresolved question. Here, using the *C. elegans* epidermis as a genetically tractable *in vivo* model, we identify a previously unrecognized mechano-adaptive function of the ESCRT-III machinery during large-scale plasma membrane repair. Our data show that ESCRT-III exhibits a wound size–dependent response: whereas small wounds recruit transient, rapidly exchanging VPS-32 assemblies consistent with the canonical membrane scission pathway, large wounds induce a persistent, wound-associated ESCRT-III structure that remains associated with the membrane throughout repair. Rather than functioning solely as a membrane scission machine, this wound-associated ESCRT-III structure is required for membrane tension restoration, efficient vesicle recruitment, and successful repair of large membrane rupture (Fig. 7 L).

A central finding of this study is that VPS-32 polymerization is required for the formation of this persistent wound-associated ESCRT-III assembly. Polymerization-defective VPS-32 mutants are still recruited to wound sites but fail to organize into higher-order structures, demonstrating that recruitment and polymerization are mechanically separable steps. FRAP analysis further revealed that, unlike the rapidly exchanging ESCRT-III assemblies observed at small wounds, the large-wound-associated assembly remains dynamic but exhibits markedly reduced molecular turnover, indicating the formation of a long-lived structure rather than a static polymer. Together, these findings support a model in which the same ESCRT-III polymerization machinery is deployed differently depending on wound geometry and mechanical context. Rather than representing a fundamentally distinct assembly mechanism, the wound-associated structure likely reflects an adaptation of the intrinsic structural plasticity of ESCRT-III, which is known to assemble into spirals, helices, and other higher-order polymers in diverse membrane-remodeling processes (Pfitzner et al., 2020; Pfitzner et al., 2021; Bertin et al., 2020; Stempels et al., 2023).

Our results further suggest that this persistent ESCRT-III assembly serves a mechanical function that is distinct from the classical membrane scission activity of ESCRT-III. Previous studies have shown that membrane mechanics regulate ESCRT-III assembly in multiple contexts. For example, relaxation of membrane tension promotes ESCRT-III recruitment during cytokinetic abscission (Lafaurie-Janvore et al., 2013), while reduced membrane tension facilitates ESCRT-dependent intraluminal vesicle formation on endosomes (Mercier et al., 2020). During nuclear envelope repair, membrane rupture recruits ESCRT machinery to restore nuclear integrity under mechanical stress (Raab et al., 2016; Denais et al., 2016; Wallis et al., 2021). Our findings extend this framework by demonstrating that ESCRT-III is not merely regulated by membrane tension but actively contributes to restoring membrane tension during repair of large plasma membrane ruptures. Notably, disruption of VPS-32 polymerization abolished this recovery without preventing the initial recruitment of VPS-32, indicating that assembly of the higher-order ESCRT-III structure, rather than simple protein accumulation, is required for mechanical stabilization of the wound edge.

How this wound-associated ESCRT-III assembly restores membrane mechanics remains an important question. Our data support a working model in which the circumferential ESCRT-III assembly reinforces the wound perimeter during the early stages of repair, thereby limiting membrane recoil and providing a mechanically stable platform for subsequent membrane remodeling. Importantly, our imaging data support the formation of a persistent circumferential assembly composed of multiple ESCRT-III components that collectively accumulate around the wound margin. Whether this organization represents a continuous polymer or closely spaced higher-order assemblies remains unresolved and will require future structural studies with higher spatial resolution. Likewise, the precise mechanical mechanism by which ESCRT-III contributes to membrane stabilization remains to be determined. One possibility is that distributed ESCRT-III assemblies locally reinforce membrane stiffness around the wound perimeter. Alternatively, the ESCRT-III assembly may function as part of a larger cortical network through mechanical coupling with the plasma membrane, actomyosin cortex (Bement et al., 1993), and vesicle-derived membranes. These possibilities are not mutually exclusive and warrant further investigation.

Our findings also integrate naturally with previous studies of membrane repair, in which large plasma membrane wounds trigger rapid recruitment of Golgi-derived membranes, endosomal compartments, SNARE proteins, and fusogens that collectively contribute to membrane patch formation and wound closure (Meng et al., 2020; Xu et al., 2024; Wang et al., 2022; Shao et al., 2023; Meng et al., 2023). Here, we show that vesicle recruitment occurs in parallel with assembly of the wound-associated ESCRT-III structure. Although a subset of VPS-32 puncta transiently associates with endosomal compartments during the early response, the mature ESCRT-III assembly is largely distinct from vesicular markers, and disruption of vesicle trafficking does not prevent VPS-32 recruitment. Conversely, impairment of VPS-32 polymerization significantly reduces vesicle accumulation at the wound site. Together, these observations support a sequential model in which ESCRT-III–dependent mechanical stabilization and vesicle-mediated membrane addition cooperate to repair large membrane ruptures. In this model, the persistent ESCRT-III assembly may help stabilize the wound edge and create favorable mechanical conditions for efficient membrane fusion and progressive wound closure.

The implications of these findings extend beyond *C. elegans* and provide a broader framework for understanding plasma membrane repair in mechanically stressed tissue. Defective repair of large membrane ruptures contributes to the pathogenesis of numerous human diseases, including muscular dystrophy, ischemia-reperfusion injury, and neurodegenerative disorders. Our observation that VPS-32 is recruited to membrane injuries in both epidermal cells and neurons, together with the requirement for VPS-32 polymerization in axon regeneration, suggests that this mechano-adaptive function of ESCRT-III may be broadly conserved across tissues. In the mammalian system, CHMP4B-mediated membrane stabilization may be particularly important for preserving sarcolemma integrity in skeletal and cardiac muscle (Scheffer et al., 2014), where cells experience repeated mechanical stress. Consistent with this idea, recent work has shown that Vps4a-mediated plasma membrane repair acts as an intrinsic cytoprotective mechanism during cardiac ischemia-reperfusion injury (Huang et al., 2025). Together, these findings suggest that the ability of ESCRT-III to assemble into persistent, higher-order structures may represent a conserved strategy for maintaining membrane integrity under mechanical stress and may provide new mechanistic insights into tissue injury, regeneration, and diseases associated with defective plasma membrane repair.

## Materials and methods

### *C. elegans* strains and maintenance

All *C. elegans* strains were cultured on nematode growth medium (NGM) plates at 20°C on *Escherichia coli* OP50 under standard conditions. The N2 Bristol strain served as the WT. Transgenic strains expressing fluorescently tagged VPS-32 or membrane markers were generated as described below. VPS-32 mutant alleles (V44W or E94K) were generated by CRISPR-Cas9–mediated genome editing and validated by Sanger sequencing. A complete list of strains used in this study is provided in Table S1.

### Plasmid construction and generation of transgenic worms

DNA constructs were generated using recombination cloning (LR reaction) enzyme kits. The pCFJ201, pCFJ210, and pDEST883 vectors were used as backbone plasmids. Primers were designed based on gene-specific sequences. The *Pcol-19* promoter was used for epidermal-specific expression. Transgenic worms were created by injecting constructs at 10 ng/μl with a 50 ng/μl co-injection marker (*Pttx-3-RFP*). SCIs were made using CRISPR-Cas9. Microinjection mixes contained the modified destination plasmid (50–100 ng/μl), the Cas9-sgRNA plasmid (<50 ng/μl; higher concentrations reduced worm survivability and SCI efficiency), and fluorescence co-injection markers (<10 ng/μl). Young adult hermaphrodites were microinjected in both gonads on 4% agarose pads and recovered at 25°C (Xu et al., 2016). Insertions were targeted to specific genomic loci: the ttTi4348 site on chromosome I, the ttTi5605 site on chromosome II, and the cxTi10882 site on chromosome IV (Frøkjær-Jensen et al., 2012). All plasmids and oligonucleotides used in this study are listed in Table S1.

### Needle puncture, laser ablation, and ultrasonic rupture

For needle puncture, micro-glass needles were pulled using a Narishige PC-10 puller. Synchronized young adult worms were wounded manually under a dissection microscope. For laser-induced wounding, worms were mounted on 4% agar pads, immobilized with 12 mM levamisole, and wounded using a Micropoint UV laser. For ultrasonic wounding, young adult animals were washed and collected in 500 μl of M9 buffer within a 1.5-ml microcentrifuge tube. Mechanical wounding was induced using an ultrasonic homogenizer at 10% amplitude. Worms were subjected to three discrete sonication cycles, each comprising a 0.3-s pulse followed by a 3-s quiescent interval. Immediately after wounding, worms were transferred onto a fresh NGM agar plate seeded with OP50 and allowed to recover at 20°C. Wounded animals were manually selected for live imaging at defined time points. Because ultrasonic treatment induces nonspecific tissue damage, this method was used exclusively to assess generalized responses to large-scale membrane disruption.

### Adult epidermal-specific RNAi

Adult epidermal-specific RNAi was performed as follows (Xu and Chisholm, 2011). Synchronized L1 stage worms carrying the *rde-1(ne219)*; *Pcol-19::RDE-1(juIs346)* genotype were transferred onto NGM plates seeded with HT115(DE3) *E. coli* and supplemented with dsRNA-expressing bacteria targeting specific genes, 1 mM isopropyl β-D-1-thiogalactopyranoside (IPTG), and selective antibiotics. They were incubated at 20°C until reaching the young adult stage for further experiments. Related reagents used in the assay are listed in Table S1.

### TryB staining

TryB staining of wounded worms was performed as follows (Meng et al., 2020). Young adult worms were wounded and incubated in 0.4% (wt/vol) TryB for 1 h at 20°C, washed three times in M9 buffer, and imaged using an upright microscope (Nikon, NIS Elements). Worms were classified as TryB-positive when showing circumferential staining at the wound site. Normalized TryB staining rates were derived as follows: (% TryB^+^ wounded cohort)/(1 − % TryB^+^ unwounded), thereby controlling for nonspecific dye uptake in unwounded control animals. At least 100 wound sites were quantified per experiment, with each experiment repeated at least twice. Related reagents used in the assay are listed in Table S1.

### Fluorescent dextran exclusion assay and imaging

FITC-labeled dextrans of 150 kDa (corresponding to hydrodynamic radii of ∼8.5 nm) (HY128868G, MedChemExpress) were employed. Young adult worms were wounded with a microinjection needle. At a series of time points after wounding, the worms were transferred to staining solutions containing the FITC-dextrans diluted in M9 buffer to a final concentration of 1 mg/ml. Incubation was carried out for 10 min at 20°C in the dark. After staining, the worms were washed three times with M9 buffer and imaged using a spinning-disk confocal microscope in Z-stack mode. Related reagents used in the assay are listed in Table S1.

### WGA staining and imaging

Fluorescently conjugated WGA (G1730-100 μl; Servicebio Co., Ltd.) was used to stain the damaged plasma membrane. Wounded young adult worms were transferred to WGA solution diluted 1:50 in M9 buffer at different time points after wounding and incubated for 10 min at 20°C in the dark. Following three washes with M9 buffer, the worms were imaged with a spinning-disk confocal microscope in Z-stack mode. Related reagents used in the assay are listed in Table S1.

### Live imaging

Images were acquired with a Nikon Eclipse Ti spinning-disk confocal microscope equipped with an Andor confocal scanning unit and a 100×/1.46 NA oil immersion objective (Nikon). Imaging was performed at room temperature (20–25°C). Young adult worms were immobilized on 4% agarose pads containing 12 mM levamisole. Images were acquired at 100× magnification with a 0.5-μm interval between Z-stack images. Z-sectioning and time-lapse imaging were controlled using Andor IQ3 software. Fluorescence was excited with 488-nm (GFP/WGA/FITC-dextran), 561-nm (mKate2/mCherry), and 405-nm (tagBFP) laser lines. The imaging region of interest (ROI) was 1,024 × 1,024 pixels. Laser power, exposure time, and camera gain differed between reporters because the brightness of the different fluorescent proteins and fusion constructs varied. For experiments using the same fluorescent protein fused to the same protein of interest and expressed from the same transgene, identical acquisition settings were used. These settings were chosen so that the target fluorescence signal intensity was at least 1,000 arbitrary units (a.u.) while remaining below the detector saturation limit and well above the background signal intensity. Because the same reporter was imaged in any given comparison, all images used for a given quantification and all images shown in the same figure panel were acquired with identical settings.

### Multi-SIM imaging

Super-resolution imaging of *C. elegans* epidermis was performed using a Multi-SIM system (Nano Insights, Beijing) equipped with a 100×/1.49 NA oil immersion objective (Nikon) and sCMOS camera (Photometrics Kinetix). Imaging modes included GI-SIM and 3D-SIM. Imaging was performed at room temperature (20–25°C). Image stacks were reconstructed and deconvolved using SI-Recon 2.23.3 (Nano Insights), with regular calibration performed using 100-nm fluorescent beads to ensure lateral and axial resolution and accurate channel registration. The imaging ROI is 3,072 × 3,072 pixels.

### Image analysis

Fluorescence signals were quantified using ImageJ (Fiji) and partially 3D-enabled by Imaris. For each worm, the absolute intensity of the recruited fluorescence signal was measured. The intensity of unwounded and wounded regions, as well as background intensity, was calculated. The recruitment fluorescence signal was obtained using the formula: (Iw − Iuw)/(Iuw − Ibg).

### Colocalization analysis

Pearson/Manders’ colocalization coefficients were calculated for colocalization analysis. ROIs were selected. The PCC/MCC values indicate the degree of colocalization. The quantitative value of PCC/MCC was calculated in ImageJ Coloc 2.

### FRAP analysis

Signals were bleached using 100% laser transmission with an argon laser (30 mW, 50% at 405 nm). Fluorescence intensity was imaged at 10-s intervals for 20 min. The relative fluorescence intensity was calculated and plotted against time to generate the FRAP curve.

### FRET analysis

FRET experiments were performed on day 1-stage worms using a spinning-disk confocal microscope equipped with a 100×, 1.46 NA objective (Andor). mCherry::KRAS was excited at 561 nm and collected through a 620/50-nm filter, while Lyn::YPet was excited at 488 nm and collected through a 525/50-nm filter. FRET images were acquired by exciting YPet with a 488-nm laser and collecting the emission through a 620/50-nm filter. FRET efficiency was quantified using ImageJ plugin PixFRET.

### Quantitative analysis

Experiments were performed at least three times independently. Statistical analyses were performed using GraphPad Prism 9. The normality of each dataset was assessed using the D’Agostino*–*Pearson omnibus test prior to the selection of parametric or nonparametric tests. Normally distributed data were analyzed using unpaired two-tailed *t* tests or one-way ANOVA (followed by Tukey’s post hoc test for multiple comparisons), and non-normally distributed data were analyzed using the Mann*–*Whitney test or the Kruskal*–*Wallis test (followed by Dunn’s post-hoc test for multiple comparisons). Data are presented as mean ± SEM, and the number of independent groups (n) is indicated in each figure legend. TryB positivity, wound-induced survival, and axotomy-induced mortality are count-based (proportional) measurements; these comparisons were therefore performed on the raw counts of TryB-positive/negative wound sites or of dead/surviving animals rather than on the percentages plotted in the graphs. The proportions of TryB-positive wounds and of dead animals did not differ significantly among the independent groups of a given genotype (P > 0.05, χ-square test for homogeneity), and their counts were consequently pooled into a single 2 × 2 contingency table (genotype × outcome). Genotypes were compared using Pearson’s χ-square test, and Fisher’s exact test was used when any expected cell count was less than five. Each mutant or transgenic line was compared with the corresponding control (WT or L4440) as a pairwise contrast, and P values were adjusted for multiple comparisons using the Bonferroni method. Results were considered significant when P < 0.05. Unless otherwise stated, at least 10 wound sites were quantified per experiment. At least 100 wound sites were scored per group in the TryB exclusion assays, >50 individuals were scored per group in the survival assays, and >20 individuals were scored per group in the mortality assays; n refers to the number of independent groups assayed.

### Online supplemental material

Fig. S1 shows recruitment of upstream ESCRT components (PDCD-6, ALX-1, and TSG-101) and multiple ESCRT-III subunits to wound sites and transient peri-wound VPS-32 recruitment after large laser wounds and reduced molecular turnover of wound-associated VPS-32 revealed by FRAP. Fig. S2 demonstrates that VPS-32 polymerization is required for formation of the wound-associated ESCRT-III assembly, membrane remodeling, and efficient wound closure. Fig. S3 shows that VPS-32 assembly precedes and is largely independent of vesicle recruitment, based on WGA staining, dextran permeability assays, vesicle marker localization, and vesicle trafficking perturbations. Fig. S4 validates the FRET-based membrane tension sensor and demonstrates that VPS-32 polymerization is required for restoration of membrane tension following large plasma membrane injury. Fig. S5 demonstrates that VPS-32 is broadly required for plasma membrane repair in epidermal and muscle tissues using TryB staining or imaging. Videos 1, 2, and 3 show VPS-32::GFP dynamics following small laser, large laser, and needle-induced wounding, respectively. Video 4 shows disassembly of the wound-associated VPS-32 structure during repair. Videos 5, 6, and 7 show VPS-32 recruitment relative to a plasma membrane marker after small laser, large laser, and needle-induced wounding. Videos 8, 9, and 10 show FRAP analyses of VPS-32 in unwounded epidermis and following small and large plasma membrane wounding. Table S1 shows the reagents, *C. elegans* strains, plasmids, and primers used in this study.

## Supplementary Material

Review History

Table S1 shows the reagents, *C. elegans* strains, plasmids, and primers used in this study.

## References

[bib1] Ammendolia, D.A., W.M. Bement, and J.H. Brumell. 2021. Plasma membrane integrity: Implications for health and disease. BMC Biol. 19:71. 10.1186/s12915-021-00972-y PMC8042475 33849525

[bib2] Backwell, L., and J.A. Marsh. 2022. Diverse molecular mechanisms underlying pathogenic protein mutations: Beyond the loss-of-function paradigm. Annu. Rev. Genomics Hum. Genet. 23:475–498. 10.1146/annurev-genom-111221-103208 35395171

[bib3] Bement, W.M., P. Forscher, and M.S. Mooseker. 1993. A novel cytoskeletal structure involved in purse string wound closure and cell polarity maintenance. J. Cell Biol. 121:565–578. 10.1083/jcb.121.3.565 PMC2119560 8486737

[bib4] Bertin, A., N. de Franceschi, E. de la Mora, S. Maity, M. Alqabandi, N. Miguet, A. di Cicco, W.H. Roos, S. Mangenot, W. Weissenhorn, and P. Bassereau. 2020. Human ESCRT-III polymers assemble on positively curved membranes and induce helical membrane tube formation. Nat. Commun. 11:2663. 10.1038/s41467-020-16368-5 PMC7260177 32471988

[bib5] Booth, A., C.J. Marklew, B. Ciani, and P.A. Beales. 2019. In vitro membrane remodeling by escrt is regulated by negative feedback from membrane tension. iScience. 15:173–184. 10.1016/j.isci.2019.04.021 PMC6503128 31060000

[bib6] Burigotto, M., and J.G. Carlton. 2026. ESCRT-III function in membrane fission and repair. Nat. Rev. Mol. Cell Biol. 27:297–315. 10.1038/s41580-025-00909-1 PMC7618577 41299081

[bib7] Chen, W., M.M. Motsinger, J. Li, K.P. Bohannon, and P.I. Hanson. 2024. Ca2+-sensor ALG-2 engages ESCRTs to enhance lysosomal membrane resilience to osmotic stress. Proc. Natl. Acad. Sci. USA. 121:e2318412121. 10.1073/pnas.2318412121 PMC11145288 38781205

[bib8] Cooper, S.T., and P.L. McNeil. 2015. Membrane repair: Mechanisms and pathophysiology. Physiol. Rev. 95:1205–1240. 10.1152/physrev.00037.2014 PMC4600952 26336031

[bib9] Corkery, D.P., D. Wijayatunga, B.K.L. Feron, L.K. Herzog, A. Knyazeva, and Y.-W. Wu. 2026. The ATG8 E3-like ligases sense lysosomal damage and initiate ESCRT-mediated membrane repair. EMBO J. 45:930–952. 10.1038/s44318-025-00672-1 PMC12865045 41484365

[bib10] Denais, C.M., R.M. Gilbert, P. Isermann, A.L. McGregor, M. te Lindert, B. Weigelin, P.M. Davidson, P. Friedl, K. Wolf, and J. Lammerding. 2016. Nuclear envelope rupture and repair during cancer cell migration. Science. 352:353–358. 10.1126/science.aad7297 PMC4833568 27013428

[bib11] Frøkjær-Jensen, C., M.W. Davis, M. Ailion, and E.M. Jorgensen. 2012. Improved mos1-mediated transgenesis in *C. elegans*. Nat. Methods. 9:117–118. 10.1038/nmeth.1865 PMC3725292 22290181

[bib12] Fu, H., H. Zhou, X. Yu, J. Xu, J. Zhou, X. Meng, J. Zhao, Y. Zhou, A.D. Chisholm, and S. Xu. 2020. Wounding triggers MIRO-1 dependent mitochondrial fragmentation that accelerates epidermal wound closure through oxidative signaling. Nat. Commun. 11:1050. 10.1038/s41467-020-14885-x PMC7044169 32103012

[bib13] Gatta, A.T., and J.G. Carlton. 2019. The ESCRT-machinery: Closing holes and expanding roles. Curr. Opin. Cell Biol. 59:121–132. 10.1016/j.ceb.2019.04.005 31132588

[bib14] Goul, C.S., A. Jain, S. Yitiz, Z.E. Soltani, S. Yang, S. Rapp, M. Spacci, S. Federman, J. Sacco, H. Li, . 2026. LASER couples damage sensing to ESCRT assembly for lysosome repair. Nature. 656:1–11. 10.1038/s41586-026-10604-6 PMC13442042 42236937

[bib15] Harker-Kirschneck, L., B. Baum, and A. Šarić. 2019. Changes in ESCRT-III filament geometry drive membrane remodelling and fission in silico. BMC Biol. 17:82. 10.1186/s12915-019-0700-2 PMC6806514 31640700

[bib16] Heffner, C.M., G.P. Starling, L.C. Straker, P.C. Hawes, A.M. Isaacs, and J.G. Carlton. 2026. The ALS- and FTD-associated proteins annexin A11 and CHMP2B act sequentially in plasma membrane repair. Dev. Cell. 61:1487–1503.e7. 10.1016/j.devcel.2026.05.014 PMC13374510 42349418

[bib17] Henne, W.M., H. Stenmark, and S.D. Emr. 2013. Molecular mechanisms of the membrane sculpting ESCRT pathway. Cold Spring Harb. Perspect. Biol. 5:a016766. 10.1101/cshperspect.a016766 PMC3753708 24003212

[bib18] Huang, X., J. Zhang, C. Xu, R. Cao, P. Jiang, X. Ji, W. Wang, Z. Huang, and P. Han. 2025. Vps4a mediates a unified membrane repair machinery to attenuate ischemia/reperfusion injury. Circ. Res. 136:279–296. 10.1161/CIRCRESAHA.124.325290 39764631

[bib19] Huang, Y., X. Zhang, H.-W. Wang, and L. Yu. 2022. Assembly of tetraspanin-enriched macrodomains contains membrane damage to facilitate repair. Nat. Cell Biol. 24:825–832. 10.1038/s41556-022-00920-0 35654840

[bib20] Hurley, J.H., A.N. Coyne, M. Miączyńska, and H. Stenmark. 2025. The expanding repertoire of ESCRT functions in cell biology and disease. Nature. 642:877–888. 10.1038/s41586-025-08950-y 40562928

[bib21] Hurley, J.H., and S.D. Emr. 2006. The ESCRT complexes: Structure and mechanism of a membrane-trafficking network. Annu. Rev. Biophys. Biomol. Struct. 35:277–298. 10.1146/annurev.biophys.35.040405.102126 PMC1648078 16689637

[bib22] Jimenez, A.J., P. Maiuri, J. Lafaurie-Janvore, S. Divoux, M. Piel, and F. Perez. 2014. ESCRT machinery is required for plasma membrane repair. Science. 343:1247136. 10.1126/science.1247136 24482116

[bib23] Jukic, N., A.P. Perrino, L. Redondo-Morata, and S. Scheuring. 2023. Structure and dynamics of ESCRT-III membrane remodeling proteins by high-speed atomic force microscopy. J. Biol. Chem. 299:104575. 10.1016/j.jbc.2023.104575 PMC10074808 36870686

[bib24] Kozlov, M.M., and L.V. Chernomordik. 2015. Membrane tension and membrane fusion. Curr. Opin. Struct. Biol. 33:61–67. 10.1016/j.sbi.2015.07.010 PMC4641764 26282924

[bib25] Lafaurie-Janvore, J., P. Maiuri, I. Wang, M. Pinot, J.-B. Manneville, T. Betz, M. Balland, and M. Piel. 2013. ESCRT-III assembly and cytokinetic abscission are induced by tension release in the intercellular bridge. Science. 339:1625–1629. 10.1126/science.1233866 23539606

[bib26] Liu, M., Y. Liu, T. Song, L. Yang, L. Qi, Y.-Z. Zhang, Y. Wang, and Q.-T. Shen. 2024. Three-dimensional architecture of ESCRT-III flat spirals on the membrane. Proc. Natl. Acad. Sci. USA. 121:e2319115121. 10.1073/pnas.2319115121 PMC11098116 38709931

[bib27] McCullough, J., A.K. Clippinger, N. Talledge, M.L. Skowyra, M.G. Saunders, T.V. Naismith, L.A. Colf, P. Afonine, C. Arthur, W.I. Sundquist, . 2015. Structure and membrane remodeling activity of ESCRT-III helical polymers. Science. 350:1548–1551. 10.1126/science.aad8305 PMC4684769 26634441

[bib28] McCullough, J., A. Frost, and W.I. Sundquist. 2018. Structures, functions, and dynamics of ESCRT-III/Vps4 membrane remodeling and fission complexes. Annu. Rev. Cell Dev. Biol. 34:85–109. 10.1146/annurev-cellbio-100616-060600 PMC6241870 30095293

[bib29] McNeil, P.L., and T. Kirchhausen. 2005. An emergency response team for membrane repair. Nat. Rev. Mol. Cell Biol. 6:499–505. 10.1038/nrm1665 15928713

[bib30] McNeil, P.L., and R.A. Steinhardt. 2003. Plasma membrane disruption: Repair, prevention, adaptation. Annu. Rev. Cell Dev. Biol. 19:697–731. 10.1146/annurev.cellbio.19.111301.140101 14570587

[bib31] Meng, X., C.S. Wijaya, Q. Shao, and S. Xu. 2023. Triggered Golgi membrane enrichment promotes PtdIns(4,5)P2 generation for plasma membrane repair. J. Cell Biol. 222:e202303017. 10.1083/jcb.202303017 PMC10176212 37158801

[bib32] Meng, X., Q. Yang, X. Yu, J. Zhou, X. Ren, Y. Zhou, and S. Xu. 2020. Actin polymerization and ESCRT trigger recruitment of the fusogens Syntaxin-2 and EFF-1 to promote membrane repair in *C. elegans*. Dev. Cell. 54:624–638.e5. 10.1016/j.devcel.2020.06.027 32668210

[bib33] Mercier, V., J. Larios, G. Molinard, A. Goujon, S. Matile, J. Gruenberg, and A. Roux. 2020. Endosomal membrane tension regulates ESCRT-III-dependent intra-lumenal vesicle formation. Nat. Cell Biol. 22:947–959. 10.1038/s41556-020-0546-4 PMC7612185 32753669

[bib34] Natsuka, S., M. Kawaguchi, Y. Wada, A. Ichikawa, K. Ikura, and S. Hase. 2005. Characterization of wheat germ agglutinin ligand on soluble glycoproteins in *Caenorhabditis elegans*. J. Biochem. 138:209–213. 10.1093/jb/mvi117 16091596

[bib35] Olmos, Y. 2022. The ESCRT machinery: Remodeling, repairing, and sealing membranes. Membranes. 12:633. 10.3390/membranes12060633 PMC9229795 35736340

[bib36] Park, J., J. Kim, H. Park, T. Kim, and S. Lee. 2024. ESCRT-III: A versatile membrane remodeling machinery and its implications in cellular processes and diseases. Anim. Cells Syst. 28:367–380. 10.1080/19768354.2024.2380294 PMC11275535 39070887

[bib37] Pfitzner, A.-K., J.M. von Filseck, and A. Roux. 2021. Principles of membrane remodeling by dynamic ESCRT-III polymers. Trends Cell Biol. 31:856–868. 10.1016/j.tcb.2021.04.005 33980463

[bib38] Pfitzner, A.-K., V. Mercier, X. Jiang, J.M. von Filseck, B. Baum, A. Šarić, and A. Roux. 2020. An ESCRT-III polymerization sequence drives membrane deformation and fission. Cell. 182:1140–1155.e18. 10.1016/j.cell.2020.07.021 PMC7479521 32814015

[bib39] Pontes, B., P. Monzo, and N.C. Gauthier. 2017. Membrane tension: A challenging but universal physical parameter in cell biology. Semin. Cell Dev. Biol. 71:30–41. 10.1016/j.semcdb.2017.08.030 28851599

[bib40] Raab, M., M. Gentili, H. de Belly, H.-R. Thiam, P. Vargas, A.J. Jimenez, F. Lautenschlaeger, R. Voituriez, A.-M. Lennon-Duménil, N. Manel, and M. Piel. 2016. ESCRT III repairs nuclear envelope ruptures during cell migration to limit DNA damage and cell death. Science. 352:359–362. 10.1126/science.aad7611 27013426

[bib41] Radulovic, M., K.O. Schink, E.M. Wenzel, V. Nähse, A. Bongiovanni, F. Lafont, and H. Stenmark. 2018. ESCRT-mediated lysosome repair precedes lysophagy and promotes cell survival. EMBO J. 37:e99753. 10.15252/embj.201899753 PMC6213280 30314966

[bib42] Raj, N., M.S. Weiß, B.E. Vos, S. Weischer, F. Brinkmann, T. Betz, B. Trappmann, and V. Gerke. 2024. Membrane tension regulation is required for wound repair. Adv. Sci. 11:e2402317. 10.1002/advs.202402317 PMC11672324 39360573

[bib43] Ringer, P., A. Weißl, A.-L. Cost, A. Freikamp, B. Sabass, A. Mehlich, M. Tramier, M. Rief, and C. Grashoff. 2017. Multiplexing molecular tension sensors reveals piconewton force gradient across talin-1. Nat. Methods. 14:1090–1096. 10.1038/nmeth.4431 28945706

[bib44] Scheffer, L.L., S.C. Sreetama, N. Sharma, S. Medikayala, K.J. Brown, A. Defour, and J.K. Jaiswal. 2014. Mechanism of Ca2+-triggered ESCRT assembly and regulation of cell membrane repair. Nat. Commun. 5:5646. 10.1038/ncomms6646 PMC4333728 25534348

[bib45] Schmidt, O., Y. Weyer, S. Sprenger, M.A. Widerin, S. Eising, V. Baumann, M. Angelova, R. Loewith, C.J. Stefan, M.W. Hess, . 2020. TOR complex 2 (TORC2) signaling and the ESCRT machinery cooperate in the protection of plasma membrane integrity in yeast. J. Biol. Chem. 295:12028–12044. 10.1074/jbc.ra120.013222 PMC7443507 32611771

[bib46] Schöneberg, J., M.R. Pavlin, S. Yan, M. Righini, I.H. Lee, L.A. Carlson, A.H. Bahrami, D.H. Goldman, X. Ren, G. Hummer, . 2018. ATP-dependent force generation and membrane scission by ESCRT-III and Vps4. Science. 362:1423–1428. 10.1126/science.aat1839 PMC6309985 30573630

[bib47] Shao, Q., C.S. Wijaya, S. Wang, X. Meng, C. Yuan, C. Ma, and S. Xu. 2023. The SNARE complex formed by RIC-4/SEC-22/SYX-2 promotes *C. elegans* epidermal wound healing. Cell Rep. 42:113349. 10.1016/j.celrep.2023.113349 37910502

[bib48] Shen, Q.-T., A.L. Schuh, Y. Zheng, K. Quinney, L. Wang, M. Hanna, J.C. Mitchell, M.S. Otegui, P. Ahlquist, Q. Cui, and A. Audhya. 2014. Structural analysis and modeling reveals new mechanisms governing ESCRT-III spiral filament assembly. J. Cell Biol. 206:763–777. 10.1083/jcb.201403108 PMC4164947 25202029

[bib49] Shukla, S., W. Chen, S. Rao, S. Yang, C. Ou, K.P. Larsen, G. Hummer, P.I. Hanson, and J.H. Hurley. 2024. Mechanism and cellular function of direct membrane binding by the ESCRT and ERES-associated Ca2+-sensor ALG-2. Proc. Natl. Acad. Sci. USA. 121:e2318046121. 10.1073/pnas.2318046121 PMC10907313 38386713

[bib50] Skowyra, M.L., P.H. Schlesinger, T.V. Naismith, and P.I. Hanson. 2018. Triggered recruitment of ESCRT machinery promotes endolysosomal repair. Science. 360:eaar5078. 10.1126/science.aar5078 PMC6195421 29622626

[bib51] Stempels, F.C., M. Jiang, H.M. Warner, M.-L. Moser, M.H. Janssens, S. Maassen, I.H. Nelen, R. de Boer, W.F. Jiemy, D. Knight, . 2023. Giant worm-shaped ESCRT scaffolds surround actin-independent integrin clusters. J. Cell Biol. 222:e202205130. 10.1083/jcb.202205130 PMC10200693 37200023

[bib52] Tang, S., W.M. Henne, P.P. Borbat, N.J. Buchkovich, J.H. Freed, Y. Mao, J.C. Fromme, and S.D. Emr. 2015. Structural basis for activation, assembly and membrane binding of ESCRT-III Snf7 filaments. Elife. 4:e12548. 10.7554/elife.12548 PMC4720517 26670543

[bib53] Tang, S.K.Y., and W.F. Marshall. 2017. Self-repairing cells: How single cells heal membrane ruptures and restore lost structures. Science. 356:1022–1025. 10.1126/science.aam6496 PMC5664224 28596334

[bib54] Togo, T., J.M. Alderton, G.-Q. Bi, and R.A. Steinhardt. 1999. The mechanism of facilitated cell membrane resealing. J. Cell Sci. 112:719–731. 10.1242/jcs.112.5.719 9973606

[bib55] Vietri, M., M. Radulovic, and H. Stenmark. 2020. The many functions of ESCRTs. Nat. Rev. Mol. Cell Biol. 21:25–42. 10.1038/s41580-019-0177-4 31705132

[bib56] Wallis, S.S., L.N. Ventimiglia, E. Otigbah, E. Infante, M.A. Cuesta-Geijo, G.R. Kidiyoor, M.A. Carbajal, R.A. Fleck, M. Foiani, S. Garcia-Manyes, . 2021. The ESCRT machinery counteracts Nesprin-2G-mediated mechanical forces during nuclear envelope repair. Dev. Cell. 56:3192–3202.e8. 10.1016/j.devcel.2021.10.022 PMC8657813 34818527

[bib57] Wang, Y., Q. Yang, X. Meng, C.S. Wijaya, X. Ren, and S. Xu. 2022. Recruitment of tetraspanin TSP-15 to epidermal wounds promotes plasma membrane repair in *C. elegans*. Dev. Cell. 57:1630–1642.e4. 10.1016/j.devcel.2022.06.004 35777354

[bib58] Wijaya, C.S., and S. Xu. 2024. Reevaluating Golgi fragmentation and its implications in wound repair. Cell Regen. 13:4. 10.1186/s13619-024-00187-w PMC10864233 38349608

[bib59] Xu, J., X. Meng, Q. Yang, J. Zhang, W. Hu, H. Fu, J.W. Chen, W. Ma, A.D. Chisholm, Q. Sun, and S. Xu. 2021. Redox-sensitive CDC-42 clustering promotes wound closure in *C. elegans*. Cell Rep. 37:110040. 10.1016/j.celrep.2021.110040 PMC8742831 34818546

[bib60] Xu, S., and A.D. Chisholm. 2011. A Gαq-Ca2+ signaling pathway promotes actin-mediated epidermal wound closure in *C. elegans*. Curr. Biol. 21:1960–1967. 10.1016/j.cub.2011.10.050 PMC3237753 22100061

[bib61] Xu, S., and A.D. Chisholm. 2014. *C. elegans* epidermal wounding induces a mitochondrial ros burst that promotes wound repair. Dev. Cell. 31:48–60. 10.1016/j.devcel.2014.08.002 PMC4197410 25313960

[bib62] Xu, S., Z. Wang, K.W. Kim, Y. Jin, and A.D. Chisholm. 2016. Targeted mutagenesis of duplicated genes in *Caenorhabditis elegans* using CRISPR-Cas9. J. Genet. Genomics. 43:103–106. 10.1016/j.jgg.2015.11.004 PMC5291165 26924693

[bib63] Xu, S., T.J. Yang, S. Xu, and Y.-N. Gong. 2024. Plasma membrane repair empowers the necrotic survivors as innate immune modulators. Semin. Cell Dev. Biol. 156:93–106. 10.1016/j.semcdb.2023.08.001 PMC10872800 37648621

