# Supplementary material for: ESCRT-III assembly restores membrane tension in micron-scale plasma membrane repair
Source: J Cell Biol. 2026 Nov 13;226(2):e202601076. doi: 10.1083/jcb.202601076 (PMC13762749; doi:10.1083/jcb.202601076)
Supplement: Supplementary file 1 [file jcb_202601076_review_history.pdf]

ESCRT-III assembly restores membrane tension in micron-scale plasma membrane repair

Xinan Meng, Yuanyuan Wang, Erqing Gao, Liwei Guan, Yiyuan Ge, Jingyi Kang, Jingxiu Xu, Jingyang Yuan, Shujun Cai, Eric Miska, Wei Yang, and Suhong Xu

Corresponding Author(s): Suhong Xu, Zhejiang University and Xinan Meng, Zhejiang University

Review Timeline:

Submission Date:	2026-01-12
Editorial Decision:	2026-03-24
Revision Received:	2026-08-11
Editorial Decision:	2026-10-06
Revision Received:	2026-10-14

Monitoring Editor: Li Yu

Scientific Editor: Therese Gerbich

Transaction Report:

(Note: With the exception of the correction of typographical or spelling errors that could be a source of ambiguity, letters and reports are not edited. The original formatting of letters and referee reports may not be reflected in this compilation.)

DOI: https://doi.org/10.1083/jcb.202601076

March 24, 2026

Re: JCB manuscript #202601076

Suhong Xu
Zhejiang University

Dear Prof. Xu,

Thank you for submitting your manuscript entitled "VPS-32 polymerization restores membrane tension to enable large-scale plasma membrane repair". Your manuscript has been assessed by expert reviewers, whose comments are appended below. Although the reviewers express potential interest in this work, significant concerns unfortunately preclude publication of the current version of the manuscript in JCB.

You will see that all three reviewers found the work to be exciting and of high potential impact, with strengths in both the conceptual advance and experimental approach. At the same time, the reviewers have raised a number of important concerns that will need to be addressed before the manuscript can be considered further. In particular, there is a shared view that the current evidence supporting the proposed mechanistic model is not yet sufficiently developed, and that additional clarification and experimental support are required.

In your revision, we ask that you more clearly define your model for ESCRT-III function in membrane repair, including how it relates to established roles in membrane scission. The reviewers ask for a more rigorous and explicit treatment of how ESCRT-III assemblies form, how they might generate or stabilize membrane tension, and how recruitment is distinguished from polymerization. Both reviewers 1 and 2 also ask that you examine or discuss the involvement of other ESCRT components. While we recognize that fully addressing all of these points experimentally may be challenging within a single revision, we ask that you prioritize the most critical aspects and provide additional data and/or analysis that meaningfully strengthen the mechanistic framework and interpretation of your findings.

We also ask that you address central concern of Rev #1 is the interpretation of diffraction-limited puncta as "supramolecular polymers." The reviewer requests stronger evidence to support this claim. Approaches such as higher-resolution structural methods (e.g., CLEM or electron tomography) or complementary in vitro reconstitution assays would substantially strengthen the manuscript. While we recognize that some of these experiments may be technically demanding, additional data that directly address the structural nature of these assemblies will be important.

Finally, the reviewers feel that then manuscript would benefit from a more comprehensive discussion that situates your findings within the broader ESCRT field and addresses some alternative interpretations.

Taken together, the revisions requested are substantial and may require significant additional experimentation. We encourage you to carefully consider the full set of reviewer comments in determining the scope of your revision. If certain experiments are not feasible within a reasonable timeframe, please provide a clear rationale and where possible, alternative supporting evidence or a more tempered interpretation.

Please let us know if you are able to address the major issues outlined above and wish to submit a revised manuscript to JCB. Note that a substantial amount of additional experimental data likely would be needed to satisfactorily address the concerns of the reviewers. The typical timeframe for revisions is three to four months. If you anticipate any difficulties in meeting this aforementioned revision time limit, please contact us and we can work with you to find an appropriate time frame for resubmission. Please note that papers are generally considered through only one revision cycle, so any revised manuscript will likely be either accepted or rejected.

If you choose to revise and resubmit your manuscript, please also attend to the following editorial points. Please direct any editorial questions to the journal office.

GENERAL GUIDELINES:
Text limits: Character count is < 40,000, not including spaces. Count includes title page, abstract, introduction, results, discussion, and acknowledgments. Count does not include materials and methods, figure legends, references, tables, or supplemental legends.

Figures: Your manuscript may have up to 10 main text figures. To avoid delays in production, figures must be prepared according to the policies outlined in our Instructions to Authors, under Data Presentation, https://jcb.rupress.org/site/misc/ifora.xhtml. All figures in accepted manuscripts will be screened prior to publication.

***IMPORTANT: It is JCB policy that if requested, original data images must be made available. Failure to provide original

images upon request will result in unavoidable delays in publication. Please ensure that you have access to all original microscopy and blot data images before submitting your revision.***

Supplemental information: There are strict limits on the allowable amount of supplemental data. Your manuscript may have up to 5 supplemental figures. Up to 10 supplemental videos or flash animations are allowed. A summary of all supplemental material should appear at the end of the Materials and methods section.

Please note that JCB now requires authors to submit Source Data used to generate figures containing gels and Western blots with all revised manuscripts. This Source Data consists of fully uncropped and unprocessed images for each gel/blot displayed in the main and supplemental figures. For assays performed using capillary electrophoresis and/or immunoassay-based detection, authors should instead provide the electropherogram graph(s) for each experiment, plotting fluorescence/chemiluminescence intensity vs. molecular weight/size. Please be sure to provide one Source Data file for each figure gels, blots, and/or capillary electrophoresis assays along with your revised manuscript files. File names for Source Data figures should be alphanumeric without any spaces or special characters (i.e., SourceDataF#, where F# refers to the associated main figure number or SourceDataFS# for those associated with Supplementary figures). For traditional gels and blots, the lanes of the gels/blots should be labeled as they are in the associated figure, the place where cropping was applied should be marked (with a box), and molecular weight/size standards should be labeled wherever possible. For capillary electrophoresis assays, each trace in the graph should be color-coded and labeled to indicate which protein, gene, or sample is being measured (please try to avoid red/green combinations to accommodate our color-blind readers).

Source Data files will be made available to reviewers during evaluation of revised manuscripts and, if your paper is eventually published in JCB, the files will be directly linked to specific figures in the published article.

Source Data Figures should be provided as individual PDF files (one file per figure). Authors should endeavor to retain a minimum resolution of 300 dpi or pixels per inch. Please review our instructions for export from Photoshop, Illustrator, and PowerPoint here: https://rupress.org/jcb/pages/submission-guidelines#revised

If you choose to resubmit, please include a cover letter addressing the reviewers' comments point by point. Please also highlight all changes in the text of the manuscript.

Regardless of how you choose to proceed, we hope that the comments below will prove constructive as your work progresses. We would be happy to discuss them further once you've had a chance to consider the points raised. You can contact the journal office with any questions at cellbio@rockefeller.edu.

Thank you for thinking of JCB as an appropriate place to publish your work.

Sincerely,

Li Yu
Monitoring Editor
Journal of Cell Biology

Therese Gerbich
Scientific Editor
Journal of Cell Biology

Reviewer #1 (Comments to the Authors (Required)):

Meng et al. report that the ESCRT-III protein VPS-32 responds to large-scale (10 um) plasma membrane wounds in C.elegans epidermis to form large polymers that encircle the wound. VPS-32 recruitment to large wounds was substantially slower than recruitment to smaller (1 um) wounds. Using a FRET-based sensor module the authors found that VPS-32 polymerization is required in order to restore membrane tension during repair. The requirement for VPS-32 in repair of large plasma membrane wounds was not restricted to C.elegans epidermis but was also shown to be true for wounded muscles and axons.

This is an interesting study that reveals a non-canonical function for ESCRT-III filaments. Overall the conclusions are well supported by the data. However, the authors need to address the following issues prior to eventual publication.

Major points:

1. Repair of small plasma membrane wounds has previously been shown to involve ESCRT components upstream of ESCRT-III

such as ALIX and TSG101. The authors need to examine whether this is the case for large wounds as well.

2. It is surprising that large plasma membrane wounds can persist for up to 60 minutes without causing cell death. The authors should investigate if there is something plugging the holes, such as glycocalyx.

Minor point:

The research literature is in general cited adequately, but in the fast-moving ESCRT field it seems strange to cite reviews from 2006, 2011 and 2013. The authors should either cite newer reviews or refer directly to the relevant original articles.

Reviewer #2 (Comments to the Authors (Required)):

In this manuscript, Meng et al. investigated the recruitment and function of VPS-32/CHMP4B in plasma membrane repair of the C. elegans hyp7 epidermis. Most previous ESCRT-III mediated membrane repair studies have been carried out in cultured cells, so it is exciting to see VPS-32 recruitment and membrane repair investigated in a living organism. By combining elegant reporters, genetics, time-lapse imaging and different types of membrane damage, the authors clearly show that plasma membrane wounds recruit VPS-32 and that this recruitment is required for efficient repair.
However, I find the mechanistic interpretation of how VPS-32 mediates repair not fully convincing at this stage, and the upstream recruitment mechanism as well as the role of other ESCRT-III subunits remain largely unexplored. This makes the work perhaps less novel than it should be. I would suggest that the authors address the following key points to strengthen their claims:
Major points
Evidence for a "supramolecular polymer" encircling the wound: Instead of invoking the canonical ESCRT-III role in neck fission/budding, the authors propose that VPS-32 assembles into a massive supramolecular polymer that forms a ring around large wound. At present, there is no direct evidence for this central claim. In all VPS-32 images presented, one mainly sees individual puncta that cluster and appear to accumulate/tether around the wound; continuous ring-like structures are not clearly resolved. This phenotype could also be explained by a canonical "vesicle-vesicle fusion" model of plasma membrane repair, in which multiple VPS-32-positive vesicles dock and fuse near the wound.
It is not sufficient to define a "supramolecular polymer" solely from diffraction-limited fluorescence puncta. Because hyp7 epidermal cells and their wounds are very large, this system would be ideal to address this question more directly by CLEM / electron tomography to visualise ESCRT-III assemblies at the wound edge. Alternatively, an in vitro VPS-32 self-assembly assay on giant vesicles could provide more direct evidence for large continuous polymers encircling the wound.
The authors show that VPS-32 mutants that impair ESCRT-III filament formation display much reduced recruitment. If the model is that VPS-32 puncta first accumulate and then assemble into a supramolecular polymer, one might expect filament-defective mutants to still be recruited to the wound as individual puncta, but to fail in forming larger, more continuous structures. At present it is difficult to distinguish whether the mutants are defective in sensing / recruitment or in polymer assembly, because the upstream recruitment mechanism has not been characterised. A clearer separation of "recruitment" and "polymerisation" would greatly help the interpretation.

It is potentially very informative to understand whether and how other ESCRT-III components regulate VPS-32 recruitment and repair, but the data are hard to follow. In the figures, other ESCRT-III subunits also appear as individual puncta at the wound, with different sizes/intensities compared to VPS-32. Why do ESCRT-III components that are thought to act together show such distinct patterns? Please comment on this.
The VPS-4 part is currently confusing. For VPS-4, the only apparent recruitment image seems to be Figure S5C (VPS-4::mCherry, according to the legend), but the text mentions an ATPase-dead mutant, while the figure label uses "VPS-4(DN)", and the red signal appears to be a membrane marker. It is not clear which signal corresponds to VPS-4. The title of Figure S2 is "VPS-4 is required for VPS-32 recruitment to damaged membrane", but the data supporting this statement are not clearly presented. If VPS-4 is indeed required for VPS-32 recruitment, this would imply that VPS-32 accumulation depends on ESCRT-III complex assembly and VPS-4 cycling, not only on VPS-32 itself. Combined with the recruitment of other ESCRT-III subunits (Fig. 1G), one might even conclude that the proposed "supramolecular polymer" is a property of the entire ESCRT-III ensemble rather than VPS-32 alone. If this is the case, is this behaviour specific to C. elegans, and how does it relate to the models derived from mammalian cells?
The idea that polymeric VPS-32 acts as a scaffold to restore membrane tension at the wound margin is interesting, and the use of a membrane tension probe is a strong point. However, increased membrane tension is not unique to the "polymer ring" model. In principle, any ESCRT-III-mediated constriction/fission/budding mechanism that shrinks the wound area could also lead to a local increase in tension. The authors should acknowledge this and explain why the observed changes in tension specifically support their scaffold model rather than alternative ESCRT-III mechanisms.
In contrast to the fission/budding models proposed in mammalian ESCRT-mediated PM repair, the VPS-32 puncta in C. elegans hyp7 epidermis appear more like vesicles docking and fusing with each other at the wound. This would be compatible with a canonical "vesicle-vesicle fusion" model, as described for example in sea urchin eggs and skeletal muscle repair, where Ca^{2+}-triggered vesicles fuse to form a membrane patch. If no solid structural evidence for a continuous supramolecular polymer can be provided, the authors may need to explore this alternative repair mechanism more explicitly or at least discuss how their

data could fit into a vesicle-fusion-based patch model.
Minor
In several figures the labelling is confusing. Since the authors use three different types of damage, it would be very helpful to clearly label the damage type in each panel (e.g. in Fig. 1G, one had to infer the damage type by comparing scale bars and conditions). As mentioned above, Fig. S5C and the description of VPS-4 are particularly confusing. Mis-labelling or incomplete figure legends significantly undermines confidence in the data and should be carefully corrected throughout.
Late time points and disassembly. It would be valuable to know what happens at later time points: does VPS-32 disassemble and revert to small puncta once repair is complete? This aspect is difficult to capture in mammalian cell systems and could be a real strength of the C. elegans model if analysed properly. A more detailed analysis and discussion of VPS-32 dynamics at late time points would considerably strengthen the mechanistic insight.

Reviewer #3 (Comments to the Authors (Required)):

This paper investigates the assembly dynamics and function of ESCRT-III at sites of plasma membrane damage in the C. Elegans epidermis. By imaging VPS-32 (CHMP-4B homolog), the authors find that after large wounding (10 um) but not small wounding (1 um), causes ESCRT-III to form large puncta up to 50 um from the wound site. These puncta merge and migrate towards the wound, finally forming a ring encircling the wound site. After introducing VPS-32 mutants that have defects in polymerization, the authors show that membrane repair is defective, that the wound has slower closure rate, and that VPS-32 is recruited poorly and does not form elongated structures near the wound site. Finally, using a FRET membrane tension sensor after introduction of VPS-32 mutants, the authors conclude that ESCRT-III recovers membrane tension after PM damage by forming a massive polymer that encircles the wound site.

While this paper presents intriguing observations and ideas, it requires several revisions and controls.

1. Some of the figure legends don't match the figure. See figure S2 for example.

2. Some of the citations have been added as superscript numbers. For example the citation for the specific FRET sensor used is added as citation #32, so I cannot look up what you are referencing.

3. The videos are not titled as video #1, #2, #3, etc nor are they uploaded in a way that matches the order in the paper.

4. The model at the end is not clear. Please elaborate your model in the discussion and be more explicit about your predictions:
A. Is the idea that ESCRT-III subunits form a more static polymer structure that contain large wounds? If so, the authors should perform FRAP on VPS-32 at small vs large wound sites to show there is difference in ESCRT-III cycling at large vs small wound cites. On the other hand, if the idea is that ESCRT-III is cycling via the well-known spiral/dome mechanism, how is it thought that ESCRT-III self-associates to form these higher-order polymers? Is there precedent in vitro for these types of very large ESCRT structures?
B. Is this higher order polymeric ring continuous around the wound? Based on the 3D reconstructions (S3A) that does not appear to be the case. How is it expected that a discontinuous ring could apply membrane tension?
C. Please discuss your findings in the context of your lab's previous work. You previously elegantly showed that large amounts of vesicles (endosomes, Golgi, etc.) move to the wound site probably to plug the lesion. Is it possible that some of these ESCRT-III puncta are on vesicles that move toward the wound site? It is well known that ESCRT-III localizes to endosomes for example.

5. Using their VPS-32 polymerization mutants, the authors claim that the defects in large-scale plasma membrane repair are primarily due to defects in membrane tension stabilization at the wound periphery. Is there a way to distinguish the role of VPS-32 in its classical role for membrane scission (which also requires ESCRT-III polymerization) and in membrane tension stabilization using these mutants? Can the authors provide additional discussion about this point and speculate as to how ESCRT-III-mediated stabilization leads to the eventual resolution and repair of large wounds?

6. The authors should describe in the text what they think their membrane tension sensor is measuring. Perhaps it measures the spatial relationship between lipid rafts and PS PIP2 domains. Is it not a concern that the lipid environment is substantially changing at the wound site? It has been well demonstrated that after wounding the membrane scrambles, there are local increases in cholesterol (potentially increasing raft domains), and the authors have previously shown that PIP2 is generated at the wound site. To confirm that there are not changes in the abundance of the two domains at the wound site, the authors should express the N-Lyn Ypet module separately from KRAS-mCherry module and confirm that there is no accumulation of one module over the other at the wound site. If there is, it indicates that the signal is coming from changes in abundance of one of the lipid environments.

7. Is the role for ESCRT-III in membrane tension stabilization specific to large plasma membrane wounds? Do focal accumulations of ESCRT-III at small wounds also stabilize membrane tensions?

8. The membrane appears to be under higher basal membrane tension in VPS-32 mutant/DN cells. Are wounds formed immediately after ablation larger larger because of the high initial membrane tension? I only see wound size 3 and 6 hours post ablation reported. If possible, please show wound size at 0 hours.

9. These punctate waves of ESCRT-III proteins migrating toward PM wounds have been previously described by Heffner et al. (doi.org/10.1101/2024.11.19.624330, see Movie #2 for example) in human cells. You could consider siting them as it shows that something similar occurs in human cancer cells.

10. It would help the reader if you explained/cited why myr::mKate accumulates at the wound site in the text.

Reviewer comments (our point-by-point responses are provided in blue text):

Reviewer #1 (Comments to the Authors (Required)):

Meng et al. report that the ESCRT-III protein VPS-32 responds to large-scale (10 μm) plasma membrane wounds in C. elegans epidermis to form large polymers that encircle the wound. VPS-32 recruitment to large wounds was substantially slower than recruitment to smaller (1 μm) wounds. Using a FRET-based sensor module the authors found that VPS-32 polymerization is required in order to restore membrane tension during repair. The requirement for VPS-32 in repair of large plasma membrane wounds was not restricted to C. elegans epidermis but was also shown to be true for wounded muscles and axons.

This is an interesting study that reveals a non-canonical function for ESCRT-III filaments. Overall the conclusions are well supported by the data. However, the authors need to address the following issues prior to eventual publication.

Response: We thank the Reviewer for the thorough and thoughtful evaluation of our manuscript. We appreciate the recognition that this work reveals a non-canonical function for ESCRT-III filaments, a finding we believe substantially advances our understanding of how cells respond to large-scale mechanical membrane injury *in vivo*.

Major points:

1. Repair of small plasma membrane wounds has previously been shown to involve ESCRT components upstream of ESCRT-III such as ALIX and TSG101. The authors need to examine whether this is the case for large wounds as well.

Response: We thank the reviewer for this important suggestion. Previous studies of plasma membrane injury in mammalian cells have demonstrated that upstream ESCRT components, including ALIX and TSG101, are required for CHMP4B/VPS-32 recruitment and repair; whether a similar upstream recruitment hierarchy operates during large-scale wound repair was unknown and represents an important mechanistic question.

To address this, we examined the roles of several conserved upstream ESCRT regulators in VPS-32 recruitment following large membrane damage. Using epidermis-specific RNAi, we depleted

alx-1 (ALIX homolog), *tsg-101* (TSG101 homolog), and *pdcd-6(M04F3.4)* (ALG-2 homolog) in animals expressing VPS-32::GFP. We found that depletion of *alx-1* or *tsg-101* significantly reduced VPS-32 accumulation at the wound periphery (revised Figure 2A and 2B), resulting in decreased recruitment of the wound-associated ESCRT-III assembly. In contrast, knockdown of *pdcd-6* had no significant effect on VPS-32 recruitment compared with control animals (revised Figure 2A and 2B).

These findings indicate that large plasma membrane wounds utilize a conserved upstream ESCRT recruitment pathway involving ALX-1 and TSG-101. Interestingly, however, PDCD-6/ALG-2 was dispensable for VPS-32 recruitment in this experiment. This observation is consistent with recent studies showing that the requirement for Ca2+-dependent ALG-2 in ESCRT recruitment varies depending on the type of membrane damage and cellular context (*1–3*). Our observation therefore suggests that although large plasma membrane wound repair utilizes core ESCRT recruitment pathways, it also exhibits distinct regulatory mechanisms, potentially reflecting the different geometric and mechanical challenges associated with micron-scale membrane disruptions.

To further address the reviewer's question, we investigated whether these upstream ESCRT-III components are themselves recruited to the wound site; we generated fluorescent reporters for PDCD-6, ALX-1, and TSG-101, and monitored their localization following wounding. We found that PDCD-6, ALX-1, and TSG-101 were all recruited to large plasma membrane wounds (revised Figure S2A and S2B). **Together with our original observations that multiple ESCRT-III subunits accumulate at large wounds, these data demonstrate that large plasma membrane injury activates a coordinated ESCRT response involving both upstream ESCRT regulators and ESCRT-III components.**

These new data have been incorporated into the revised manuscript (revised Figure 2 and Figure S2).

2. It is surprising that large plasma membrane wounds can persist for up to 60 minutes without causing cell death. The authors should investigate if there is something plugging the holes, such as glycocalyx.

Response: We thank the reviewer for raising this insightful suggestion. We agree that the ability of large plasma membrane wounds to persist for an extended period without causing immediate cell death suggests that the wound does not remain a complete open membrane rupture throughout the repair process. Indeed, the wound is likely to become progressively occluded while membrane repair proceeds.

To address the reviewer's suggestion, we examined whether glycocalyx-associated material accumulates at the wound site by staining animals with wheat germ agglutinin (WGA), which recognizes carbohydrate-rich glycoconjugates (*4*). In unwounded animals, little or no WGA signal was detected at the apical surface of epidermal cells. Following needle wounding, however, WGA-positive material accumulated specifically at the wound site. By 30 minutes after injury, the WGA signal became concentrated within the center of the wounds (revised Figure S5A). Although the molecular identity and origin of this material remain to be determined, these results are consistent with the reviewer's suggestion that carbohydrate-rich material may contribute to partial wound occlusion during repair.

To further assess whether the wound site has regained barrier function, we performed fluorescent dextran permeability assays with 150-kDa dextran. Dextran readily entered wounded cells immediately after injury. It remained detectable if we stained the wounded animals at the early stages of repair (10 min-3 h) (revised Figure S5B and S5C). Still, intracellular fluorescence progressively decreased when we stained the wounded animals over time. By 6 h after wounding, dextran was rarely detected in wounded cells (revised Figure S5B and S5C), indicating that membrane barrier function had been substantially restored. These observations demonstrate that repair of large plasma membrane wounds is a gradual process in vivo, during which permeability progressively decreases rather than being restored immediately.

These new findings complement our previous work showing that plasma membrane injury induces the recruitment of the Golgi apparatus and Golgi-derived vesicles, which assemble into a membrane patch to facilitate membrane repair through SNARE-dependent membrane fusion(*5*, *6*). In addition, the *C. elegans* epidermis is covered by a mechanically robust extracellular cuticle, which likely provides structural support following injury. Although we have not directly examined the contribution of the cuticle in the present study, this tissue architecture may help limit cell lysis

and uncontrolled release of intracellular contents after large plasma membrane rupture, thereby providing a protected environment in which the membrane repair machinery can progressively restore plasma membrane integrity over several hours.

Taken together, the newly **identified accumulation of WGA-positive material**, the previously characterized membrane patch, and the progressive restoration of membrane barrier function, and the protective extracellular environment of the epidermis support a model in which large plasma membrane wounds become progressively occluded during repair rather than remaining as open membrane ruptures. Based on these new data, we have revised our working model to propose that the wound-associated ESCRT-III assembly stabilizes the wound edge, promotes recruitment of intracellular membrane compartments, and cooperates with vesicle-mediated membrane addition to facilitate progressive wound closure.

These new data have been incorporated into the revised manuscript (revised Figure 5 and revised Figure S5).

Minor point:

The research literature is in general cited adequately, but in the fast-moving ESCRT field it seems strange to cite reviews from 2006, 2011 and 2013. The authors should either cite newer reviews or refer directly to the relevant original articles.

Response: We thank the reviewer for this helpful suggestion. We agree that several of the review articles cited in the original manuscript are no longer the most appropriate references for describing the current understanding of ESCRT biology. We have therefore systematically reviewed and updated the citation list throughout the manuscript.

Specifically, we have replaced or supplemented older review articles with more recent reviews that better reflect current advances in the field, including Burigotto and Carlton, *Nature Reviews Molecular Cell Biology* (2026) (*7*), Hurley et al. Nature (2025) (*8*), Vietri et al. Nature Reviews Molecular Cell Biology (2020) (*9*), Olmos Membranes (2022) (*10*), and McCullough et al. Annual Review of Cell and Developmental Biology (2018) (*11*). We have also incorporated several recent studies published after the original submission, including Goul et al. Nature (2026) (*12*) and Heffner et al. (2026) Dev Cell (*13*), where relevant.

These updates have been implemented throughout the Introduction, Results, and Discussion sections to ensure that the manuscript more accurately reflects the current state of the ESCRT field and appropriately cites the original experimental literature.

Reviewer #2 (Comments to the Authors (Required)):

In this manuscript, Meng et al. investigated the recruitment and function of VPS32/CHMP4B in plasma membrane repair of the C. elegans hyp7 epidermis. Most previous ESCRT-III mediated membrane repair studies have been carried out in cultured cells, so it is exciting to see VPS32 recruitment and membrane repair investigated in a living organism. By combining elegant reporters, genetics, timelapse imaging and different types of membrane damage, the authors clearly show that plasma membrane wounds recruit VPS32 and that this recruitment is required for efficient repair.

However, I find the mechanistic interpretation of how VPS32 mediates repair not fully convincing at this stage, and the upstream recruitment mechanism as well as the role of other ESCRTIII subunits remain largely unexplored. This makes the work perhaps less novel than it should be. I would suggest that the authors address the following key points to strengthen their claims:

Response: We thank the reviewer for the thoughtful and detailed evaluation of our manuscript, and for the positive recognition of the in vivo approach, the combination of live imaging, genetics, and biophysical measurements, and the clear demonstration of VPS-32 recruitment and its requirement for efficient repair. We are encouraged that the reviewer finds the study exciting and the core observations convincing.

Major points

Evidence for a "supramolecular polymer" encircling the wound: Instead of invoking the canonical ESCRTIII role in neck fission/budding, the authors propose that VPS32 assembles into a massive supramolecular polymer that forms a ring around large wound. At present, there is no direct evidence for this central claim. In all VPS32 images presented, one mainly sees individual puncta that cluster and appear to accumulate/tether around the wound; continuous ringlike structures are not clearly resolved. This phenotype could also be explained by a canonical "vesicle-vesicle fusion" model of plasma membrane repair, in which multiple VPS32-positive vesicles dock and fuse near the wound.

Response: We thank the reviewer for these thoughtful and constructive comments. We agree that our current imaging does not provide definitive ultrastructural evidence for a continuous ESCRT-

III polymer encircling the wound. In both conventional confocal and multi-SIM images, VPS-32 is primarily observed as clustered puncta that accumulate around the wound margin, and a continuous closed ring cannot be resolved at the available resolution. We also agree that a vesicle-mediated repair mechanism is a plausible alternative interpretation and should be considered explicitly.

Accordingly, we have substantially revised the manuscript to moderate our conclusions. Throughout the revised text, we have replaced terms such as "supramolecular polymer ring" with more conservative descriptions "wound-associated ESCRT-III assembly" or "circumferential ESCRT-III structure," and we now explicitly acknowledge that the precise ultrastructural organization of this assembly remains to be determined.

Although we cannot directly demonstrate a continuous ESCRT-III structure, several independent observations indicate that the wound-associated ESCRT-III assembly represents more than simply a collection of docking vesicles at wound sites.

First, multi-SIM imaging revealed that VPS-32 at large wounds assembles into extended linear and spiral-like filamentous structures spanning several micrometers, in contrast to the transient punctate accumulations observed at small wounds (Figure 4A and Figure S4A-S4B). Although these filamentous structures are not uniformly continuous around the entire wound, they demonstrate that VPS-32 assembles into higher-order, membrane-associated architectures rather than remaining exclusively as isolated puncta.

Second, our new FRAP experiment shows that VPS-32 associated with large wounds exhibits markedly slower turnover than VPS-32 at small wounds (revised Figure 3G and 3H). This reduced turnover indicates the formation of a persistent, dynamically remodeled ESCRT-III assembly rather than a rapidly exchanging population of membrane-associated proteins.

To directly address the reviewer's proposed vesicle-fusion model, we performed additional analyses of vesicle trafficking during wound repair. We found that multiple vesicle markers, including the Golgi marker MANS-2, Golgi-derived vesicle marker RAB-6.2, early endosome marker EEA-1, and late endosome marker RAB-7, were all robustly recruited to wound sites following injury (revised Figure S5D-S5H), confirming extensive activation of membrane

trafficking during repair. However, none of these vesicle markers showed substantial colocalization with VPS-32 (revised Figure S5D-S5H), indicating that the wound-associated VPS-32 assembly is not simply marking the same vesicle population.

Furthermore, RNAi depletion of *rab-5*, *rab-7*, or *rab-10*, or disruption of Golgi trafficking with Brefeldin A, did not significantly impair VPS-32 recruitment to the wound margin (revised Figure S5I and S5J). In contrast, VPS-32 polymerization-defective *vps-32(E94K)* and *vps-32(V44W)* mutants exhibited significantly reduced RAB-6.2, RAB-7 or EEA-1 recruitment (revised Figure 5A-5F). Moreover, VPS-32 is also required for the recruitment of membrane fusion machinery, including SYX-2, RIC-4, and SEC-22 (revised Figure 5G-5L). Together, these findings suggest that VPS-32 assembly occurs largely independently of canonical vesicle trafficking, whereas efficient recruitment of intracellular membrane compartments and membrane fusion machinery depend on ESCRT-III.

Taken together, these newly collected data suggest that the wound-associated ESCRT-III assembly and vesicle-mediated membrane repair are not mutually exclusive mechanisms. Instead, **our results support an integrated model in which ESCRT-III assembles into a persistent, higher-order wound-associated structure that stabilizes the wound edge and restores membrane tension, thereby facilitating efficient recruitment of intracellular compartments and membrane fusion during repair of large membrane wounds.** We have revised both the Results and Discussion accordingly to present this integrated model more explicitly while acknowledging that the precise ultrastructural organization of the wound-associated ESCRT-III assembly will require future investigation.

Finally, we sincerely thank the reviewer for this constructive suggestion. This prompted us to perform substantial additional experiments on vesicle trafficking and to refine both our interpretation and working model, which we believe have significantly strengthened the manuscript.

It is not sufficient to define a "supramolecular polymer" solely from diffraction-limited fluorescence puncta. Because hyp7 epidermal cells and their wounds are very large, this system would be ideal to address this question more directly by CLEM / electron tomography to visualise

ESCRTIII assemblies at the wound edge. Alternatively, an in vitro VPS32 self-assembly assay on giant vesicles could provide more direct evidence for large continuous polymers encircling the wound.

Response: We thank the reviewer for this thoughtful and constructive suggestion. We fully agree that direct ultrastructural visualization of the wound-associated ESCRT-III assembly would provide important mechanistic insight and further strengthen our study.

To directly address this question, we performed cryo-correlative light and electron microscopy (cryo-CLEM) combined with Serialized On-grid Lift-In for Tomography (SOLIST), cryo-focused ion beam (cryo-FIB) milling, and cryo-electron tomography (cryo-ET) in collaboration with Dr. Shujun Cai's laboratory at Southern University of Science and Technology. Cryo-ET allows direct visualization of macromolecules at molecular resolution while preserving cellular ultrastructure in a near-native state. Cryo-confocal imaging of vitrified animals expressing the plasma membrane marker myr::mKate2 enabled identification of wounded regions for targeted cryo-lift-out. Cryo-ET revealed extensive membrane remodeling at the wound margin, together with ~4 nm-thick filamentous densities closely associated with the damaged plasma membrane (response Figure 1). The thickness of these filamentous densities is consistent with that of the filamentous strands (~4.1 nm) observed in the cryo-EM structure of the yeast ESCRT-III protein Snf7 (the homolog of *C. elegans* VPS-32) spirals(*15*). **Moreover, these filamentous densities are clearly thinner than the actin filaments present along the wound edge, which are measured at ~7 nm in diameter, making it unlikely that they represent F-actin.**

However, we cannot conclusively assign these filamentous densities as VPS-32 polymers. In situ structural determination of VPS-32 remains technically challenging because of its relatively small molecular size and low cellular abundance, while the spatial resolution of cryo-confocal imaging is insufficient for precise molecular targeting within cryo-tomograms. Furthermore, currently available labeling strategies, including immunogold labeling and genetically encoded electron-dense tags, are incompatible with our cryo-ET sample preparation workflow. Thus, unambiguous molecular identification of these filamentous densities was not possible. **We therefore believe that it would be inappropriate to draw definitive conclusions from these cryo-ET observations and have not included these data in the revised manuscript.**

We also appreciate the reviewer's suggestion to perform an in vitro reconstitution assay using purified VPS-32 on giant unilamellar vesicles (GUVs). We agree that such experiments would provide valuable information regarding the intrinsic self-assembly properties of VPS-32 and the architecture of ESCRT-III polymers. However, we believe that this represents a distinct line of investigation that extends beyond the scope of the present study. Our work is focused on defining how ESCRT-III functions during large plasma membrane repair in vivo, where its assembly is regulated by upstream ESCRT components, membrane tension, and intracellular membrane trafficking. Although a minimal GUV system would be highly informative for defining the intrinsic biophysical properties of VPS-32, it would not recapitulate the complex cellular environment that governs ESCRT-III assembly during physiological wound repair.

Although we cannot directly visualize the underlying architecture, several independent observations support the existence of a higher-order ESCRT-III assembly at large wounds. Super-resolution SIM imaging reveals extended linear and spiral-like VPS-32 structures at the wound periphery (Figure 4A, Figure S4A and S4B). New experiments of FRAP analysis demonstrate that these assemblies exhibit markedly slower turnover than those at small wounds (revised Figure 3G and 3H), indicating the formation of a persistent, dynamically remodeled assembly. Furthermore, polymerization-defective VPS-32 mutants fail to form the persistent wound-associated structures despite normal initial recruitment and exhibit defects in membrane tension recovery, vesicle recruitment, wound closure, and tissue repair (revised Figures 4-6). Together, these complementary imaging, dynamic, and functional data support the formation of a persistent ESCRT-III assembly during large-wound repair, while not resolving its precise ultrastructural organization.

Accordingly, we have taken a more conservative interpretation throughout the revised manuscript. We now refer to this structure as a "wound-associated ESCRT-III assembly" or "circumferential ESCRT-III structure" rather than a continuous VPS-32 polymer, and we explicitly acknowledge that its molecular architecture remains unresolved. We fully agree with the reviewer that defining the ultrastructural organization of this repair assembly is an important future direction. We are actively optimizing cryo-CLEM/cryo-ET approaches, including the integration of genetically encoded EM tags and sub-tomogram averaging, and are also interested in developing a complementary in vitro reconstitution system to further elucidate the biophysical principles

governing ESCRT-III polymerization during membrane repair. We have incorporated these considerations into the revised Discussion.

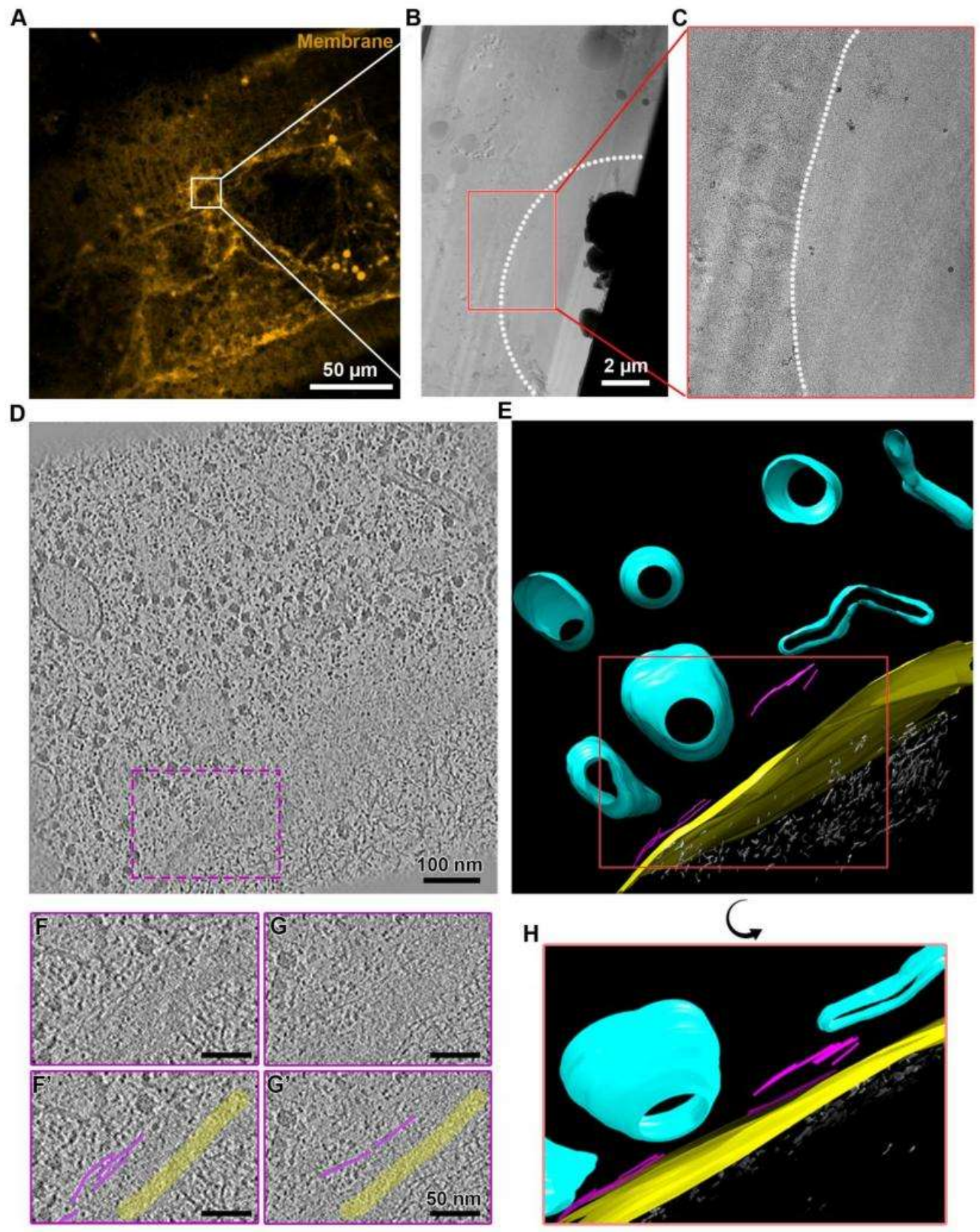

Response Figure 1. Cryo-CLEM and cryo-ET reveal membrane remodeling after large plasma membrane injury

(A) Cryo-confocal fluorescence image of a vitrified C. elegans expressing the plasma membrane marker myr::mKate2. The boxed region indicates the wounded site selected for cryo-lift-out and cryo-FIB milling.

(B) Cryo-TEM overview image of the cryo-lamella generated from the region shown in (A).

(C) Higher-magnification view of the wounded region shown in (B).

(D) Representative tomographic slice showing membrane remodeling at the wound margin.

(E) Three-dimensional segmentation of the tomogram in (D). Repairing plasma membrane, yellow; ER/microsomal membranes, cyan; actin filaments, gray; thin filamentous densities, magenta.

(F, F′) Enlarged views of the boxed region in (D), highlighting the thin filamentous densities associated with the repairing plasma membrane.

(G, G′) Enlarged views of a second region showing additional filamentous densities at the wound margin.

(H) Rotated view of the segmentation shown in (E), illustrating the close association of the thin filamentous densities with the damaged plasma membrane.

The authors show that VPS32 mutants that impair ESCRT III filament formation display much reduced recruitment. If the model is that VPS32 puncta first accumulate and then assemble into a supramolecular polymer, one might expect filament-defective mutants to still be recruited to the wound as individual puncta, but to fail in forming larger, more continuous structures. At present it is difficult to distinguish whether the mutants are defective in sensing/recruitment or in polymer assembly, because the upstream recruitment mechanism has not been characterised. A clearer separation of "recruitment" and "polymerisation" would greatly help the interpretation.

Response: We thank the reviewer for these insightful comments. We fully agree that distinguishing the initial recruitment of VPS-32 from its subsequent polymerization is critical for interpreting the mutant phenotypes. In the original manuscript, this distinction was not sufficiently clear. To address this issue, we performed additional confocal live imaging analysis of the polymerization-defective mutants *vps-32(V44W)* and *vps-32(E94K)* following large plasma membrane wounding.

These experiments revealed two mechanistically distinct phases of VPS-32 accumulation. During the early phase (0-60 min post-wounding), VPS-32(E94K) was recruited with kinetics comparable to wild-type, while VPS-32(V44W) exhibited markedly reduced recruitment to wound sites (revised Figure S4C and S4D). These results indicate that polymerization is not absolutely required for the initial recruitment of VPS-32, as demonstrated by the E94K mutant, while the V44W mutation likely affects both recruitment efficiency and subsequent assembly.

The later phase (1-3 h after wounding) revealed a much clearer distinction. In wild-type animals, VPS-32 progressively accumulated and matured into a persistent wound-associated ESCRT-III assembly. In contrast, both VPS-32(E94K) and VPS-32(V44W) failed to develop this higher-order structure. Instead, VPS-32 remained as fragmented puncta that did not mature into the extended

wound-associated assembly, as confirmed by super-resolution SIM imaging (revised Figure S4E and S4F). Thus, despite their different early recruitment phenotypes, both polymerization-defective mutants exhibited the same defect in assembly of the persistent wound-associated ESCRT-III structure.

Together, these findings demonstrate that initial recruitment and subsequent polymerization are mechanistically separable steps during ESCRT-III assembly. In particular, the VPS-32(E94K) mutant provides direct evidence that VPS-32 can be recruited to wounded membranes despite being unable to assemble into higher-order structures. Conversely, the failure of both mutants to maintain and mature the wound-associated assembly indicates that persistent accumulation at later stages depends on VPS-32 polymerization rather than initial recruitment alone.

To further clarify the upstream recruitment mechanism, we also examined conserved ESCRT regulators and found that depletion of ALX-1 or TSG-101, but not PDCD-6/ALG-2, significantly impaired VPS-32 recruitment to large plasma membrane wounds (revised Figure 2). These findings support a two-step model in which upstream ESCRT components mediate the initial recruitment of VPS-32, followed by polymerization-dependent assembly into a persistent wound-associated ESCRT-III structure. We have incorporated these new data and revised the Results and Discussion accordingly.

It is potentially very informative to understand whether and how other ESCRTIII components regulate VPS32 recruitment and repair, but the data are hard to follow. In the figures, other ESCRTIII subunits also appear as individual puncta at the wound, with different sizes/intensities compared to VPS32. Why do ESCRTIII components that are thought to act together show such distinct patterns? Please comment on this.

Response: We thank the reviewer for this important comment. We agree that the distinct puncta sizes and spatial distributions of different ESCRT-III subunits require further explanation. In the revised manuscript, we have performed additional dual-color imaging and expanded the Results section to clarify how these observations fit with the established stepwise assembly of the ESCRT-III machinery.

To directly address this question, we generated dual-color strains expressing VPS-32::tagBFP together with individual ESCRT-III subunits, including VPS-20::GFP, VPS-24::GFP, VPS-60::GFP, C01A2.4::GFP, and DID-2::GFP. Live imaging following needle wounding showed that all of these proteins are recruited to the wound region and exhibit substantial colocalization with VPS-32 (revised Figure S2F-S2K), indicating that they participate in the same wound-associated ESCRT-III assembly despite their distinct appearance in single-channel images.

Our time-lapse analyses further revealed that these subunits may be recruited with different temporal kinetics. For example, VPS-32 recruits to the wound site earlier than DID-2 (revised Figure 2E and 2F), consistent with the known hierarchical assembly of ESCRT-III. More broadly, these additional results demonstrate that large plasma membrane wounds trigger a coordinated ESCRT response involving both canonical upstream regulators and multiple ESCRT-III subunits, culminating in assembly of a wound-associated ESCRT-III structure.

Importantly, we do not expect all ESCRT-III subunits to exhibit identical localization patterns or signal intensities. Previous structural and biochemical studies have established that VPS-32 (CHMP4/Snf7) is the principal filament-forming component and likely constitutes the major structural element of ESCRT-III assemblies. In contrast, VPS-20, VPS-2, VPS-24, VPS-60, and DID-2 are thought to function at lower stoichiometry and/or at specific stages of assembly of ESCRT-III structure, including filament nucleation, stabilization, remodeling, or VPS4-mediated turnover. Consequently, differences in puncta size, abundance, and fluorescence intensity most likely reflect their distinct molecular roles and dynamic residence times within the ESCRT-III assembly, rather than indicating that they form separate or independent structures.

To improve clarity, we have incorporated the new dual-color imaging, recruitment kinetics, and colocalization analyses into revised Figure 2. We have also expanded the Discussion to explicitly describe the hierarchical and dynamic assembly of multiple ESCRT-III subunits during large plasma membrane repair.

The VPS4 part is currently confusing. For VPS4, the only apparent recruitment image seems to be Figure S5C (VPS4::mCherry, according to the legend), but the text mentions an ATPase-dead mutant, while the figure label uses "VPS4(DN)", and the red signal appears to be a membrane

marker. It is not clear which signal corresponds to VPS4. The title of Figure S2 is "VPS4 is required for VPS32 recruitment to damaged membrane", but the data supporting this statement are not clearly presented. If VPS4 is indeed required for VPS32 recruitment, this would imply that VPS32 accumulation depends on ESCRTIII complex assembly and VPS4 cycling, not only on VPS32 itself. Combined with the recruitment of other ESCRTIII subunits (Fig. 1G), one might even conclude that the proposed "supramolecular polymer" is a property of the entire ESCRTIII ensemble rather than VPS32 alone. If this is the case, is this behaviour specific to C. elegans, and how does it relate to the models derived from mammalian cells?

Response: We thank the reviewer for these thoughtful and insightful comments. We agree that the original presentation of the VPS-4 data was confusing and that our interpretation placed too much emphasis on VPS-32 rather than the ESCRT-III complex as a whole. We have revised the figures and text to clarify both the experimental data and our working model.

First, we apologize for the labeling errors in the original manuscript. The reviewer is correct that the red channel shown in the original Figure S5C corresponds to the membrane marker myr::mKate2, rather than VPS-4. This error has now been corrected, and the ATPase-defective construct is consistently designated VPS-4(DN) throughout the manuscript. We have also revised the figure legends to clearly identify each fluorescent channel and experimental condition.

Second, we have strengthened the evidence supporting the role for VPS-4 during large membrane repair. We generated a fluorescent VPS-4 reporter and found that VPS-4 is rapidly recruited to wound sites following injury, where it colocalizes with VPS-32 during assembly of the wound-associated ESCRT-III structure (revised Figure S2D and S2E). In addition, epidermis-specific depletion of *vps-4* or expression of VPS-4(DN) significantly reduced the accumulation and persistence of VPS-32 1 and 3 h post-wound at wound sites (revised Figure 2A and 2B, revised Figure S7C and S7D). Based on these new data, we have revised the Results to state more precisely that VPS-4 activity is required for efficient assembly and maintenance of the wound-associated ESCRT-III structure.

Importantly, **we fully agree with the reviewer's broader interpretation that the repair structure should not be considered a VPS-32-only assembly.** Our new dual-color imaging

demonstrates that multiple ESCRT-III components, including VPS-20, VPS-24, VPS-60, C01A2.4, and DID-2, are recruited to wound sites together with VPS-32 (revised Figure S2F-S2K). Although these subunits display distinct recruitment kinetics and relative abundances, they exhibit substantial colocalization with VPS-32, consistent with their assembly into a common ESCRT-III complex. Accordingly, we have revised the manuscript throughout to replace terms such as "VPS-32 polymer" with the more accurate and conservative terminology "wound-associated ESCRT-III assembly" or "circumferential ESCRT-III structure."

Finally, our findings are consistent with current models of ESCRT-III function in other systems. Extensive structural and biochemical studies have shown that ESCRT-III assemblies are composed of multiple subunits that polymerize sequentially and undergo continuous VPS4-dependent remodeling(*16*, *17*). Rather than functioning solely as a disassembly factor, VPS4 is now recognized as an essential regulator that drives filament remodeling, subunit exchange, and maturation of functional ESCRT-III assemblies(*7*, *11*). We therefore do not consider the wound-associated ESCRT-III assembly observed in *C. elegans* to be fundamentally species-specific. Instead, we propose that it represents an adaptation of a conserved ESCRT-III remodeling mechanism to the unique geometric and mechanical challenges posed by micron-scale plasma membrane wounds. The large size and optical accessibility of the *C. elegans* epidermis provide a unique opportunity to visualize this long-lived ESCRT-III assembly in vivo, whereas equivalent structures may be considerably more difficult to resolve in cultured mammalian cells.

To address the reviewer's concerns, we have revised the relevant figures, figure legends, Results, and Discussion to clarify the role of VPS-4, emphasize that the repair structure is a multi-component ESCRT-III assembly rather than a VPS-32-only polymer, and place our findings within the broader context of conserved ESCRT-III biology.

The idea that polymeric VPS32 acts as a scaffold to restore membrane tension at the wound margin is interesting, and the use of a membrane tension probe is a strong point. However, increased membrane tension is not unique to the "polymer ring" model. In principle, any ESCRTIII-mediated constriction/fission/budding mechanism that shrinks the wound area could also lead to a local increase in tension. The authors should acknowledge this and explain why the observed changes in tension specifically support their scaffold model rather than alternative ESCRTIII mechanisms.

Response: We thank the reviewer for this thoughtful and insightful comment. We fully agree that restoration of membrane tension is not unique to a scaffold-based mechanism. As the reviewer points out, any ESCRT-III-mediated process that progressively reduces the wound opening, including membrane constriction, budding, or fission, could contribute to local restoration of membrane tension. We have therefore revised the Discussion to explicitly acknowledge this possibility and have moderated our interpretation throughout the manuscript.

To investigate the relationship between ESCRT-III assembly and membrane mechanics, we measured membrane tension following both small (~1 μm) and large (~10 μm) laser-induced wounds using a genetically encoded FRET-based membrane tension sensor. These experiments revealed a striking size-dependent response. Small wounds recruited transient VPS-32 assemblies but did not produce a detectable decrease in membrane tension. In contrast, large wounds induced a pronounced and sustained reduction in membrane tension that gradually recovered over several hours (revised Figure 6D-6E). Importantly, this recovery was significantly impaired in animals expressing dominant-negative VPS-32 or polymerization-defective VPS-32 mutants (revised Figure 6F-6J). These findings demonstrate that VPS-32 polymerization is required for efficient restoration of membrane mechanical homeostasis during repair of large plasma membrane wounds, although they do not, by themselves, distinguish among the underlying mechanical mechanisms.

Several additional observations motivated our proposed working model. First, membrane tension began to recover while large wounds remained morphologically apparent by light microscopy, suggesting that restoration of membrane mechanics is not simply a passive consequence of complete wound closure. Second, VPS-32 formed a persistent wound-associated ESCRT-III assembly that remained associated with the wound perimeter throughout the period of mechanical recovery and exhibited substantially slower molecular turnover than the transient ESCRT-III assemblies formed at small wounds (revised Figure 3G and 3H). Finally, disruption of VPS-32 polymerization impaired membrane tension recovery, reduced vesicle recruitment, and compromised membrane remodeling without completely preventing the initial recruitment of VPS-32 to the wound site. Together, these findings suggest that higher-order ESCRT-III assembly contributes to maintaining a mechanically stable environment that supports efficient membrane repair.

Based on the reviewer's constructive comments and our newly collected data, we now propose that the persistent wound-associated ESCRT-III assembly contributes to mechanical stabilization of the wound edge while simultaneously facilitating membrane remodeling and vesicle-mediated repair. Accordingly, we have revised the manuscript to present this as a working model that is consistent with our data, while explicitly acknowledging that the precise biophysical mechanism by which ESCRT-III restores membrane tension during large plasma membrane repair remains an important question for future investigation.

In contrast to the fission/budding models proposed in mammalian ESCRT-mediated PM repair, the VPS32 puncta in C. elegans hyp7 epidermis appear more like vesicles docking and fusing with each other at the wound. This would be compatible with a canonical "vesicle-vesicle fusion" model, as described for example in sea urchin eggs and skeletal muscle repair, where Ca2+-triggered vesicles fuse to form a membrane patch. If no solid structural evidence for a continuous supramolecular polymer can be provided, the authors may need to explore this alternative repair mechanism more explicitly or at least discuss how their data could fit into a vesicle-fusion-based patch model.

Response: We thank the reviewer for this insightful comment and for highlighting the well-established vesicle-patch model of plasma membrane repair described in sea urchin eggs, skeletal muscle, and other systems. We agree that our current imaging does not provide definitive ultrastructural evidence for a continuous ESCRT-III polymer. We therefore appreciate this suggestion and have revised the manuscript to more explicitly discuss how our findings relate to vesicle-mediated membrane repair rather than presenting these mechanisms as mutually exclusive.

Our previous work has already established that vesicle-mediated membrane addition is an essential component of large plasma membrane repair in the *C. elegans* epidermis. We previously demonstrated that Golgi-derived vesicles, together with the SNARE protein SYX-2, are required for efficient wound repair(*5*, *6*), supporting a membrane-patch mechanism similar to that described in sea urchin eggs and skeletal muscle. Thus, our current study was not intended to replace the vesicle-patch model, but rather to investigate how ESCRT-III functions within this repair process.

To directly address the relationship between ESCRT-III and vesicle trafficking, we performed several additional experiments. We found that multiple intracellular membrane compartments, including Golgi-derived vesicles (MANS-2 and RAB-6.2), early endosomes (EEA-1), and late endosomes (RAB-7), were robustly recruited to wound sites following injury (revised Figure S5D-S5H), confirming extensive activation of membrane trafficking during repair. However, these vesicle markers showed little colocalization with VPS-32, indicating that the wound-associated ESCRT-III assembly is largely distinct from the vesicle populations that accumulate at the wound.

We next examined the functional relationship between these two processes. Disruption of VPS-32 polymerization significantly reduced the recruitment of Golgi-derived vesicles, endosomal compartments, and membrane fusion machinery, including SYX-2, RIC-4, and SEC-22 (revised Figure 5A-5L). In contrast, inhibition of vesicle trafficking by epidermis-specific depletion of *rab-5*, *rab-7*, or *rab-10*, or by treatment with Brefeldin A, had little effect on VPS-32 accumulation at wound sites (revised Figure S5I and S5J). These results suggest that ESCRT-III assembly occurs largely independently of canonical vesicle trafficking, whereas efficient vesicle recruitment and membrane fusion are promoted by VPS-32 polymerization.

Taken together, these findings support an integrated model in which ESCRT-III assembly and vesicle-mediated membrane repair cooperate during large plasma membrane repair. Rather than functioning as alternative mechanisms, we propose that the wound-associated ESCRT-III assembly provides a mechanically stabilized environment that facilitates the recruitment and fusion of intracellular membrane compartments, thereby promoting progressive wound occlusion and restoration of plasma membrane integrity. We have revised both the Results and Discussion to present this cooperative model more explicitly while acknowledging that the precise ultrastructural organization of the ESCRT-III assembly remains to be determined.

Minor

In several figures the labelling is confusing. Since the authors use three different types of damage, it would be very helpful to clearly label the damage type in each panel (e.g. in Fig. 1G, one had to infer the damage type by comparing scale bars and conditions). As mentioned above, Fig. S5C and the description of VPS4 are particularly confusing. Mislabelling or incomplete figure legends significantly undermines confidence in the data and should be carefully corrected throughout.

Response: We thank the reviewer for highlighting this important issue. We agree that clear and consistent figure labeling is essential, particularly because this study employs three distinct wounding paradigms: small laser ablation (~1 μm), large laser ablation (~10 μm), and needle puncture.

We have also corrected the labeling errors related to VPS-4 in revised Figure S7C, as described in our previous response. Specifically, the red channel has been correctly identified as the membrane marker myr::mKate2, the VPS-4 signal is now displayed in a separate channel, and the nomenclature VPS-4(DN) has been standardized throughout the manuscript.

In addition, we carefully reviewed all figures and legends to ensure that each panel clearly specifies the genotype, fluorescent reporter(s), wound methods, imaging time point, scale bar, and quantitative analyses where appropriate. These revisions substantially improve the clarity and consistency of data presentation and should eliminate the ambiguities identified by the reviewer.

We appreciate this suggestion, which has helped improve the overall quality and readability of the manuscript.

Late time points and disassembly. It would be valuable to know what happens at later time points: does VPS32 disassemble and revert to small puncta once repair is complete? This aspect is difficult to capture in mammalian cell systems and could be a real strength of the C. elegans model if analysed properly. A more detailed analysis and discussion of VPS32 dynamics at late time points would considerably strengthen the mechanistic insight.

Response: We thank the reviewer for this insightful suggestion. We fully agree that the ability to follow the complete lifecycle of a wound-associated ESCRT-III assembly in vivo is a unique advantage of the C. elegans epidermal wound model and can provide mechanistic insight that is difficult to obtain in mammalian cell culture systems.

To address this point, we performed long-term live imaging of VPS-32::GFP following needle-induced wounding and monitored its dynamics throughout the later stages of repair. These experiments revealed that the wound-associated ESCRT-III assembly undergoes a dynamic and reversible lifecycle consisting of assembly, persistence, and eventual disassembly. VPS-32 rapidly

accumulated at wound sites after injury, remained associated with the wound for several hours, and then gradually declined as repair progressed (revised Figure S1A-S1C and Video 4). During this process, the large wound-associated assembly progressively diminished and ultimately reverted to the small punctate distribution characteristic of unwounded epidermal cells.

Importantly, the kinetics of this response were markedly different from those observed after small plasma membrane wounds. Whereas VPS-32 recruitment to small wounds was transient and largely resolved within approximately 30 minutes, the wound-associated ESCRT-III assembly induced by large wounds persisted for at least 6 hours and remained detectable for more than 12 hours before gradually dissipating. Owing to the prolonged duration of this process and the technical limitations of continuous live imaging over extended periods, we were not able to determine the precise timing of complete disassembly. Nevertheless, our data demonstrate that the wound-associated ESCRT-III assembly is long-lived but reversible, distinguishing it from the transient ESCRT-III assemblies observed during repair of small membrane lesions.

These new findings further support our model that ESCRT-III assembly is dynamically regulated throughout the repair process. Rather than functioning as a transient repair factor, the wound-associated ESCRT-III assembly persists during the prolonged phase of membrane remodeling and mechanical recovery, before being progressively disassembled once repair is complete. We have incorporated these new data into the revised manuscript (revised Figure S1A-S1C and Video 4). We fully agree with the reviewer that defining the molecular mechanisms governing its late-stage disassembly will be an important direction for future studies, and the *C. elegans* model provides an excellent platform for addressing this question through long-term in vivo imaging.

Reviewer #3 (Comments to the Authors (Required)):

This paper investigates the assembly dynamics and function of ESCRT-III at sites of plasma membrane damage in the C. elegans epidermis. By imaging VPS-32 (CHMP-4B homolog), the authors find that after large wounding (10 µm) but not small wounding (1 µm), causes ESCRT-III to form large puncta up to 50 µm from the wound site. These puncta merge and migrate towards the wound, finally forming a ring encircling the wound site. After introducing VPS-32 mutants that have defects in polymerization, the authors show that membrane repair is defective, that the wound has slower closure rate, and that VPS-32 is recruited poorly and does not form elongated structures near the wound site. Finally, using a FRET membrane tension sensor after introduction of VPS-32 mutants, the authors conclude that ESCRT-III recovers membrane tension after PM damage by forming a massive polymer that encircles the wound site.

While this paper presents intriguing observations and ideas, it requires several revisions and controls.

Response: We thank the reviewer for the careful evaluation of our manuscript and for the thoughtful summary of our work. We appreciate the reviewer's recognition that our study identifies a wound-size-dependent ESCRT-III response during plasma membrane repair and combines live imaging, functional analyses, and membrane tension measurements to investigate its role in large-wound repair.

1. Some of the figure legends don't match the figure. See figure S2 for example.

Response: We thank the reviewer for identifying this issue and apologize for the inconsistencies in the original manuscript. We have systematically reviewed all figures and figure legends and corrected all discrepancies, including those in revised Figure S2. Each legend has been carefully revised to ensure that it accurately describes the corresponding figure, including the experimental conditions, genotypes, fluorescent reporters, and imaging parameters where appropriate.

2. Some of the citations have been added as superscript numbers. For example the citation for the specific FRET sensor used is added as citation #32, so I cannot look up what you are referencing.

Response: We thank the reviewer for bringing this formatting issue to our attention. We have carefully reviewed the citation formatting throughout the manuscript to ensure consistency and accuracy. All in-text citations now correctly correspond to the numbered reference list.

3. The videos are not titled as video #1, #2, #3, etc nor are they uploaded in a way that matches the order in the paper.

Response: We have reorganized all supplementary videos to improve consistency and ease of navigation. The videos are now labeled sequentially (Video 1, Video 2, Video 3, etc.) and are ordered according to their first appearance in the manuscript. The corresponding video citations in the text and legends have also been updated to ensure consistency throughout.

4. The model at the end is not clear. Please elaborate your model in the discussion and be more explicit about your predictions:

A. Is the idea that ESCRT-III subunits form a more static polymer structure that contain large wounds? If so, the authors should perform FRAP on VPS-32 at small vs large wound sites to show there is difference in ESCRT-III cycling at large vs small wound sites. On the other hand, if the idea is that ESCRT-III is cycling via the well-known spiral/dome mechanism, how is it thought that ESCRT-III self-associates to form these higher-order polymers? Is there precedent in vitro for these types of very large ESCRT structures?

Response: We thank the reviewer for these constructive comments. We agree that an important unresolved question is whether the wound-associated ESCRT-III structure is a static polymer or a dynamic assembly. To address this, we performed the suggested FRAP experiments and substantially revised both Results and Discussion.

FRAP analysis revealed a clear difference in VPS-32 dynamics between small and large wounds. At small wounds (~1 μm), VPS-32::GFP recovered rapidly and completely or even more rapidly after photobleaching ($t½ < 2$ min), consistent with a highly dynamic ESCRT-III assembly undergoing continuous turnover. In contrast, VPS-32::GFP at large wounds (~10 μm) recovered much more slowly ($t½$ ~20 min) and only partially during the imaging period, indicating incorporation into a longer-lived but still dynamic ESCRT-III assembly (revised Figure 3G and 3H). **Thus, the wound-associated structure is not static; rather, it exhibits substantially reduced turnover compared with the transient ESCRT-III assemblies formed at small wounds**.

To further distinguish membrane recruitment from higher-order assembly, we analyzed polymerization-defective VPS-32 mutants. As shown in the revised manuscript, VPS-32(E94K) was recruited to wound sites with kinetics similar to wild-type during the initial phase of repair but failed to mature into the persistent wound-associated assembly at later time points, whereas VPS-32(V44W) exhibited defects in both recruitment and subsequent assembly (revised Figure S4). **Together, these results indicate that initial recruitment and polymerization are mechanistically separable processes, and that formation of the persistent wound-associated ESCRT-III assembly requires VPS-32 polymerization.**

We also investigated the upstream recruitment mechanism and found that depletion of ALX-1 or TSG-101, but not ALG-2, significantly impaired VPS-32 recruitment (revised Figure 2), supporting a two-step model in which conserved upstream ESCRT factors first recruit VPS-32 to the wound, followed by polymerization-dependent maturation into a persistent ESCRT-III assembly.

Regarding the reviewer's second question, we do not propose a fundamentally new mechanism of ESCRT-III assembly. Structural and biochemical studies have established that ESCRT-III proteins intrinsically polymerize into filaments, spirals, helices, and dome-like structures on membranes, and that these polymers undergo continuous VPS4-dependent remodeling rather than remaining static(*18–20*). In vitro reconstitution experiments have further shown that ESCRT-III can assemble into extended membrane-associated polymers whose architecture is strongly influenced by membrane geometry, curvature, and mechanical constraints(*16*). We therefore propose that the large wound-associated ESCRT-III assembly observed in C. elegans represents a deployment of these well-established polymerization properties under the unique geometric and mechanical conditions created by micron-scale plasma membrane wounds, rather than a distinct mode of ESCRT-III assembly.

Accordingly, we have revised our interpretation throughout the manuscript. Rather than describing a static "supramolecular polymer," we now refer to **a persistent "wound-associated ESCRT-III assembly" or "circumferential ESCRT-III structure"**, emphasizing that it is a dynamic, continuously remodeled assembly with markedly slower turnover than canonical ESCRT-III complexes. We believe this revised model more accurately reflects both our new experimental data

and current understanding of ESCRT-III biology. We thank the reviewer again for these excellent questions, which significantly enhanced our understanding of ESCRT-III in large plasma membrane repair.

B. Is this higher order polymeric ring continuous around the wound? Based on the 3D reconstructions (S3A) that does not appear to be the case. How is it expected that a discontinuous ring could apply membrane tension?

Response: We thank the reviewer for this important observation. We agree that our 3D reconstruction does not provide sufficient evidence to conclude that the wound-associated ESCRT-III assembly forms a perfectly continuous ring. Accordingly, we have revised the terminology throughout the manuscript, replacing "continuous ring" or "supramolecular polymer ring" with the more accurate and conservative terms "wound-associated ESCRT-III assembly" or "circumferential ESCRT-III structure."

We also agree that the apparent discontinuity raises an important mechanistic question regarding how this assembly contributes to membrane stabilization. At present, our data do not support the conclusion that a fully continuous ESCRT-III polymer is required for restoring membrane tension. Instead, we favor a more cautious model in which multiple ESCRT-III assemblies are distributed around the wound perimeter and collectively contribute to stabilization of the wound edge. In this view, the relevant feature is not necessarily an uninterrupted ring, but rather the circumferential organization of ESCRT-III around the wound.

Several observations are consistent with this interpretation. First, membrane tension recovery depends on VPS-32 polymerization competence (revised Figure 6I and 6J), indicating that higher-order ESCRT-III assembly is functionally important. Second, FRAP analysis shows that these assemblies are long-lived but remain dynamic (revised Figure 3G and 3H), consistent with a persistent but remodeling assembly rather than static filaments. Third, multiple ESCRT-III components accumulate together at the wound margin (revised Figure 2D and Figure S2E-S2J), indicating that the <u>wound-associated structure is a multi-component ESCRT-III assembly rather than a VPS-32-only structure</u>.

We also recognize that the apparent discontinuity may partly reflect the current limits of light microscopy. Because the wound edge is a curved three-dimensional structure, weak or thin membrane-associated assemblies may not be continuously resolved throughout the entire perimeter. Therefore, the present data cannot distinguish conclusively between a truly discontinuous assembly and one that is more continuous than is apparent by light microscopy. Higher-resolution approaches, such as electron tomography or correlative light and electron microscopy, will be required to resolve the ultrastructure of the wound-associated ESCRT-III assembly in vivo. We attempted to pursue this experimentally, but were not able to obtain definitive structure data in the current study. We now note this explicitly as an important direction for future study.

Accordingly, we have revised both the model figure and the Discussion to distinguish our observations from our interpretation. In the revised manuscript, we propose that a persistent circumferential ESCRT-III assembly, rather than a perfectly continuous polymeric ring, contributes to stabilization of the wound edge during large-wound repair, while acknowledging that its precise ultrastructural organization remains to be determined.

C. Please discuss your findings in the context of your lab's previous work. You previously elegantly showed that large amounts of vesicles (endosomes, Golgi, etc.) move to the wound site probably to plug the lesion. Is it possible that some of these ESCRT-III puncta are on vesicles that move toward the wound site? It is well known that ESCRT-III localizes to endosomes for example.

Response: We thank the reviewer for this insightful comment. We agree that our current findings should be interpreted in the context of our previous work, which demonstrated that large plasma membrane wounds trigger robust recruitment of Golgi-derived vesicles, endosomal compartments, and SNARE-dependent membrane fusion to promote repair. Given that ESCRT-III proteins are well known to localize to endosomes, it is entirely plausible that a subset of the VPS-32 puncta observed after wounding are associated with intracellular vesicles, particularly during the early stages of the wound response.

To address this, we examined the relationship between ESCRT-III assembly and vesicle trafficking. Consistent with previous observations, the Golgi markers MANS-2 and RAB-6.2, early endosome

marker EEA-1, and late endosome marker RAB-7 were all recruited to wound sites following injury (revised Figure S5D-S5H), confirming extensive activation of intracellular membrane trafficking during repair. However, none of these markers showed substantial colocalization with VPS-32 (revised Figure S5D-S5H), indicating that the wound-associated ESCRT-III assembly is largely distinct from the vesicle populations that accumulate during repair.

We next asked whether VPS-32 polymerization contributes to vesicle recruitment. Notably, wound-induced accumulation of RAB-6.2-positive Golgi vesicles, EEA-1-positive early endosomes, and RAB-7-positive late endosomes was significantly reduced in both *vps-32(V44W)* and *vps-32(E94K)* mutants (revised Figure 5A-5F). Similarly, recruitment of the membrane fusion machinery, including the t-SNARE SYX-2, SNAP-25 homolog RIC-4, and v-SNARE SEC-22, was markedly diminished in both polymerization-defective mutants (revised Figure 5G-5L). Conversely, epidermal-specific depletion of *rab-5*, *rab-7*, or *rab-10*, as well as disruption of Golgi-dependent trafficking with Brefeldin A, had little effect on VPS-32 accumulation at wound sites (revised Figure S5I and S5J). Together, these findings support that rather than being recruited on trafficking vesicles, VPS-32 assembles into a wound-associated scaffold that subsequently promotes efficient recruitment of intracellular membrane compartments and membrane fusion machinery required for progressive wound occlusion and repair.

Based on these new data, we now favor an integrated model in which ESCRT-III-dependent mechanical stabilization and vesicle-mediated membrane addition cooperate to repair large membrane ruptures. In this model, the persistent ESCRT-III assembly stabilizes the wound edge and creates favorable mechanical conditions for efficient membrane fusion and progressive wound closure.

5. Using their VPS-32 polymerization mutants, the authors claim that the defects in large-scale plasma membrane repair are primarily due to defects in membrane tension stabilization at the wound periphery. Is there a way to distinguish the role of VPS-32 in its classical role for membrane scission (which also requires ESCRT-III polymerization) and in membrane tension stabilization using these mutants? Can the authors provide additional discussion about this point and speculate as to how ESCRT-III-mediated stabilization leads to the eventual resolution and repair of large wounds?

Response: We thank the reviewer for this important comment. We agree that the VPS-32 polymerization-defective mutants used in this study are expected to impair both the canonical membrane remodeling activities of ESCRT-III and the wound-associated stabilization function that we propose here. Therefore, these mutants do not by themselves allow a definitive functional separation between membrane scission and membrane mechanical stabilization.

To address this point more directly, we performed additional analyses of the two polymerization-defective VPS-32 mutants. These experiments revealed several informative differences and similarities.

First, the two mutants behaved differently during the early phase after wounding. VPS-32(E94K) was recruited to wound sites with kinetics broadly similar to wild-type VPS-32 during the first 60 minutes after injury, whereas VPS-32(V44W) showed markedly reduced recruitment. However, at later time points their phenotypes converged (revised Figure S4C and S4D). In contrast to wild-type VPS-32, which progressively accumulated at the wound margin and matured into a persistent wound-associated ESCRT-III assembly, neither mutant formed the characteristic circumferential structure. Instead, both remained as dispersed puncta and failed to develop into higher-order assemblies, as confirmed by super-resolution imaging at 3 hours after wounding (revised Figure S4E and S4F). These observations indicate that membrane recruitment and higher-order assembly are mechanistically separable steps: **VPS-32 can be recruited to damaged membranes without fully competent polymerization, but polymerization is required for maturation of the persistent wound-associated ESCRT-III structure**.

Second, both *vps-32(V44W)* and *vps-32(E94K)* mutant animals showed impaired wound-associated vesicle recruitment (revised Figure 5).

Third, both mutants also compromised recovery of membrane tension after large wounding (revised Figure 6 and Figure S6).

Together, these results suggest that higher-order VPS-32 assembly is required not only for the canonical membrane remodeling functions of ESCRT-III, but also for the restoration of membrane mechanical integrity during repair.

Based on these findings, we propose a working model in which ESCRT-III acts sequentially and cooperatively during repair of large plasma membrane wounds. After injury, VPS-32 is recruited to the damaged membrane and then assembles into a persistent wound-associated ESCRT-III structure through polymerization. **This higher-order assembly may help stabilize the wound edge mechanically, thereby promoting recovery of local membrane tension and creating conditions that favor vesicle recruitment, membrane addition, and progressive wound resolution**.

We have revised the Discussion accordingly to better distinguish our experimental observations from our mechanistic interpretation and to explicitly acknowledge the limits of the current mutants. The revised text now reads:

"Our results further suggest that this persistent ESCRT-III assembly serves a mechanical function that is distinct from the classical membrane scission activity of ESCRT-III. Previous studies have shown that membrane mechanics regulate ESCRT-III assembly in multiple contexts. For example, relaxation of membrane tension promotes ESCRT-III recruitment during cytokinetic abscission(*21*), while reduced membrane tension facilitates ESCRT-dependent intraluminal vesicle formation on the endosomes (*22*). During nuclear envelope repair, membrane rupture recruits ESCRT machinery to restore nuclear integrity under mechanical stress(*23–25*). Our findings extend this framework by demonstrating that ESCRT-III is not merely regulated by membrane tension but actively contributes to restoring membrane tension during repair of large plasma membrane ruptures. Notably, disruption of VPS-32 polymerization abolished this recovery without preventing the initial recruitment of VPS-32, indicating that assembly of the higher-order ESCRT-III structure, rather than simple protein accumulation, is required for mechanical stabilization of the wound edge."

6. The authors should describe in the text what they think their membrane tension sensor is measuring. Perhaps it measures the spatial relationship between lipid rafts and PS PIP2 domains. Is it not a concern that the lipid environment is substantially changing at the wound site? It has been well demonstrated that after wounding the membrane scrambles, there are local increases in cholesterol (potentially increasing raft domains), and the authors have previously shown that PIP2 is generated at the wound site. To confirm that there are not changes in the abundance of the two

domains at the wound site, the authors should express the N-Lyn Ypet module separately from KRAS-mCherry module and confirm that there is no accumulation of one module over the other at the wound site. If there is, it indicates that the signal is coming from changes in abundance of one of the lipid environments.

Response: We thank the reviewer for raising this important methodological concern and for suggesting this critical control experiment. We agree that plasma membrane injury is accompanied by extensive lipid remodeling, including phospholipid scrambling, cholesterol redistribution, and local PIP_2 accumulation, all of which could potentially influence the distribution of membrane-targeting motifs independently of membrane tension. We have now performed the suggested experiments and revised the manuscript to clarify both the sensing mechanism and the limitations of the membrane tension sensor.

The membrane tension sensor (TSM) consists of an N-terminal Lyn membrane-targeting motif and a C-terminal KRAS membrane-targeting motif connected by an elastic peptide containing a YPet–mCherry FRET pair (*26*). Under increased membrane tension, the two membrane anchors are pulled farther apart, stretching the elastic linker and reducing FRET efficiency. Conversely, membrane relaxation allows the linker to adopt a more compact conformation, resulting in increased FRET. Thus, the sensor reports changes in membrane mechanics by measuring changes in the relative distance between the two membrane anchors. We have expanded the Results section to better explain this principle.

We fully agree that wound-induced lipid remodeling could potentially alter the distribution of the Lyn and KRAS membrane anchors, thereby affecting the FRET signal independently of membrane tension. To directly address this possibility, we performed the control experiment suggested by the reviewer by expressing N-Lyn::YPet and KRAS::mCherry as separate, unfused proteins. We then quantified their relative fluorescence at wound sites throughout the repair process. Importantly, neither membrane anchor exhibited preferential enrichment or exclusion at the wound edge, and the YPet/mCherry fluorescence ratio remained unchanged relative to adjacent unwounded membrane (revised Figure S6B and S6C). These newly collected data indicate that wound-induced lipid remodeling does not differentially redistribute the two membrane-targeting motifs under our experimental conditions. Together with the close temporal correlation between FRET recovery

and restoration of membrane integrity, these findings support the conclusion that the sensor primarily reports changes in membrane mechanical tension during plasma membrane repair.

We have incorporated these new control experiments into the revised manuscript (revised Figure S6B and S6C) and expanded both the Results and Discussion to clarify the operating principle of the membrane tension sensor, the potential influence of wound-induced lipid remodeling, and the limitations of interpreting FRET measurements in this context.

7. Is the role for ESCRT-III in membrane tension stabilization specific to large plasma membrane wounds? Do focal accumulations of ESCRT-III at small wounds also stabilize membrane tensions?

Response: We thank the reviewer for this insightful question. Determining whether the membrane tension–stabilizing function of ESCRT-III is specific to large wounds or represents a general feature of plasma membrane repair is an important mechanistic issue.

To address this, we measured membrane tension using the TSM FRET sensor following both small (~1 μm) and large (~10 μm) laser-induced wounds. Following small wounding, the FRET signal remained comparable to the unwounded baseline throughout the repair process, with no significant changes detected (revised Figure 6D and 6E). In contrast, large wounds caused a rapid increase in FRET within 1 min after injury, indicating a marked reduction in membrane tension, followed by gradual recovery over the subsequent 1-3 h (revised Figure 6D-6H).

These results indicate that membrane tension is substantially perturbed only after large plasma membrane injuries. Consistent with this observation, VPS-32 displays distinct behaviors depending on wound size. At small wounds, VPS-32 forms transient assemblies that turn over rapidly (FRAP $t1/2 < 2$ min) and disappear within minutes. In contrast, large wounds induce a persistent circumferential ESCRT-III assembly with markedly slower turnover (FRAP $t1/2 \sim 20$ min), coinciding with the prolonged period of membrane tension recovery.

Together, these findings suggest that ESCRT-III performs distinct functions depending on the extent of membrane damage. During repair of small wounds, ESCRT-III forms highly dynamic assemblies consistent with its established membrane remodeling activities, while no measurable membrane tension defect is detected. In contrast, large wounds generate a sustained loss of

membrane tension that correlates with the formation of a longer-lived ESCRT-III assembly and VPS-32-dependent tension recovery. These observations support the idea that the membrane tension restoration function of ESCRT-III is preferentially engaged during repair of large plasma membrane wounds.

We have incorporated these new data into revised Figure 6 and Figure S6 and expanded the Results and Discussion to explicitly discuss the wound size-dependent functions of ESCRT-III during plasma membrane repair.

8. The membrane appears to be under higher basal membrane tension in VPS-32 mutant/DN cells. Are wounds formed immediately after ablation larger because of the high initial membrane tension? I only see wound size 3 and 6 hours post ablation reported. If possible, please show wound size at 0 hours. (5 minutes)

Response: We thank the reviewer for this insightful comment. This prompted us to examine whether impaired VPS-32 function alters the initial size of plasma membrane wounds, an important control for interpreting the delayed wound closure phenotype.

To directly address the reviewer's question, we quantified wound diameter immediately after needle wounding (5 min) in wild-type, *vps-32(V44W)*, and *vps-32(E94K)* animals. We found no significant difference in the initial wound size between WT and mutants (revised Figure 6I and 6J, and S6H). Thus, impaired VPS-32 function does not affect the size of the primary lesion.

In contrast, significant differences emerged during the repair process. Whereas wild-type wounds progressively decreased in size, wounds in VPS-32 polymerization mutants remained substantially larger at later time points, demonstrating that the repair defect reflects impaired wound closure rather than a larger initial injury.

These new data have been incorporated into revised Figure S6, which now includes wound measurements at 5 minutes, 1, and 3 h after injury, providing a complete time course of wound repair across all genotypes (revised Figure S6H).

9. These punctate waves of ESCRT-III proteins migrating toward PM wounds have been previously described by Heffner et al. (doi.org/10.1101/2024.11.19.624330, see Movie #2 for example) in human cells. You could consider citing them as it shows that something similar occurs in human cancer cells.

Response: We thank the reviewer for bringing this important study to our attention. We have now cited Heffner et al., Dev Cell (2026) (*13*) in the revised manuscript.

10. It would help the reader if you explained/cited why myr::mKate accumulates at the wound site in the text.

Response: We thank the reviewer for this helpful suggestion. myr::mKate2 is a membrane-targeted fluorescent reporter that labels the plasma membrane. Its progressive enrichment at the wound margin reflects active membrane remodeling during repair, which is driven by the recruitment and fusion of intracellular membrane compartments with the damaged plasma membrane. This interpretation is supported by our previous studies demonstrating that plasma membrane injury triggers the rapid recruitment of the Golgi apparatus and Golgi-derived vesicles, followed by SNARE-dependent membrane fusion to form a membrane patch at the wound site (*5*, *6*).

To clarify this point, we have revised the Results section where myr::mKate2 is first introduced (revised Figure 3) to explain that its accumulation at the wound periphery is consistent with ongoing membrane remodeling and membrane addition during large plasma membrane repair.

References:

1. M. L. Skowyra, P. H. Schlesinger, T. V. Naismith, P. I. Hanson, Triggered recruitment of ESCRT machinery promotes endolysosomal repair. *Science* **360** (2018).

2. W. Chen, M. M. Motsinger, J. Li, K. P. Bohannon, P. I. Hanson, Ca2+-sensor ALG-2 engages ESCRTs to enhance lysosomal membrane resilience to osmotic stress. *Proc. Natl. Acad. Sci.* **121**, e2318412121 (2024).

3. D. P. Corkery, D. Wijayatunga, B. K. L. Feron, L. K. Herzog, A. Knyazeva, Y.-W. Wu, The ATG8 E3-like ligases sense lysosomal damage and initiate ESCRT-mediated membrane repair. *EMBO J.* **45**, 930–952 (2026).

4. S. Natsuka, M. Kawaguchi, Y. Wada, A. Ichikawa, K. Ikura, S. Hase, Characterization of Wheat Germ Agglutinin Ligand on Soluble Glycoproteins in Caenorhabditis elegans. *J. Biochem.* **138**, 209–213 (2005).

5. Q. Shao, C. S. Wijaya, S. Wang, X. Meng, C. Yuan, C. Ma, S. Xu, The SNARE complex formed by RIC-4/SEC-22/SYX-2 promotes C. elegans epidermal wound healing. *Cell Rep.* **42**, 113349 (2023).

6. X. Meng, C. S. Wijaya, Q. Shao, S. Xu, Triggered Golgi membrane enrichment promotes PtdIns(4,5)P2 generation for plasma membrane repair. *J. Cell Biol.* **222**, e202303017 (2023).

7. M. Burigotto, J. G. Carlton, ESCRT-III function in membrane fission and repair. *Nat. Rev. Mol. Cell Biol.* **27**, 297–315 (2026).

8. J. H. Hurley, A. N. Coyne, M. Miączyńska, H. Stenmark, The expanding repertoire of ESCRT functions in cell biology and disease. *Nature* **642**, 877–888 (2025).

9. M. Vietri, M. Radulovic, H. Stenmark, The many functions of ESCRTs. *Nat. Rev. Mol. Cell Biol.* **21**, 25–42 (2020).

10. Y. Olmos, The ESCRT Machinery: Remodeling, Repairing, and Sealing Membranes. *Membranes* **12**, 633 (2022).

11. J. McCullough, A. Frost, W. I. Sundquist, Structures, Functions, and Dynamics of ESCRT-III/Vps4 Membrane Remodeling and Fission Complexes. *Annu. Rev. Cell Dev. Biol.* **34**, 85–109 (2018).

12. C. S. Goul, A. Jain, S. Yitiz, Z. E. Soltani, S. Yang, S. Rapp, M. Spacci, S. Federman, J. Sacco, H. Li, L. D. Enriquez, N. Liv, L. Przybyla, R. Zoncu, LASER couples damage sensing to ESCRT assembly for lysosome repair. *Nature*, 1–11 (2026).

13. C. M. Heffner, G. P. Starling, L. C. Straker, P. C. Hawes, A. M. Isaacs, J. G. Carlton, The ALS- and FTD-associated proteins annexin A11 and CHMP2B act sequentially in plasma membrane repair. *Dev. Cell*, doi: 10.1016/j.devcel.2026.05.014 (2026).

14. X. Huang, J. Zhang, C. Xu, R. Cao, P. Jiang, X. Ji, W. Wang, Z. Huang, P. Han, Vps4a Mediates a Unified Membrane Repair Machinery to Attenuate Ischemia/Reperfusion Injury. *Circ. Res.* **136**, 279–296 (2025).

15. M. Liu, Y. Liu, T. Song, L. Yang, L. Qi, Y.-Z. Zhang, Y. Wang, Q.-T. Shen, Three-dimensional architecture of ESCRT-III flat spirals on the membrane. *Proc. Natl. Acad. Sci.* **121**, e2319115121 (2024).

16. A.-K. Pfitzner, V. Mercier, X. Jiang, J. M. von Filseck, B. Baum, A. Šarić, A. Roux, An ESCRT-III Polymerization Sequence Drives Membrane Deformation and Fission. *Cell* **182**, 1140-1155.e18 (2020).

17. B. E. Mierzwa, N. Chiaruttini, L. Redondo-Morata, J. M. von Filseck, J. König, J. Larios, I. Poser, T. Müller-Reichert, S. Scheuring, A. Roux, D. W. Gerlich, Dynamic subunit turnover in ESCRT-III assemblies is regulated by Vps4 to mediate membrane remodelling during cytokinesis. *Nat. Cell Biol.* **19**, 787–798 (2017).

18. F. C. Stempels, M. Jiang, H. M. Warner, M.-L. Moser, M. H. Janssens, S. Maassen, I. H. Nelen, R. de Boer, W. F. Jiemy, D. Knight, J. Selley, R. O'Cualain, M. V. Baranov, T. C. Q. Burgers, R. Sansevrino, D. Milovanovic, P. Heeringa, M. C. Jones, R. Vlijm, M. ter Beest, G. van den Bogaart, Giant worm-shaped ESCRT scaffolds surround actin-independent integrin clusters. *J. Cell Biol.* **222**, e202205130 (2023).

19. Q.-T. Shen, A. L. Schuh, Y. Zheng, K. Quinney, L. Wang, M. Hanna, J. C. Mitchell, M. S. Otegui, P. Ahlquist, Q. Cui, A. Audhya, Structural analysis and modeling reveals new mechanisms governing ESCRT-III spiral filament assembly. *J. Cell Biol.* **206**, 763–777 (2014).

20. S. Tang, W. M. Henne, P. P. Borbat, N. J. Buchkovich, J. H. Freed, Y. Mao, J. C. Fromme, S. D. Emr, Structural basis for activation, assembly and membrane binding of ESCRT-III Snf7 filaments. *eLife* **4**, e12548 (2015).

21. J. Lafaurie-Janvore, P. Maiuri, I. Wang, M. Pinot, J.-B. Manneville, T. Betz, M. Balland, M. Piel, ESCRT-III Assembly and Cytokinetic Abscission Are Induced by Tension Release in the Intercellular Bridge. *Science* **339**, 1625–1629 (2013).

22. V. Mercier, J. Larios, G. Molinard, A. Goujon, S. Matile, J. Gruenberg, A. Roux, Endosomal membrane tension regulates ESCRT-III-dependent intra-lumenal vesicle formation. *Nat. Cell Biol.* **22**, 947–959 (2020).

23. M. Raab, M. Gentili, H. de Belly, H.-R. Thiam, P. Vargas, A. J. Jimenez, F. Lautenschlaeger, R. Voituriez, A.-M. Lennon-Duménil, N. Manel, M. Piel, ESCRT III repairs nuclear envelope ruptures during cell migration to limit DNA damage and cell death. *Science* **352**, 359–362 (2016).

24. C. M. Denais, R. M. Gilbert, P. Isermann, A. L. McGregor, M. te Lindert, B. Weigelin, P. M. Davidson, P. Friedl, K. Wolf, J. Lammerding, Nuclear envelope rupture and repair during cancer cell migration. *Science* **352**, 353–358 (2016).

25. S. S. Wallis, L. N. Ventimiglia, E. Otigbah, E. Infante, M. A. Cuesta-Geijo, G. R. Kidiyoor, M. A. Carbajal, R. A. Fleck, M. Foiani, S. Garcia-Manyes, J. Martin-Serrano, M. Agromayor, The ESCRT machinery counteracts Nesprin-2G-mediated mechanical forces during nuclear envelope repair. *Dev. Cell* **56**, 3192-3202.e8 (2021).

26. P. Ringer, A. Weißl, A.-L. Cost, A. Freikamp, B. Sabass, A. Mehlich, M. Tramier, M. Rief, C. Grashoff, Multiplexing molecular tension sensors reveals piconewton force gradient across talin-1. *Nat. Methods* **14**, 1090–1096 (2017).

October 6, 2026

RE: JCB Manuscript #202601076R

Suhong Xu
Zhejiang University

Dear Prof. Xu:

Thank you for submitting your revised manuscript entitled "Wound-associated ESCRT-III assembly restores membrane tension in micron-scale plasma membrane repair". The reviewers all now support publication so we would be happy to publish your paper in JCB pending final revisions necessary to meet our formatting guidelines (see details below).

To avoid unnecessary delays in the acceptance and publication of your paper, please read the following information carefully.

A. MANUSCRIPT ORGANIZATION AND FORMATTING:

Full guidelines are available on our Instructions for Authors page, http://jcb.rupress.org/submission-guidelines#revised.
Submission of a paper that does not conform to JCB guidelines will delay the acceptance of your manuscript.

1) Text limits: Character count for Articles is < 40,000, not including spaces. Count includes abstract, introduction, results, discussion, and acknowledgments. Count does not include title page, figure legends, materials and methods, references, tables, or supplemental legends. [Report 20k)

2) Figures limits: Articles may have up to 10 main text figures. [Report 5 figures]

3) Figure formatting: Scale bars must be present on all microscopy images, including inset magnifications. Molecular weight or nucleic acid size markers must be included on all gel electrophoresis. Aspect ratios of images may not be altered.

4) Statistical analysis: Error bars on graphic representations of numerical data must be clearly described in the figure legend. The number of independent data points (n) represented in a graph must be indicated in the legend. Statistical methods should be explained in full in the materials and methods. For figures presenting pooled data the statistical measure should be defined in the figure legends. Please also be sure to indicate the statistical tests used in each of your experiments (either in the figure legend itself or in a separate methods section) as well as the parameters of the test (for example, if you ran a t-test, please indicate if it was one- or two-sided, etc.). Also, if you used parametric tests, please indicate if the data distribution was tested for normality (and if so, how). If not, you must state something to the effect that "Data distribution was assumed to be normal but this was not formally tested."

Please check to be sure that legends include all information about the n of the experiment and the statistical tests that were performed.

5) Abstract and title: The abstract should be no longer than 160 words and should communicate the significance of the paper for a general audience. The title should be less than 100 characters including spaces. Make the title concise but accessible to a general readership.

While your current title will be appreciated by the specialists, we do not feel that it will be accessible to a broader cell biology audience. Therefore we suggest the following title: "ESCRT-III assembly restores membrane tension in micron-scale plasma membrane repair"

6) Materials and methods: Should be comprehensive and not simply reference a previous publication for details on how an experiment was performed. Please provide full descriptions in the text for readers who may not have access to referenced manuscripts. For example, in the plasmid construction section of your methods, you state: " Single-copy insertions were made using CRISPR-Cas9, as previously described6." You must remove this and write out the full methodology. The same goes for the other places in the methods where you refer to methods "...as previously described."

7) All antibodies, cell lines, animals, and tools used in the manuscript should be described in full, including accession numbers for materials available in a public repository such as the Resource Identification Portal. Please be sure to provide the sequences for all of your primers/oligos and RNAi constructs in the materials and methods. You must also indicate in the methods the source, species, and catalog numbers (where appropriate) for all of your antibodies. Please also indicate the acquisition and quantification methods for immunoblotting/western blots.

8) Microscope image acquisition: The following information must be provided about the acquisition and processing of images:

a. Make and model of microscope
b. Type, magnification, and numerical aperture of the objective lenses
c. Temperature
d. Imaging medium
e. Fluorochromes
f. Camera make and model
g. Acquisition software
h. Any software used for image processing subsequent to data acquisition. Please include details and types of operations involved (e.g., type of deconvolution, 3D reconstitutions, surface or volume rendering, gamma adjustments, etc.).

Please be sure to add additional missing imaging details.

9) References: There is no limit to the number of references cited in a manuscript. References should be cited parenthetically in the text by author and year of publication. Abbreviate the names of journals according to PubMed.

Please change references in the text to be consistent with this formatting and arrange reference list alphabetically.

10) Supplemental materials: There are strict limits on the allowable amount of supplemental data. Articles may have up to 5 supplemental figures. At the moment, you currently have 11 such items (7 figures and 4 tables). We will therefore need for you to reduce the count a bit.. Specifically, you could combine some tables into a single supplementary table with multiple 'panels'. Please be sure to correct the callouts in the text to reflect any changes. In addition, since you only currently have 7 main display items (7 figures), you could also consider moving one or more of the supp figures to the main text. Please also note that tables, like figures, should be provided as individual, editable files. A summary of all supplemental material should appear at the end of the Materials and methods section. [Report 3 SI figures]

11) eTOC summary: A ~40-50-word summary that describes the context and significance of the findings for a general readership should be included on the title page. The statement should be written in the present tense and refer to the work in the third person.

Please add this.

12) Conflict of interest statement: JCB requires inclusion of a statement in the acknowledgements regarding competing financial interests. If no competing financial interests exist, please include the following statement: "The authors declare no competing financial interests." If competing interests are declared, please follow your statement of these competing interests with the following statement: "The authors declare no further competing financial interests."

Please add this.

13) ORCID IDs: ORCID IDs are unique identifiers allowing researchers to create a record of their various scholarly contributions in a single place. Please note that ORCID IDs are now *required* for all authors. At resubmission of your final files, please be sure to provide your ORCID ID and those of all co-authors.

14) A separate author contribution section following the Acknowledgments. All authors should be mentioned and designated by their full names. We encourage use of the CRediT nomenclature.

Please add this.

Please note that JCB now requires authors to submit Source Data used to generate figures containing gels and Western blots with all revised manuscripts. This Source Data consists of fully uncropped and unprocessed images for each gel/blot displayed in the main and supplemental figures. For assays performed using capillary electrophoresis and/or immunoassay-based detection, authors should instead provide the electropherogram graph(s) for each experiment, plotting fluorescence/chemiluminescence intensity vs. molecular weight/size. Please be sure to provide one Source Data file for each figure gels, blots, and/or capillary electrophoresis assays along with your revised manuscript files. File names for Source Data figures should be alphanumeric without any spaces or special characters (i.e., SourceDataF#, where F# refers to the associated main figure number or SourceDataFS# for those associated with Supplementary figures). For traditional gels and blots, the lanes of the gels/blots should be labeled as they are in the associated figure, the place where cropping was applied should be marked (with a box), and molecular weight/size standards should be labeled wherever possible. For capillary electrophoresis assays, each trace in the graph should be color-coded and labeled to indicate which protein, gene, or sample is being measured (please try to avoid red/green combinations to accommodate our color-blind readers).

Source Data files will be made available to reviewers during evaluation of revised manuscripts and, if your paper is eventually published in JCB, the files will be directly linked to specific figures in the published article.

Source Data Figures should be provided as individual PDF files (one file per figure). Authors should endeavor to retain a

minimum resolution of 300 dpi or pixels per inch. Please review our instructions for export from Photoshop, Illustrator, and PowerPoint here: https://rupress.org/jcb/pages/submission-guidelines#revised

Journal of Cell Biology now requires a data availability statement for all research article submissions. These statements will be published in the article directly above the Acknowledgments. The statement should address all data underlying the research presented in the manuscript. Please visit the JCB instructions for authors for guidelines and examples of statements at (https://rupress.org/jcb/pages/editorial-policies#data-availability-statement).

B. FINAL FILES:

Please upload the following materials to our online submission system. These items are required prior to acceptance. If you have any questions, contact JCB's Managing Editor, Lindsey Hollander (lhollander@rockefeller.edu).

-- An editable version of the final text (.DOC or .DOCX) is needed for copyediting (no PDFs).

-- High-resolution figure and MP4 video files: See our detailed guidelines for preparing your production-ready images, https://jcb.rupress.org/fig-vid-guidelines.

-- Cover images: If you have any striking images related to this story, we would be happy to consider them for inclusion on the journal cover. Submitted images may also be chosen for highlighting on the journal table of contents or JCB homepage carousel. Images should be uploaded as TIFF or EPS files and must be at least 300 dpi resolution.

It is JCB policy that if requested, original data images must be made available to the editors. Failure to provide original images upon request will result in unavoidable delays in publication. Please ensure that you have access to all original data images prior to final submission.

The license to publish form must be signed before your manuscript can be sent to production. A link to the license to publish form will be sent to the corresponding author only. Please take a moment to check your funder requirements before choosing the appropriate license.

Additionally, JCB encourages authors to submit a short video summary of their work. These videos are intended to convey the main messages of the study to a non-specialist, scientific audience. Think of them as an extended version of your abstract, or a short poster presentation. We encourage first authors to present the results to increase their visibility. The videos will be shared on social media to promote your work. For more detailed guidelines and tips on preparing your video, please visit https://rupress.org/jcb/pages/submission-guidelines#videoSummaries.

Thank you for your attention to these final processing requirements. Please revise and format the manuscript and upload materials within 14 days. If you need an extension for whatever reason, please let us know and we can work with you to determine a suitable revision period.

Please contact the journal office with any questions at cellbio@rockefeller.edu.

Thank you for this interesting contribution, we look forward to publishing your paper in Journal of Cell Biology.

Sincerely,

Li Yu
Monitoring Editor
Journal of Cell Biology

Therese Gerbich
Scientific Editor
Journal of Cell Biology

Reviewer #1 (Comments to the Authors (Required)):

The authors have successfully addressed the points I raised, and I am happy to recommend publication of this revised manuscript.

Reviewer #2 (Comments to the Authors (Required)):

Many thanks for the authors for addressing my comments. The manuscript has substantially improved and I recommend publication.

Reviewer #3 (Comments to the Authors (Required)):

The work is now acceptable for publication in the J. Cell Biology.
