# Supplementary material for: ESCRT-III assembly restores membrane tension in micron-scale plasma membrane repair
Source: J Cell Biol. 2026 Nov 13;226(2):e202601076. doi: 10.1083/jcb.202601076 (PMC13762749; doi:10.1083/jcb.202601076)
Supplement: Supplementary file 2 [file jcb_202601076_tables1.docx]

**Supplemental Table**

| **Reagents** | | |
| --- | --- | --- |
| **Bacterial Strains** | **Source** | **Identifier** |
| OP50 | CGC | N/A |
| HT115 | Shanghai Yuanye | N/A |
| *Trelief*^®^5α Chemically Competent Cell | Tsingke | TSC-C01 |
| DB3.1 | WEIDI | DL1040 |
| **Chemicals** |  |  |
| Agarose | Tsingke | TSJ001 |
| M5 Gel-red | Mei5 | MF079-01 |
| Ethylenediamine tetraacetic acid (EDTA) | Sigma | E9884 |
| TRIS | Life Science | 0487C397 |
| Sodium chloride | Sigma | V900058 |
| Bacto Agar | BD | 214010 |
| Bacto Peptone | BD | 211677 |
| Cholesterol | Sigma | C8667 |
| Calcium chloride dihydrate | Sigma | C7902 |
| Magnesiumsulfate heptahydrate | Sigma | M1880 |
| Potassiumphosphate monobasic | Sigma | V900041 |
| Potassium phosphate dibasic | Sigma | V900050 |
| Sodium phosphate dibasic | Sigma | V900061 |
| Isopropyl beta-D-thiogalactoside | Sigma | I5502 |
| Carbenicillin Na_2_ | INALCO | 1758-9317 |
| Tryptone | OXOID | CM0129 |
| Yeast extract | OXOID | LP0021 |
| Potassium chloride | Sigma | V900068 |
| Trypan Blue | Sigma | T6146 |
| Brefeldin A (BFA) | Sigma | 2350-15-6 |
| iF488-Wheat Germ Agglutinin | Servicebio | G1730 |
| FITC-Dextran(MW 150000) | MedChemExpress | HY-128868G |
| **Critical Commercial Assays** |  |  |
| 2X Phanta Max Master Mix | Vazyme | P525-AA |
| ClonExpress II One Step Cloning Kit | Vazyme | C112-02 |
| ClonExpress MultiS One Step Cloning Kit | Vazyme | C113-02 |
| Gateway LR clonase II | Invitrogen | 56484 |
| 10X KLD Enzyme Mix | NEB | M0554S |
| ***C. elegans* Strains** | | |
| **Genotype** | **Source** | **Strain** |
| *C. elegans* N2 Bristol | CGC | N2 |
| *rde-1(ne219)* V; P*col-19*-RDE-1(*juIs346*) I | Xu and Chisholm, 2011 | CZ14540 |
| *vps-32(E94K)(syb10582)* II | SunnyBiotech | PHX10582 |
| *vps-32(V44W)(syb10631)* II | SunnyBiotech | PHX10631 |
| P*col-19*-myr::mKate2(*zjuSi46*) IV | Meng et al., 2020 | SHX364 |
| *rde-1(ne219)* V; P*col-19*-myr::mKate2(*zjuSi46*) IV ; P*col-19*-RDE-1(*juIs346*) I | Meng et al., 2020 | SHX422 |
| P*col-19*-myr::tagBFP(*zjuSi58*) IV | This paper | SHX653 |
| P*col-19*-tagBFP::SYX-2(*zjuSi72*) IV | Meng et al., 2020 | SHX1025 |
| P*col-19*-PH::GFP(*zjuSi175*) I | Meng et al., 2023 | SHX2000 |
| P*col-19*-TSM tension sensor(*zjuSi229*) IV | This paper | SHX2429 |
| P*vps-4*-VPS-4::mKate2(*zjuSi478*) II | This paper | SHX2776 |
| P*vps-32*-VPS-32::GFP(*zjuSi429*) II | This paper | SHX2778 |
| P*col-19*-RIC-4::GFP(*zjuSi309*) I | Shao et al., 2023 | SHX3015 |
| P*col-19*-GFP::SEC-22(*zjuSi292*) I | Shao et al., 2023 | SHX3057 |
| P*col-19*-VPS-4(DN)(*zjuEx2006*) | This paper | SHX3720 |
| P*col-19*-PH::mKate2(*zjuSi327*) III; P*vps-32*-VPS-32::GFP(*zjuSi429*) II | This paper | SHX3998 |
| *rde-1(ne219)* V; P*vps-32*-VPS-32::GFP(*zjuSi429*) II; P*col-19*-RDE-1(*juIs346*) I | This paper | SHX4197 |
| P*col-19*-sfGFP1-10(*zjuSi177*) I; *vps-32::GFP11* (*zju284*) II | This paper | SHX4204 |
| P*col-19*-VPS-20::GFP(*zjuEx2367*) | This paper | SHX4269 |
| P*col-19*-VPS-2::GFP(*zjuEx2393*) | This paper | SHX4275 |
| P*col-19*-DID-2::GFP(*zjuEx2397*) | This paper | SHX4279 |
| P*col-19*-TSG-101::GFP(*zjuEx2402*) | This paper | SHX4292 |
| P*col-19*-VPS-24::GFP(*zjuEx2403*) | This paper | SHX4293 |
| P*col-19*-R12C12.5::GFP(*zjuEx2543*) | This paper | SHX4481 |
| P*col-19*-C01A2.4::GFP(*zjuEx2545*) | This paper | SHX4483 |
| P*col-19*-PDCD-6::GFP(*zjuSi435*) IV | This paper | SHX4561 |
| P*col-19*-GFP::ALX-1(*zjuSi437*) I | This paper | SHX4563 |
| P*vps-32*-VPS-32::GFP(*zjuSi443*) I | This paper | SHX4616 |
| P*col-19*-mKate2::VPS-32(*zjuEx2734*) | This paper | SHX4753 |
| P*col-19*-myr::mKate2(*zjuSi46*) IV; P*vps-32*-VPS-32::GFP(*zjuSi443*) I | This paper | SHX4759 |
| P*vps-32*-VPS-32::GFP(*zjuSi455*) II | This paper | SHX4763 |
| P*vps-32*-VPS-32::tagBFP(*zjuSi457*) IV | This paper | SHX4813 |
| P*did-2*-DID-2::GFP(*zjuSi447*)II; P*vps-32*-VPS-32::tagBFP(*zjuSi457*)IV | This paper | SHX4970 |
| P*vps-32*-VPS-32::GFP(*zjuSi443*) I; P*vps-4*-VPS-4::mKate2(*zjuSi478*) II | This paper | SHX4969 |
| P*vps-4*-VPS-4::mKate2(*zjuSi505*) II | This paper | SHX5195 |
| P*col-19*-myr::mKate2(*zjuSi46*); P*vps-32*-VPS-32::GFP(*zjuSi443*); P*col-19*-VPS-4(DN)(*zjuEx3046*) | This paper | SHX5211 |
| P*vps-32*-VPS-32::GFP(*zjuSi455*); P*col-19*-VPS-4 DN(*zjuEx3048*) | This paper | SHX5213 |
| P*vps-60*-VPS-60::GFP(*zjuEx3068*) | This paper | SHX5228 |
| P*col-19*-myr::tagBFP(*zjuSi58*) IV; P*vps-4*-VPS-4::mKate2(*zjuSi505*) II | This paper | SHX5270 |
| P*col-19*-myr::tagBFP(*zjuSi58*); P*col-19*-mKate2::VPS-32(*zjuEx3088*) | This paper | SHX5279 |
| P*col-19*-MSS tesion sensor(*zjuSi229*); P*col-19*-mKate2::VPS-32(*zjuEx3095*) | This paper | SHX5300 |
| Pcol-19-myr::tagBFP(*zjuSi58*); P*vps-32*-VPS-32(V44W)::GFP(*zjuEx3206*) | This paper | SHX5492 |
| P*col-19*-TSM tension sensor(*zjuSi229*) IV ; P*vps-32*-VPS-32::tagBFP(*zjuSi542*) V | This paper | SHX5654 |
| P*vps-32*-VPS-32(E94K)::GFP(*zjuSi571*) V | This paper | SHX5758 |
| P*vps-32*-VPS-32(V44W)::GFP(*zjuSi573*) V | This paper | SHX5760 |
| P*vps-32*-VPS-32::GFP(*zjuSi585*) V | This paper | SHX5897 |
| *vps-32(E94K)*(*syb10582*); P*col-19*-myr::tagBFP(*zjuSi58*) IV | This paper | SHX5946 |
| *vps-32(V44W)*(*syb10631*); P*col-19*-myr::tagBFP*(zjuSi58)* IV | This paper | SHX5947 |
| P*vps-32-VPS-32::GFP*(*zjuSi585*) V*; Pmec-4-*mCherry(*zjuEx3522*) | This paper | SHX6051 |
| P*vps-32*-VPS-32::GFP(*zjuSi585*); P*myo-3*-mCherry(*zjuEx3524*) | This paper | SHX6053 |
| *vps-32(V44W)*(*syb10631*)*;* P*col-19-*MSS(*zjuSi229*) IV | This paper | SHX6075 |
| *vps-32(V44W)*(*syb10631*)*;* P*mec-4-GFP*(*zdIs5*) | This paper | SHX6095 |
| *vps-32(E94K)*(*syb10582*)*;*  P*col-19-*MSS(*zjuSi229*) IV | This paper | SHX6165 |
| *vps-32(E94K)*(*syb10582*); P*col-19*-mKate2::RAB-7(*zjuSi188*) I | This paper | SHX6567 |
| *vps-32(V44W)*(*syb10631*); P*col-19*-mKate2::RAB-7(*zjuSi188*) I | This paper | SHX6568 |
| *vps-32(V44W)*(*syb10631*); P*col-19*-GFP::RAB-6.2(*zjuSi313*) I | This paper | SHX6571 |
| P*col-19-*Lyn::YPet*,* P*col-19-*mCherry::KRAS(*zjuEx3787*) | This paper | SHX6573 |
| P*col-19*-GFP::MANS-2(*zjuSi302*)II; P*vps-32*-VPS-32::tagBFP(*zjuSi457*) IV | This paper | SHX6575 |
| P*col-19*-GFP::EEA-1(*zjuSi303*)II; P*vps-32*-VPS-32::tagBFP(*zjuSi457*) IV | This paper | SHX6576 |
| P*col-19*-mKate2::RAB-7(*zjuSi188*)I; P*vps-32*-VPS-32::tagBFP(*zjuSi457*)IV | This paper | SHX6577 |
| P*col-19*-GFP::RAB-6.2(*zjuSi313*); P*vps-32*-VPS-32::tagBFP(*zjuSi457*)IV | This paper | SHX6578 |
| *vps-32(V44W)*(*syb10631*)II; P*col-19*-GFP::EEA-1(*zjuSi303*) II | This paper | SHX6601 |
| *vps-32(E94K)*(*syb10582*)II; P*col-19*-GFP::RAB-6.2(*zjuSi313*) I | This paper | SHX6604 |
| P*vps-32*-VPS-32::tagBFP(*zjuSi457*)IV; P*col-19*-C01A2.4(cDNA)::GFP(*zjuEx3840*) | This paper | SHX6613 |
| P*vps-32*-VPS-32::tagBFP(*zjuSi457*)IV; P*col-19*-VPS-24::GFP(*zjuEx3838*) | This paper | SHX6611 |
| P*vps-32*-VPS-32::tagBFP(*zjuSi457*)IV; P*col-19*-VPS-20::GFP(*zjuEx3845*) | This paper | SHX6641 |
| P*vps-32*-VPS-32::tagBFP(*zjuSi457*)IV; P*col-19*-VPS-60::GFP(linker)(*zjuEx3847*) | This paper | SHX6643 |
| *vps-32(E94K)*(*syb10582*)II; P*col-19*-GFP::EEA-1(*zjuSi303*) II | This paper | SHX6728 |
| P*col-19*-GFP::RAB-1(*zjuSi253*)II; P*vps-32*-VPS-32::tagBFP(*zjuSi457*) IV | This paper | SHX6737 |
| **Plasmid** | | |
| **DNA** | **Source** | **Identifier** |
| pCFJ201-FRT-hygr-FRT-P*col-19*-myr::tagBFP | This paper | pSX960 |
| P*col-19*-Lyn::YPet | This paper | pSX1957 |
| P*col-19*-mCherry::KRAS | This paper | pSX1958 |
| P*col-19*-VPS-2::GFP | This paper | pSX1971 |
| pCFJ201-SEC-P*col-19*-MSS TSM | This paper | pSX2053 |
| pU6-BseRI-*vps-32* sg1 | This paper | pSX2171 |
| pU6-BseRI-*vps-32* sg2 | This paper | pSX2172 |
| pU6-BseRI-BseRI-sgRNA-*vps-32* #3 | This paper | pSX2220 |
| pDEST5605-SEC-P*vps-4*-VPS-4::mKate2 | This paper | pSX2260 |
| *vps-32*-GFP11-SEC KI repair template | This paper | pSX2265 |
| pcr-8-P*col-19*-TSG-101::GFP | This paper | pSX2899 |
| pCR8-P*col-19*-VPS-24::GFP | This paper | pSX2998 |
| pCR8-P*col-19*-DID-2::GFP | This paper | pSX3001 |
| pCR8-P*col-19*-VPS-20::GFP | This paper | pSX3014 |
| pCR8-P*col-19*-VPS-4(DN) | This paper | pSX3024 |
| pCR8-P*col-19*-mKate2::VPS-32 (DN) | This paper | pSX3026 |
| pCR8-P*col-19*-R12C12.5(cDNA)::GFP | This paper | pSX3427 |
| pCR8-P*col-19*-C01A2.4(cDNA)::GFP | This paper | pSX3428 |
| pCFJ201-SEC-P*col-19*-PDCD-6::GFP(cDNA) | This paper | pSX3436 |
| pCFJ210-SEC-P*col-19*-GFP::ALX-1(cDNA) | This paper | pSX3440 |
| pCFJ210-SEC-P*vps-32*-VPS-32::GFP | This paper | pSX3502 |
| pDEST5605-SEC-P*vps-32*-VPS-32::GFP | This paper | pSX3503 |
| pCFJ210-SEC-P*vps-32*-VPS-32::tagBFP::3xFLAG(new promoter) | This paper | pSX3518 |
| pCR8-P*vps-60*-VPS-60::GFP | This paper | pSX3704 |
| pDEST883-LoxP-SEC-LoxP-P*vps-32*-VPS-32(E94K)::GFP ttTi883 ChrV | This paper | pSX4211 |
| pDEST883-LoxP-SEC-LoxP-P*vps-32*-VPS-32(V44W)::GFP ttTi883 ChrV | This paper | pSX4213 |
| pDEST883-LoxP-SEC-LoxP-P*vps-32*-VPS-32::GFP (new promoter) ttTi883 ChrV | This paper | pSX4383 |
| **Oligos** | | |
| **Oligonucleotides** | **Corresponding Plasmid No.** | **Identifier** |
| *vps-32* sg1 for  acgcgtttgcccagctttcggt | pSX2171 | ZJU3879 |
| *vps-32* sg1 rev  cgaaagctgggcaaacgcgtca | pSX2171 | ZJU3880 |
| *vps-32* sg2 for  gtatccggaagcgtgactgggt | pSX2172 | ZJU3881 |
| *vps-32* sg2 rev  ccagtcacgcttccggatacca | pSX2172 | ZJU3882 |
| *vps-32* sg3 for  aatgactaaacggaaatcgggt | pSX2220 | ZJU3930 |
| *vps-32* sg3 rev  ccgatttccgtttagtcattca | pSX2220 | ZJU3931 |
| *tsg-101* for  gaggacccttggagggtacaggatgtcggcccaccaggtacaac | pSX2899 | ZJU4772 |
| *tsg-101* rev  aaaagttcttctcctttactcatttaaataggaagaccagcagttcgtcg | pSX2899 | ZJU4773 |
| *vps-24* for  gaggacccttggagggtacaggatgggtttgttcgggaaaaagaaagac | pSX2998 | ZJU4778 |
| *vps-24* rev  aaaagttcttctcctttactcattcaatcacggagctcggcaag | pSX2998 | ZJU4779 |
| *did-2* for  gaggacccttggagggtacaggatgggagccggagaaagcag | pSX3001 | ZJU4780 |
| *did-2* rev  aaaagttcttctcctttactcattcacatgttgcgaagagcagcc | pSX3001 | ZJU4781 |
| VPS-20-GFP11 For  ggacccttggagggtacaggatgggtggaattttctcgaaaaaagag | pSX3014 | ZJU5710 |
| GFP::ALX-1 for2  cgggagcctcaggagcatcgatggctacattcggctttctcagc | pSX3440 | ZJU5473 |
| GFP::ALX-1 rev2  gatggcgatctgatgacagcgctattggaatggattggcgccac | pSX3440 | ZJU5474 |
| VPS-20::GFP11 Rev  cctgaggctcccgatgctccggcttccagagcaatcttctcg | pSX3014 | ZJU5711 |
| VPS-60::GFP11 For  ggacccttggagggtacaggatgaatcggatttttggaacaggaaag | pSX3704 | ZJU5712 |
| VPS-60::GFP11 Rev  cctgaggctcccgatgctcctgctggtaatttcggcagtcc | pSX3704 | ZJU5713 |
| *R12C12.5* for  ggacccttggagggtacaggatgcctttcaagaagatgtcgtgg | pSX3427 | ZJU5792 |
| *R12C12.5* rev  cctcccgaacctccacctccgtttgatgcggcccactgc | pSX3427 | ZJU5793 |
| *C01A2.4* for caggctccgaattcgcccttgatgatgaacgacactcttgactc | pSX3428 | ZJU5801 |
| *C01A2.4* rev  tgggtcgaattcgcccttctttgaattcatctcaattccaatttcatcg | pSX3428 | ZJU5802 |
| VPS-32(V44W) for 2  tggatcgacgaaaagcaaaatgccg | pSX4213 | ZJU6744 |
| VPS-32(V44W) rev 2  attttgcttttcgtcgatccattttttctccaagaattcttgtttcttctcaagc | pSX4213 | ZJU6745 |
| VPS-32(E94K) for 2  aaaaatgcttcaacgaatgctgaag | pSX4211 | ZJU6746 |
| VPS-32(E94K) rev 2  aacttcagcattcgttgaagcatttttgagggcttctctctgatatccg | pSX4211 | ZJU6747 |
